# Ti-based MOFs with acetic acid pendings as an efficient catalyst in the preparation of new spiropyrans with biological moieties

**DOI:** 10.1038/s41598-024-62757-x

**Published:** 2024-06-19

**Authors:** Zahra Torkashvand, Hassan Sepehrmansourie, Mohammad Ali Zolfigol, Yanlong Gu

**Affiliations:** 1 Department of Organic Chemistry, Faculty of Chemistry and Petroleum Sciences, Bu-Ali Sina University, Hamedan, 6517838683 Iran; 2 School of Chemistry and Chemical Engineering, Huazhong University of Science and Technology, 1037 Luoyu Road, Hongshan District, Wuhan, 430074 China

**Keywords:** Metal–organic frameworks (MOFs), MIL-125(Ti)-NH_2_, Porous catalyst, Spiropyran, Biologically active spiropyrans, Chemistry, Catalysis, Heterogeneous catalysis

## Abstract

The strategy of designing heterogeneous porous catalysts by a post-modification method is a smart strategy to increase the catalytic power of desired catalysts. Accordingly, in this report, metal-organic frameworks based on titanium with acetic acid pending were designed and synthesized via post-modification method. The structure of the target catalyst has been investigated using different techniques such as FT-IR, XRD, SEM, EDX, Mapping, and N_2_ adsorption/desorption (BET/the BJH) the correctness of its formation has been proven. The catalytic application of Ti-based MOFs functionalized with acetic acid was evaluated in the preparation of new spiropyrans, and the obtained results show that the catalytic performance is improved by this modification. The strategy of designing heterogeneous porous catalysts through post-modification methods presents a sophisticated approach to enhancing the catalytic efficacy of desired catalysts. In this context, our study focuses on the synthesis and characterization of metal-organic frameworks (MOFs) based on titanium, functionalized with acetic acid pendants, using a post-modification method. Various characterization techniques, including Fourier-transform infrared spectroscopy (FT-IR), X-ray diffraction (XRD), scanning electron microscopy (SEM), energy-dispersive X-ray spectroscopy (EDX), mapping, and N_2_ adsorption/desorption (BET/BJH), were employed to investigate the structure and composition of the synthesized catalyst. These techniques collectively confirmed the successful formation and structural integrity of the target catalyst. The structure of the synthesized products was confirmed by melting point, ^1^H-NMR and ^13^C-NMR and FT-IR techniques. Examining the general process of catalyst synthesis and its catalytic application shows that the mentioned modification is very useful for catalytic purposes. The presented catalyst was used in synthesis of a wide range of biologically active spiropyrans with good yields. The simultaneous presence of several biologically active cores in the synthesized products will highlight the biological properties of these compounds. The present study offers a promising insight into the rational design, synthesis, and application of task-specific porous catalysts, particularly in the context of synthesizing biologically active candidate molecules.

## Introduction

Porous materials have attracted the attention of researchers for many years. Metal-organic frameworks are an important class of porous materials. Metal-organic frameworks (MOFs) are architectural structures consisting of the bonding of metals and organic linkers^1–4^. These structures are composed of metal elements that are ionic inside or outside of their core and organic ligands that coordinate with metals^5,6^. The main advantage of MOFs is their openable three-dimensional structure and designable, which allows for changing the volume and shape of the structure^7,8^. MOFs have a complex network structure that can be used in various fields such as storage and transport of gases, separation of materials, removal of pollutants from the environment, synthetic catalysis, and pharmaceuticals^9–22^. Due to the characteristics and structural diversity of MOFs, a lot of research has been conducted on the design and synthesis of these materials as well as their applications^23–27^. MOFs based on titanium (Ti) are also available. Titanium-based MOFs include structures in which titanium is used as a central metal and organic ligands are attached to it^28,29^. One of the most famous titanium-based MOFs is MIL-125, also known as MIL-125(Ti). This MOF has a porous structure with high volume and has many applications in various fields. In addition to MIL-125(Ti), a large number of Ti-based MOFs with diverse structures and properties have been used in scientific research and industrial applications. Some other examples include UiO-66(Ti), PCN-224(Ti), and NU-1000(Ti)^30–32^. Ti-based MOFs are attractive for many novel applications due to their unique physical and chemical properties, including high-temperature compatibility, remarkable mechanical strength, and high adsorption capacity^33,34^.

Post-modification of MOFs refers to the process of modifying the structure and properties of MOFs after their initial synthesis^35,36^. Post-modification techniques offer a way to increase MOF performance and functionality by introducing additional functional groups or guest molecules into the framework. These changes can be made through a variety of chemical reactions such as substitution, addition, coordination, and covalent bonding^37^. Post-modification techniques provide a means to tailor MOFs for specific applications by customizing their properties and functionalities. These strategies have significantly expanded the scope of MOF materials, allowing them to address various challenges in areas such as energy storage, environmental remediation, and biomedical applications^38,39^. In recent years, our research group introduced various tasked-specific catalysts by applying the post-modification method on MOFs^40–51^, carbon quantum data (CQDs)^52^, mesoporous^53,54^, and organic materials such as melamine^55^, uric acid^56^, and glycoluril^57^.

**Figure 1:**
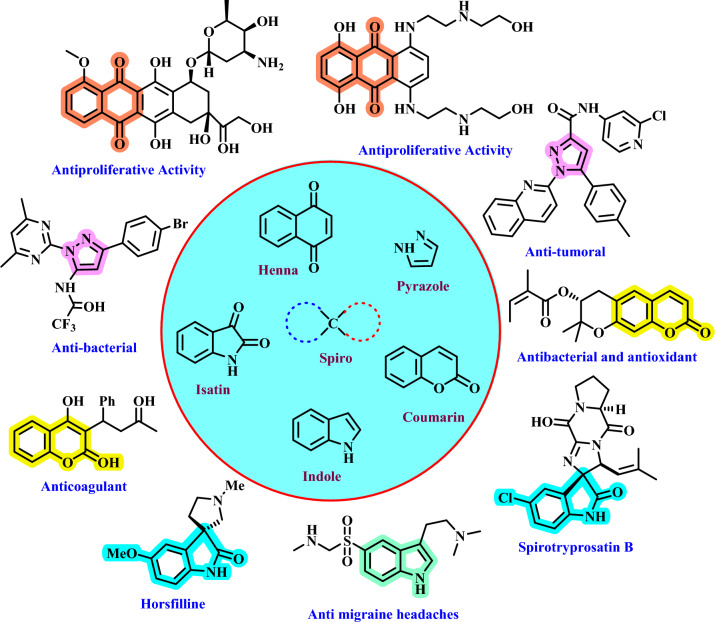
Some of the structure that have medicinal properties containing pyrazole, henna, indole, isatin, coumarin and spiro moieties.

Spiropyrans are a class of organic compounds that contain two or more rings fused at a single atom. One of the remarkable features of spiropyrans is their three-dimensional shape, which can provide structural rigidity and influence their chemical properties^58^. The presence of the spiro framework often gives these compounds unique biological activities and physical properties. Spiropyrans have various applications in medicinal chemistry, agrochemicals, materials science, and other fields. Also, they can show interesting pharmacology^59,60^. Henna, pyrazole, indole, isatin, and coumarin due to their considerable importance in medicinal chemistry have been used for spiropyrans with biologically active moieties^61,62^. Spiropyrans show diverse biological activities and have been studied for their potential therapeutic applications. For example, some derivatives have anti-inflammatory, antioxidant, antimicrobial, and anti-cancer properties. Some pharmaceutical drugs and natural products have these structures. (Figure 1)^63–66^.

In continuation of our previous investigation in the synthesis of spiropyrans^48,59, 67–73^, and 1,3,5-s- triazine derivatives^74,75^, herein due to the characteristics of Ti-based MOFs, and ability of post-modification method, we decided to design and synthesize (MOF-Ti)/TCT/Im/[CH_2_CO_2_H]Br as a porous catalyst. In the design of desired catalyst, our aim is to create acetic acid pending on MIL-125(Ti)-NH_2_ to increase its catalyst activity. The catalyst designed in this study undergoes functionalization via a post-modification method, resulting in the creation of acetic acid tags on a titanium-based metal-organic framework. The synthesized catalyst exhibits desirable activity attributed to its heterogeneity and possesses key characteristics including facile separation and recyclability, appropriate porosity, and functionalization with acetic acid groups. The catalytic activity of (MOF-Ti)/TCT/Im/[CH_2_CO_2_H]Br in the preparation of spiropyrans with biologically active moieties such as indole, pyrazole, henna, isatin and coumarin was investigated. The simultaneous presence of several biologically active cores in the synthesized products will highlight the biological properties of these compounds.

## Experimental section

### Materials and methods

2-Amino terephthalic acid (NH_2_-BDC, 95%), titanium tetraisopropanolate (TTIP, 99.8%), trichloro triazine (TCT, 95%), triethylamine (99%), imidazole (99%), Hydrobromic acid (HBr, 47%), bromoacetic acid (99%), sulfuric acid (98%), bromine (Br_2_, 99%), potassium cyanide (KCN, 98%), indole (99%), cyanoacetic acid (98%), acetic anhydride (99%), 2-hydroxy-1,4-naphthoquinone (Henna, 98%), hydrazine (N_2_H_4_, 80%), 4′-chloroacetophenone (C_8_H_7_Cl, 98%), and various ketone derivatives (95%) were purchased from Merck and Sigma-Aldrich. Furthermore, all solvents such as tetrahydrofuran (THF 99%), acetonitrile (CH_3_CN, 99%), ethanol (EtOH, 99%), ethyl acetate (EtOAc, 95%), *n*-hexane (95%),* N*,* N*-dimethylformamide (DMF, 99%), and methanol (MeOH, 99%) were purchased from Merck and Sigma-Aldrich without further purification.

### Characterization

Energy-dispersive spectroscopy (EDS) and elemental mapping were carried out by the model Oxford instruments ZEISS (England). The morphology of the obtained precursors from the different stages of the synthesis of the desired catalyst was characterized using a scanning electron microscope (SEM) technique TESCAN MIRA-II (Czechia). Meanwhile, the FT-IR technique model device) PerkinElmer spectrum version 10.02.00) was used to identify the functional groups of the different stages in the course of synthesis of desired catalyst. Furthermore, XRD patterns of the different stages of synthesized catalyst were detected by X-ray diffractometer PHILIPS PW1730 (Netherlands). Finally, Brunauer-Emmett-Teller (BET) technique with a model device BELSORP-mini-II was utilized to determine the surface area and pore size of synthesized catalyst.

### Preparation of Ti-based MOFs

MIL-125(Ti)-NH_2_ or (MOF-Ti) as a Ti-based MOFs was prepared by a solvothermal method^76,77^. To prepare this porous structure, NH_2_-BDC (6 mmol, 1.086 g) was poured into DMF (25 mL) and then MeOH (25 mL) and titanium tetraisopropanolate (TTIP) (3 mmol, 0.852 g) were added to it. The reaction mixture was stirred for 5 min at room temperature and transferred to a 60 mL autoclave. After 24 h at 150 °C, the system was cooled down to ambient temperature. The yellow product was washed several times with DMF and MeOH to separate the unreacted raw material (1.8 g product).

### Preparation of (MOF-Ti)/TCT/imidazole

To modify Ti-based MOFs, in a 50 mL flask, MIL-125(Ti)-NH_2_ (0.5 g) was mixed with trichloro triazine (TCT) (5 mmol, 0.92 g) in dry THF (30 mL) and stirred at 25 °C for 6 h under N_2_ atmosphere. The precipitate was washed with dry THF to purify it. (MOF-Ti)/TCT was dried at 80 °C. Next, in a 50 mL flask, imidazole (Im) (10 mmol, 0.68 g), and triethylamine (2 mmol, 0.2 g) were poured into 30 mL dry THF. Then, to the resulting solution, (MOF-Ti)/TCT (0.5 g) was added and stirred at 25 °C for 5 h and 24 h under reflux conditions, respectively. After the completion of the reaction, the precipitate was washed with dry THF to perform purification. (MOF-Ti)/TCT/Imidazole was dried at 80 °C^78^.

### Preparation of (MOF-Ti)/TCT/Im/[CH_2_CO_2_H]Br as a porous catalyst

**Figure 2:**
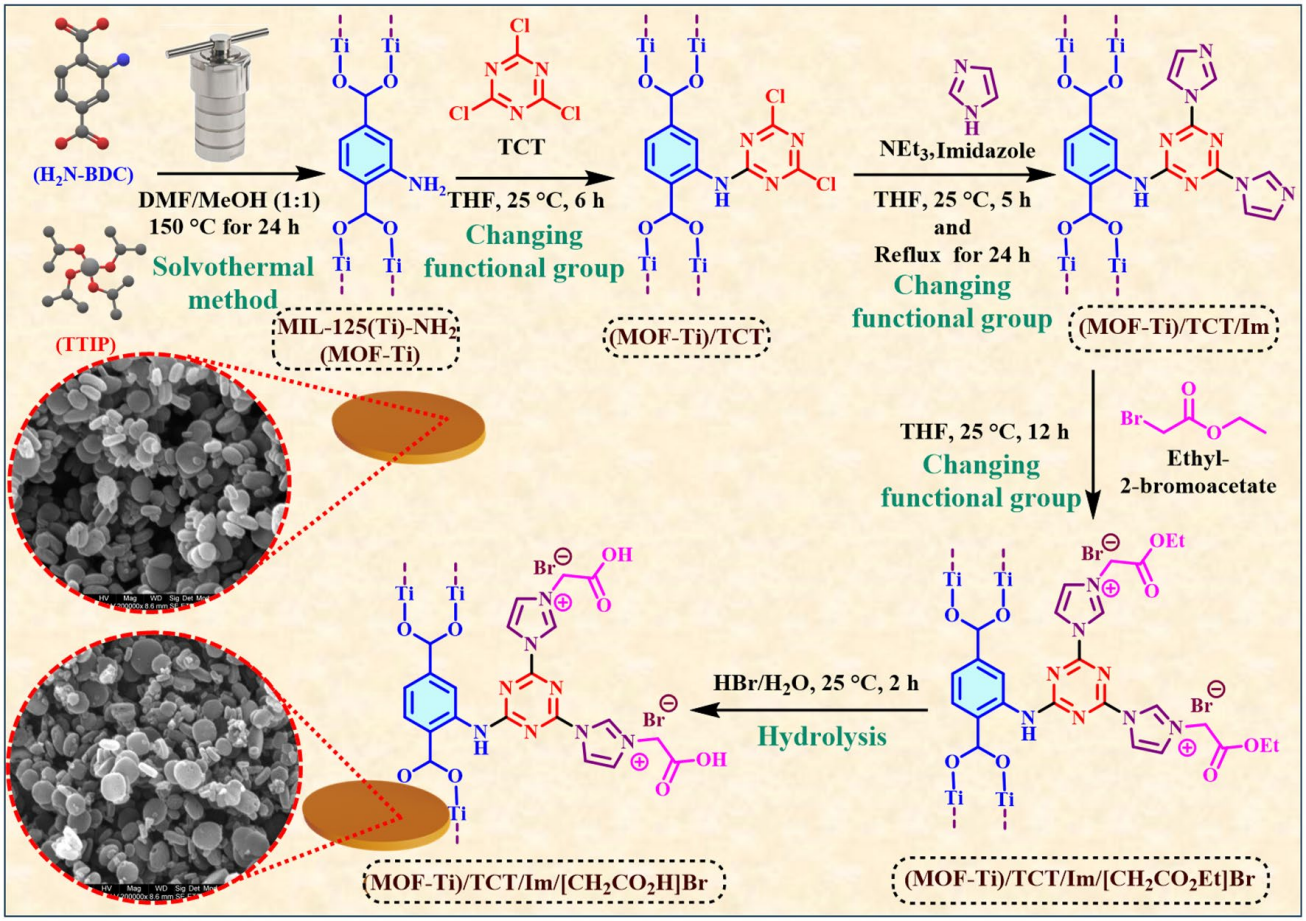
Catalyst preparation strategy for the synthesis of Ti-based MOFs with acetic acid pendings.

To synthesize the final catalyst, (MOF-Ti)/TCT/Imidazole (0.1 g) was mixed with ethyl bromoacetate (3 mmol, 0.5 g) which was synthesized according to previously reported methodology^73^) in 5 mL of dry THF and stirred for 12 h at 25 °C. After the completion of the reaction, the precipitate was purified with dry THF. After, (MOF-Ti)/TCT/Im/[CH_2_CO_2_Et]Br (0.1 g) was stirred in a 10 mL flask containing 2 mL of H_2_O and 1 mL of HBr for hydrolysis at 25 °C for 2 h. After the hydrolysis was completed, the yellow precipitate was separated and dried at 80 °C (0.12 g product) (Fig. 2).

### Catalytic reaction

**Figure 3:**
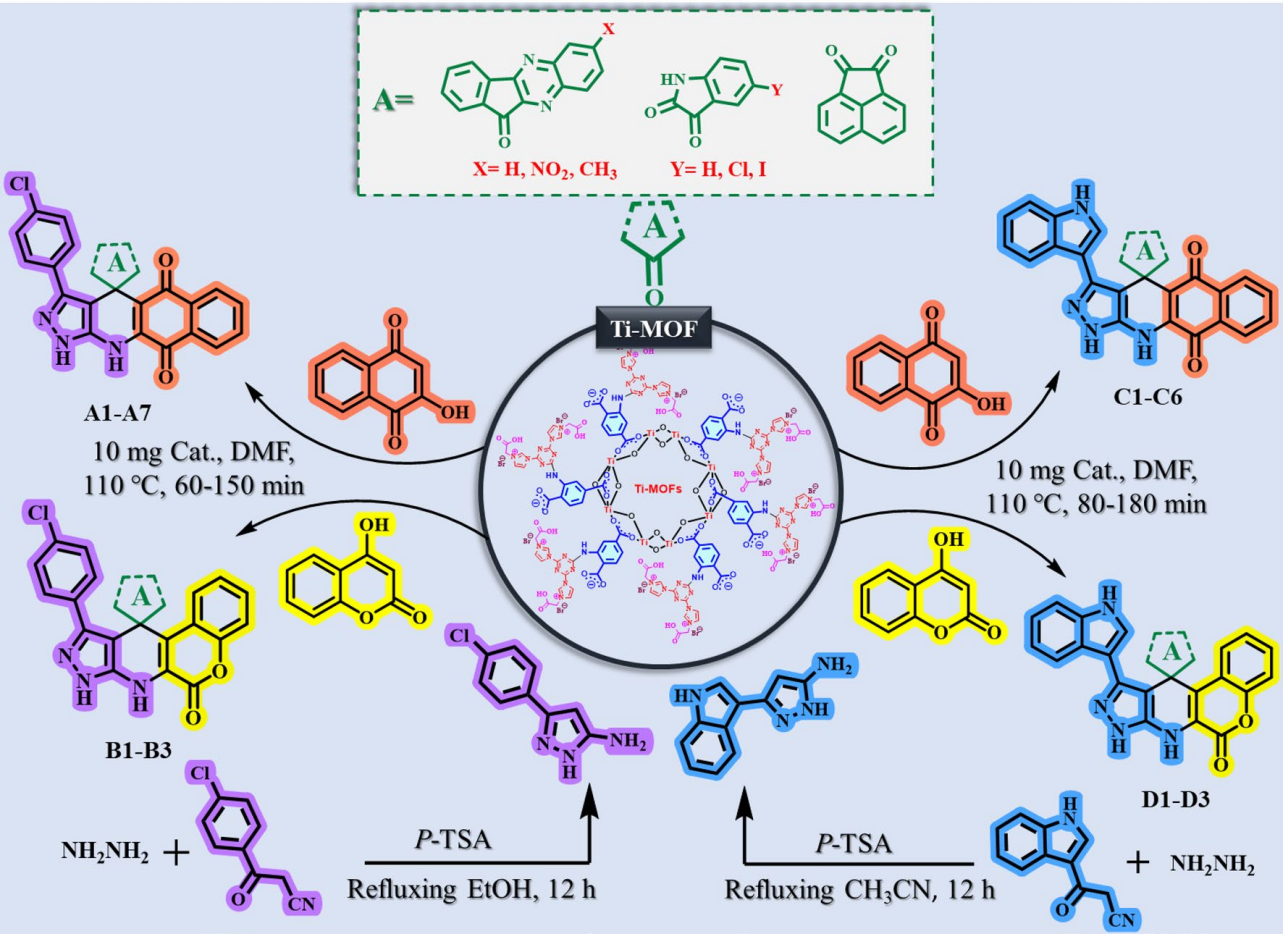
Catalytic application strategy for preparation of new spiropyrans including biological moieties using Ti-based MOFs with acetic acid pendings as a porous catalyst.

First, raw materials 3-(4-chlorophenyl)-1H-pyrazol-5-amine and 3-(1H-indol-3-yl)-1H-pyrazol-5-amine were synthesized according to the previous methods^44,48, 79–81^. Next, in a 10 mL flask, 1 mmol of raw materials, 1 mmol of ketone derivatives of category A such as isatin, 1 mmol of 2-hydroxynaphthalene-1,4-dione (Henna) or 4-hydroxy-2H-chromen-2-one, 10 mg of (MOF-Ti)/TCT/Im/[CH2CO2H]Br as a porous catalyst in 5 mL of DMF were stirred to appropriate time at 110 °C. The progress of the reaction was monitored with the help of TLC (n-Hexane: EtOAc, 4:6) technique. After the completion of the reaction, the catalyst was separated from the reaction mixture by centrifugation. Then, H2O (5 mL) was added to the reaction mixture and the precipitate was washed several times with EtOH and EtOAc solvents for purification. The resulting pure product was dried at 100 °C and identified with the help of different techniques (Fig. 3).

## Result and discussion

### Catalyst preparation strategy

Tasked-specific design of porous catalysts has been our main research interest in recent years^40–51^. Functionalizing suitable substrates for the synthesis of efficient catalysts is one of the most important ways to improve catalytic abilities. In this report, the goal is to design a catalyst based on MIL-125(Ti)-NH_2_. The reason for choosing the Ti-based MOFs is the morphology as well as the suitable surface area of this porous structure, which has been an important factor in catalytic purposes. Next, to improve the catalytic performance of this structure, the creation of acetic acid on MIL-125(Ti)-NH_2_ was used. In the first step, trichlorotriazine (TCT) and imidazole were used to modify MIL-125(Ti)-NH_2_. In the next step, ethyl bromoacetate was used for the final modification of the described porous structure. In the last step, hydrolysis of the ester functional group was done to prepare the final catalyst. The results obtained from various analyses show that the mentioned steps are well done and the structure, morphology, and surface area of (MOF-Ti)/TCT/Im/[CH_2_CO_2_H]Br are suitable for catalyzing the organic reaction. The obtained results of this report approve this claim (Fig. 2).

The catalytic application of Ti-based MOFs functionalized with acetic acid as a porous catalyst was evaluated for the synthesis of new spiropyrans containing biological moieties. Spiropyrans have been used in various fields of medicine and industry. Therefore, expanding the synthesis of such compounds is very important. The synthesized catalyst was used in the preparation of spiropyrans and very favorable results were obtained. The obtained results show that in the presence of the presented catalyst, various spiropyran derivatives can be synthesized with high yields and short reaction times. Therefore, in the design of products, it has been tried to use different isatin, ketones, amines, henna and coumarin in the preparation of these compounds (Fig. 3). The synthesized products were evaluated and identified using various techniques such as melting point, ^1^H-NMR and ^13^C-NMR (results are included in the supporting information).

**Figure 4:**
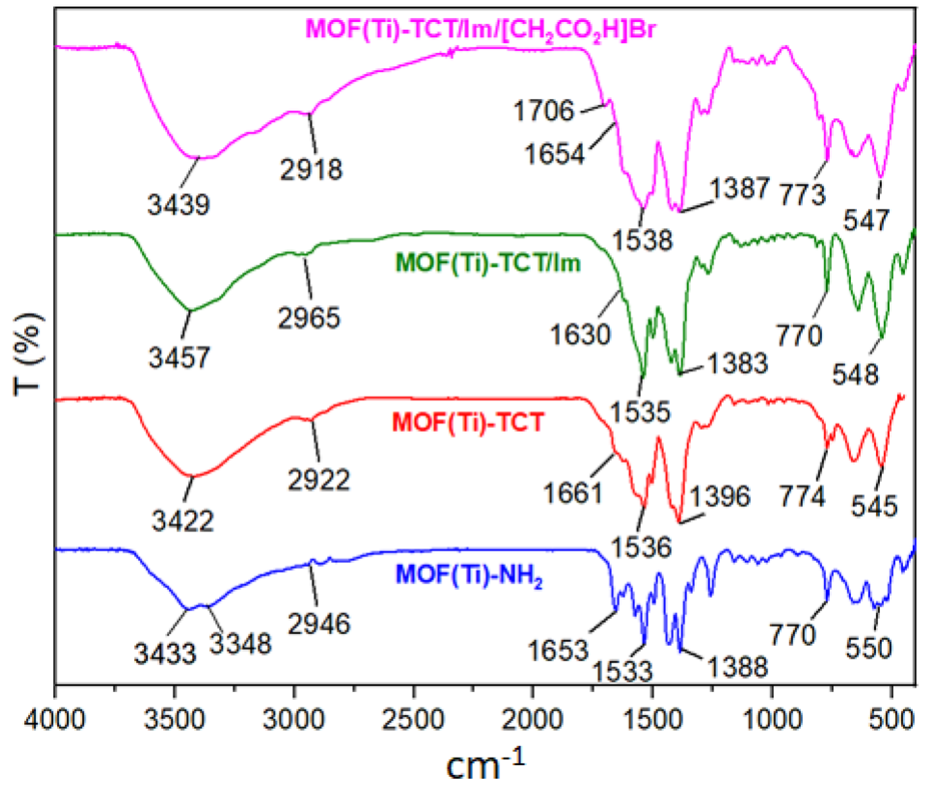
Comparison of the FT-IR pattern of different products of any stage of Ti-based MOFs which was functionalized with acetic acid as a porous catalyst.

FT-IR spectra of different stages of (MOF-Ti)/TCT/Im/[CH_2_CO_2_H]Br as a porous catalyst are compared in Fig. 4. The broad peak 2500–3600 cm^−1^ is related to the acidic OH group of acetic acid in the final catalyst. The new peak added in the area of 1706 cm^−1^ is related to the carbonyl group of acetic acid created in the final structure. The absorption peaks at 3433 and 3448 cm^−1^ indicate the symmetric and asymmetric vibrations of NH_2_ in the MIL-125(Ti)-NH_2_ structure. The absorption peak at 1653 cm^−1^ in MIL-125(Ti)-NH_2_ is assigned to the stretching of the C=O bond of the carboxylic acid group. The changes in the FT-IR spectra of different products of any stage of catalyst synthesis show that the synthesis stages have progressed well. Also, the structure of MIL-125(Ti)-NH_2_ has not been destroyed during the addition of various compounds.

**Figure 5:**
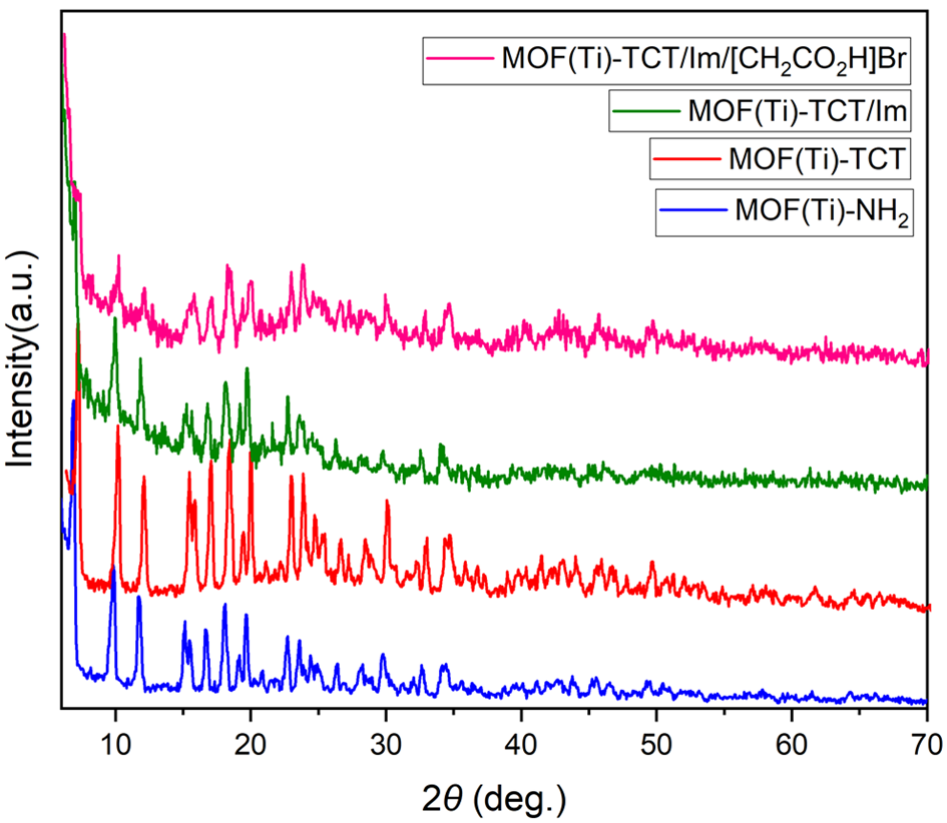
Comparison of the XRD pattern of different products of any stage of Ti-based MOFs which was functionalized with acetic acid as a porous catalyst.

XRD analysis was used to identify the crystal planes of the compounds. The XRD results related to different products of any stage of the catalyst are shown comparatively in Fig. 5. According to the obtained results, the pattern of crystal plates of MIL-125(Ti)-NH_2_ structure is consistent with previous reports^76,77^. Peaks of MIL-125(Ti)-NH_2_ exhibited 2***θ*** = 6.93, 9.60, 9.82, 11.73, 15.13, 15.53, 16.73, 18.13, 19.13, 19.73, 20.78, 22.74, 23.58, and 26.38° corresponding to diffraction lines (101), (200), (002), (211), (220), (310), (103), (222), (312), (213), (400), (004), (422), and (204). This crystal pattern is well preserved in different stages, which indicates the stability of the structure during synthesis and modification.

**Figure 6:**
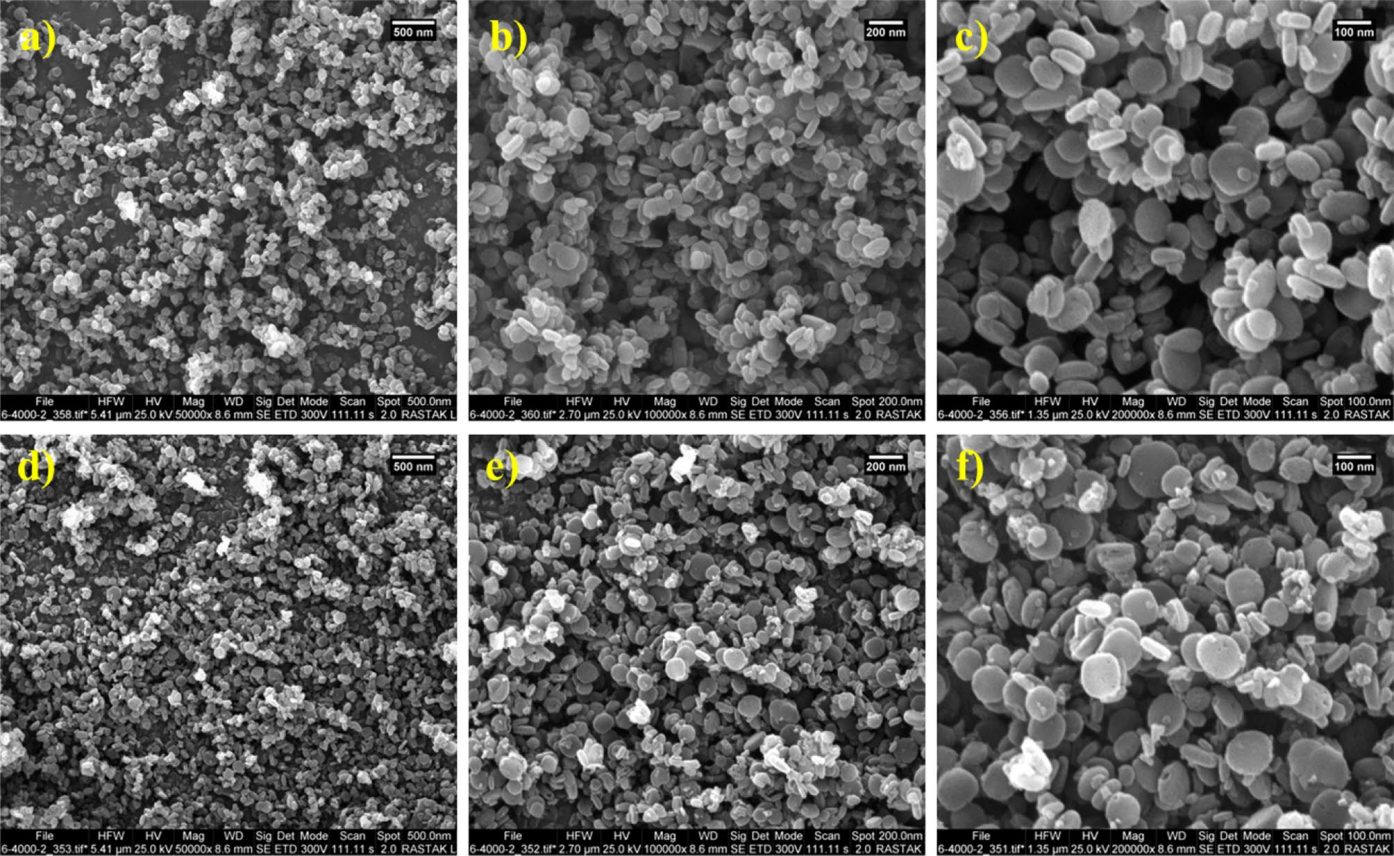
Scanning electron microscopy (SEM) of MOF(Ti)-NH_2_ (**a**–**c**) and Ti-based MOFs functionalized with acetic acid as a porous catalyst (**d**–**f**).

The morphology of metal–organic framework MIL-125(Ti)-NH_2_ and the final catalyst was examined by scanning electron microscopy (SEM) (Fig. 6). As shown in Figures, the structure morphology of MIL-125(Ti)-NH_2_ is disk-like. Also, the morphology of MIL-125(Ti)-NH_2_ remains unchanged after different stages of structure post modification. The morphology of Ti-based MOFs functionalized with acetic acid has appeared as a disk-like morphology. This type of morphology provides a suitable catalytic activity.

**Figure 7:**
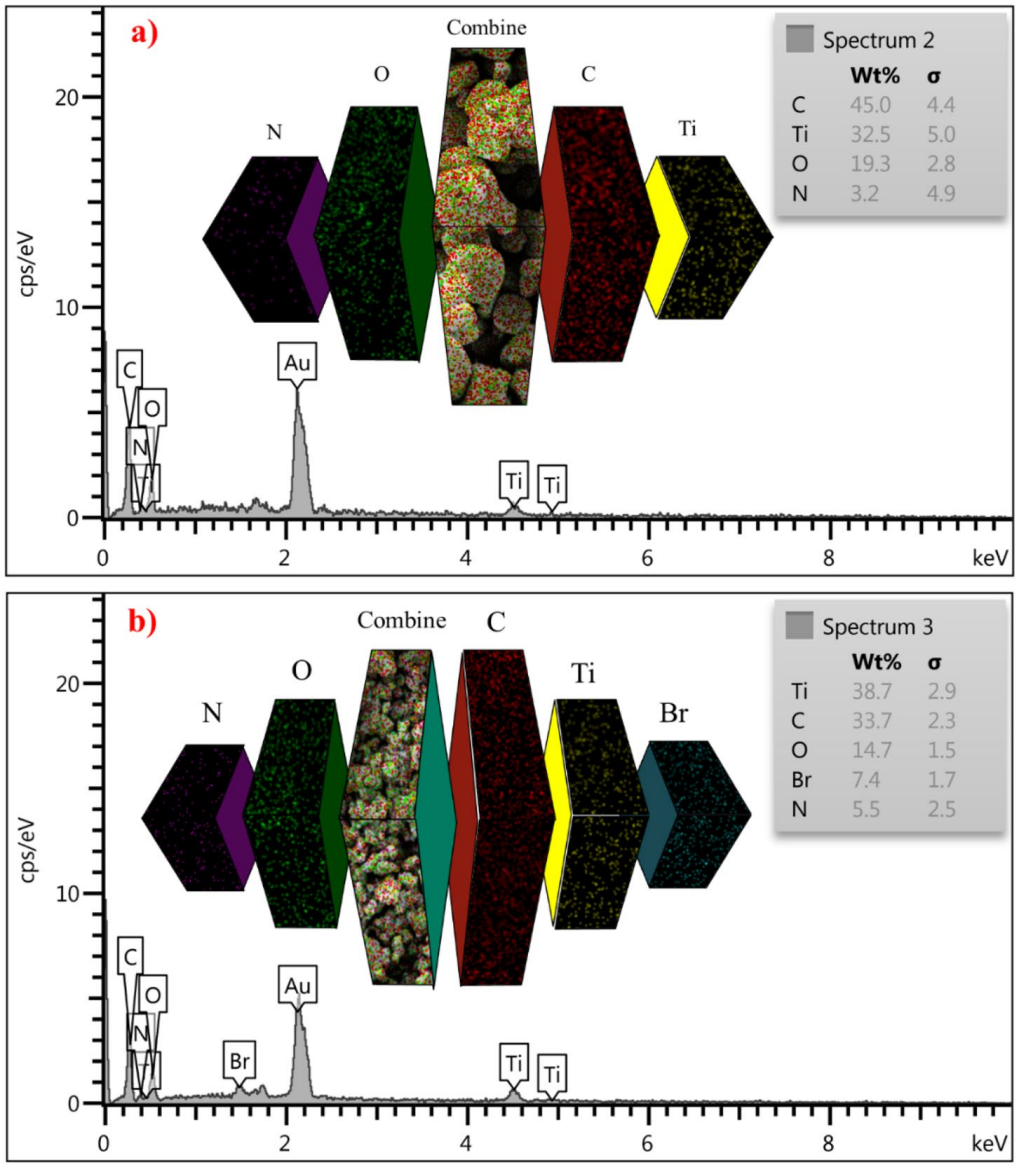
Energy dispersive X-ray (EDX) and elemental mapping analysis of (**a**) MOF(Ti)-NH_2_ and (**b**) Ti-based MOFs functionalized with acetic acid as a porous catalyst.

Energy dispersive X-ray (EDX) technique was used to examine the elements in the catalyst structure. The results are shown in Fig. 7. According to the obtained results, Carbon (C), Nitrogen (N), Oxygen (O), and Titanium (Ti) elements are present in MIL-125(Ti)-NH_2_ structure. In the structure of the final catalyst, in addition to the above elements, the presence of bromine (Br) element has been proven. The results of elemental mapping analysis confirm both the existence of these elements and the uniform distribution of elements on the surface of the catalyst (Fig. 7).

**Figure 8:**
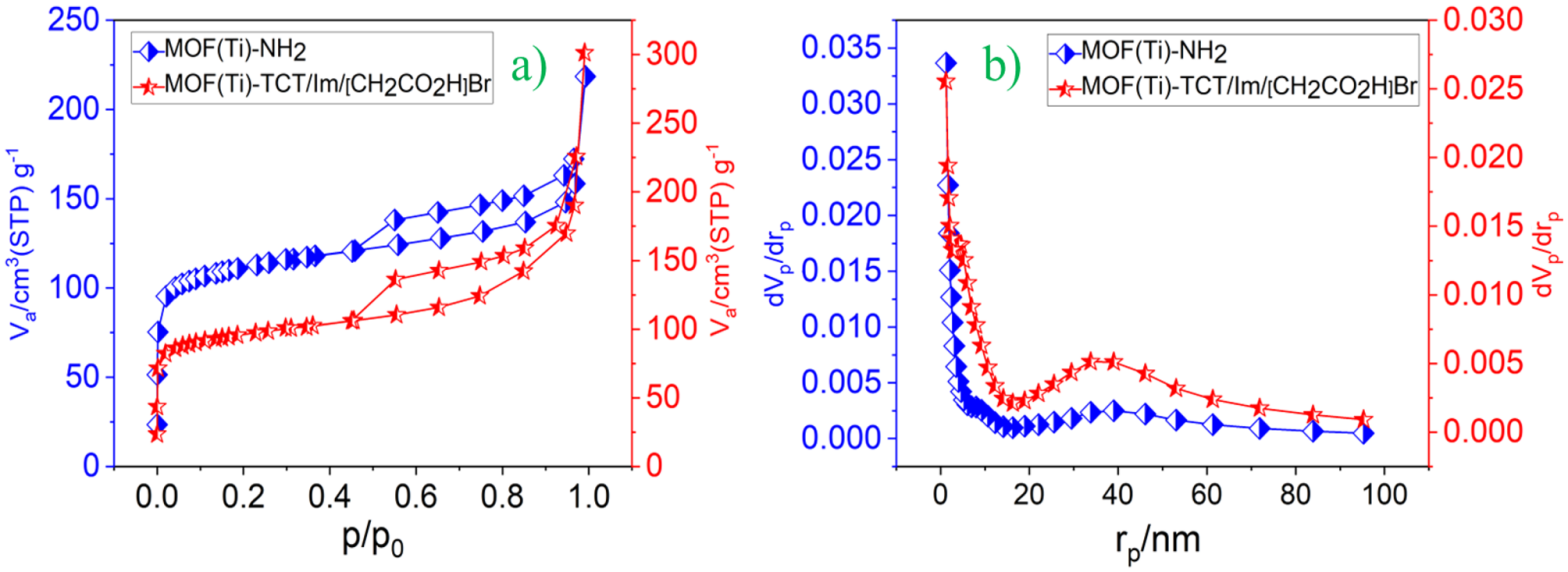
(**a**) N_2_ adsorption/desorption and (**b**) pore size distribution based on BJH analysis of MOF(Ti)-NH_2_ and Ti-based MOFs functionalized with acetic acid as a porous catalyst.

N_2_-adsorption/desorption isotherms of the final catalyst as well as MIL-125(Ti)-NH_2_ were measured and presented in Fig. 8a. Using BET equation, the calculated surface area for MIL-125(Ti)-NH_2_ and the final catalyst, are 420 and 357 m^2^g^−1^, respectively. The obtained total pore volume for MIL-125(Ti)-NH_2_ and the final catalyst are 0.462 and 0.329 cm^3^g^−1^, respectively. The pore size distribution based on the BJH method is shown in Fig. 8b, revealing the presence of micropores and mesopores in both samples. The mean pore diameter for MIL-125(Ti)-NH_2_ and the final catalyst are 5.2 and 3.1 nm, respectively. The high surface area of the catalyst corresponds to the presence of more reactive sites and consequently higher catalytic activity. This feature is well proven by the data on the Ti-based MOFs functionalized with acetic acid as a porous catalyst.

### Catalytic activity

**Figure 9:**
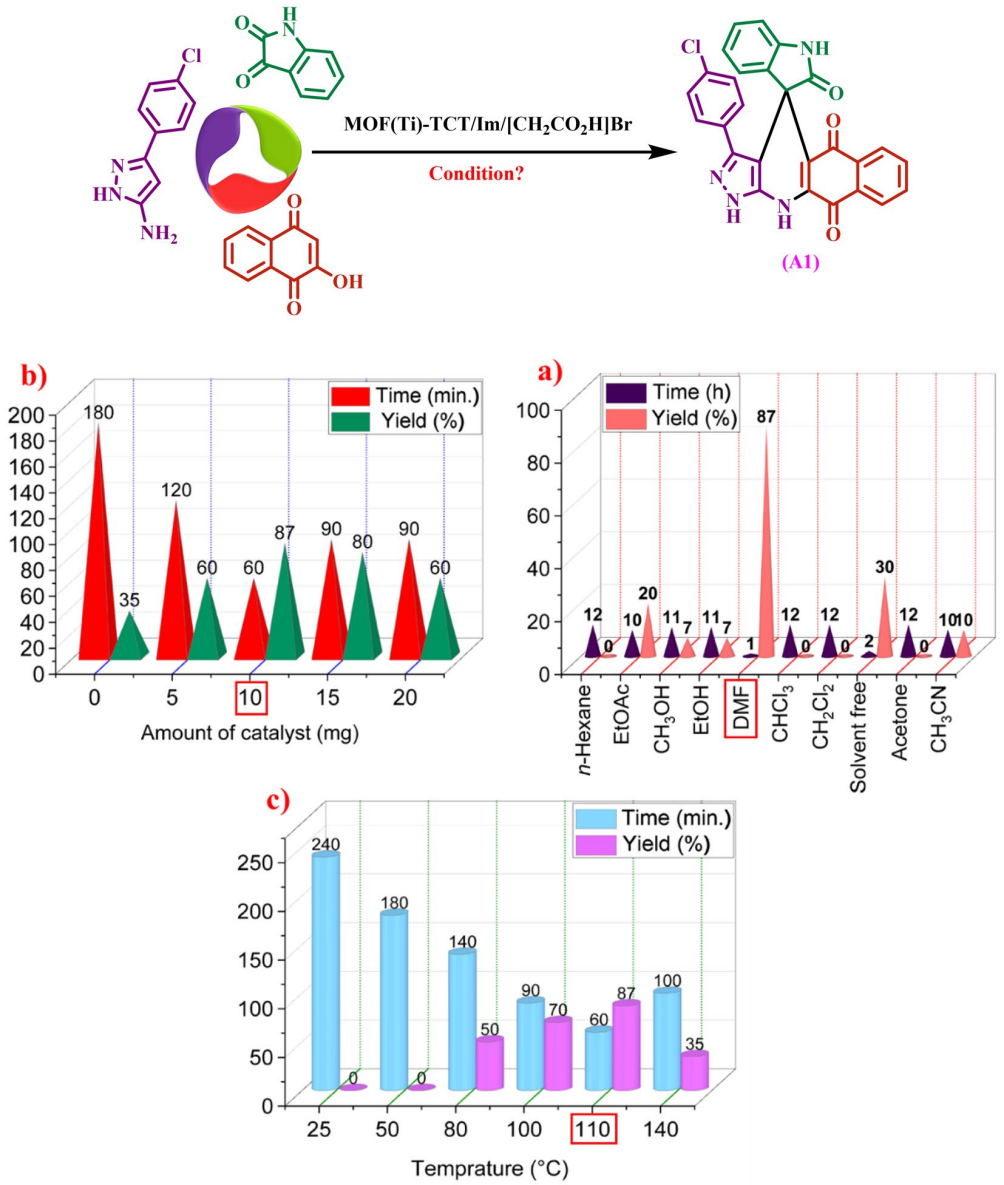
Optimization of some parameter’s reaction Ti-based MOFs functionalized with acetic acid as a porous catalyst: (**a**) solvent (**b**) amount of catalyst and c) temperature.

After the complete identification of the synthesized catalyst, the accuracy of its structure was proven using different techniques. This porous catalyst was used in the preparation of new spiropyrans. To prepare these compounds, the reaction between 3-(4-chlorophenyl)-1*H*-pyrazol-5-amine (1 mmol, 0.193 g), isatin (1 mmol, 0.147 g), and 2-hydroxynaphthalene-1,4-dione (Henna) (1 mmol, 0.174 g) was chosen as a model reaction (compound A1) to obtain the optimal conditions. The model reaction was evaluated using different solvents as well as solvent-free conditions. After optimization of solvents, DMF was selected as the most suitable solvent (Fig. 9a). In another study, the model reaction with different amounts of catalyst (Fig. 9b) and different temperatures (Fig. 9c) was investigated. According to the obtained results, the amount of 10 mg of the catalyst at 110 °C in DMF solvent was identified as the optimal condition.

**Figure 10:**
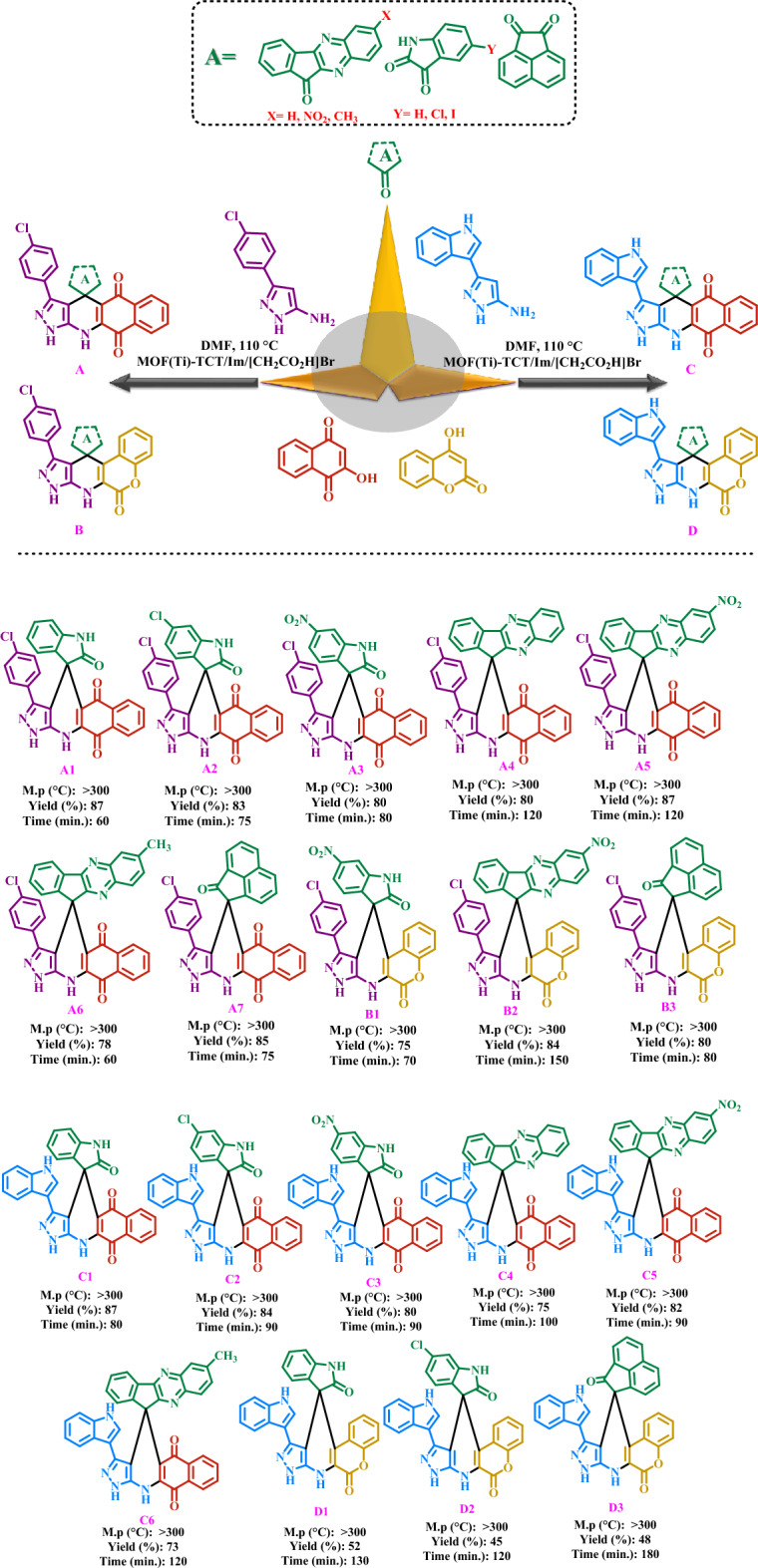
Preparation of new spiropyrans including biological moieties using Ti-based MOFs functionalized with acetic acid as a porous catalyst.

According to the optimal reaction conditions specified in the preparation of target spiropyrans, different ketones of category A, amines, as well as 2-hydroxynaphthalene-1,4-dione (Henna) and 4-hydroxy-2*H*-chromen-2-one were used to synthesize a wide range of spiropyrans. The results are shown in Fig. 10. According to the results of Fig. 10, the products were prepared in relatively short reaction time and high yield. The obtained results reveal the catalytic performance of Ti-based MOFs functionalized with acetic acid as a porous catalyst in the course of synthesis of target spiropyrans.

**Figure 11:**
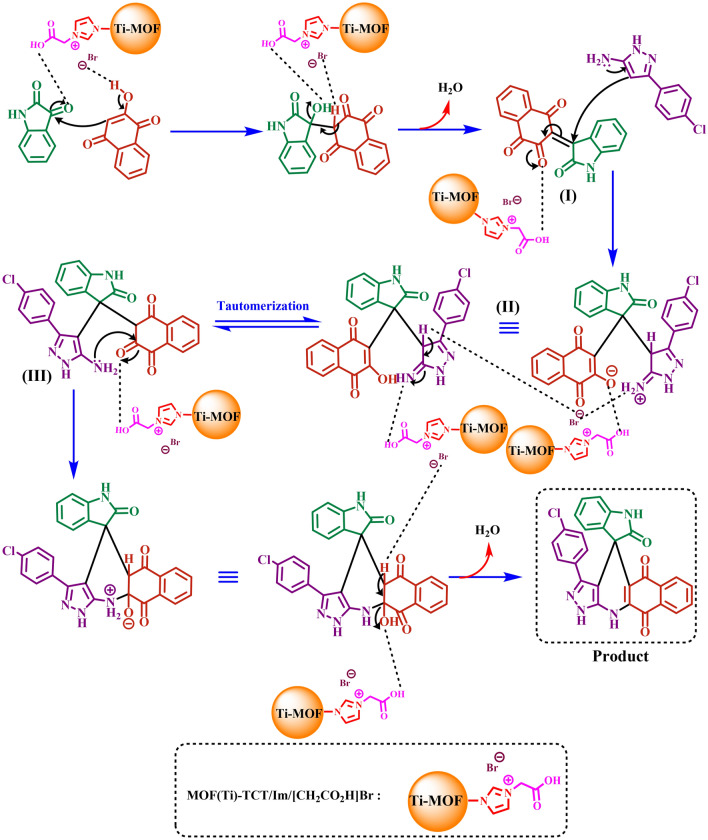
The proposed mechanism for preparation of new spiropyrans including biological moieties using Ti-based MOFs functionalized with acetic acid as a porous catalyst.

A mechanism is proposed for the synthesized product (A1) using Ti-based MOFs functionalized with acetic acid as a porous catalyst (Fig. 11). In the proposed mechanism, at first, isatin is activated by the catalyst, and henna compound reacts with activated isatin. Intermediate (I) is created from the reaction of these two structures and the removal of one H_2_O molecule. Next, 3-(4-chlorophenyl)-1*H*-pyrazol-5-amine is added to intermediate (I), which is a Michael acceptor, and intermediate (II) is produced. Further, intermediate (II) is converted to intermediate (III) through tautomerization. The intermediate (III) is converted to the final product through intramolecular cyclization and the loss of another H_2_O molecule. Other synthesized spiropyran derivatives proceed according to the same mechanism.

**Figure 12:**
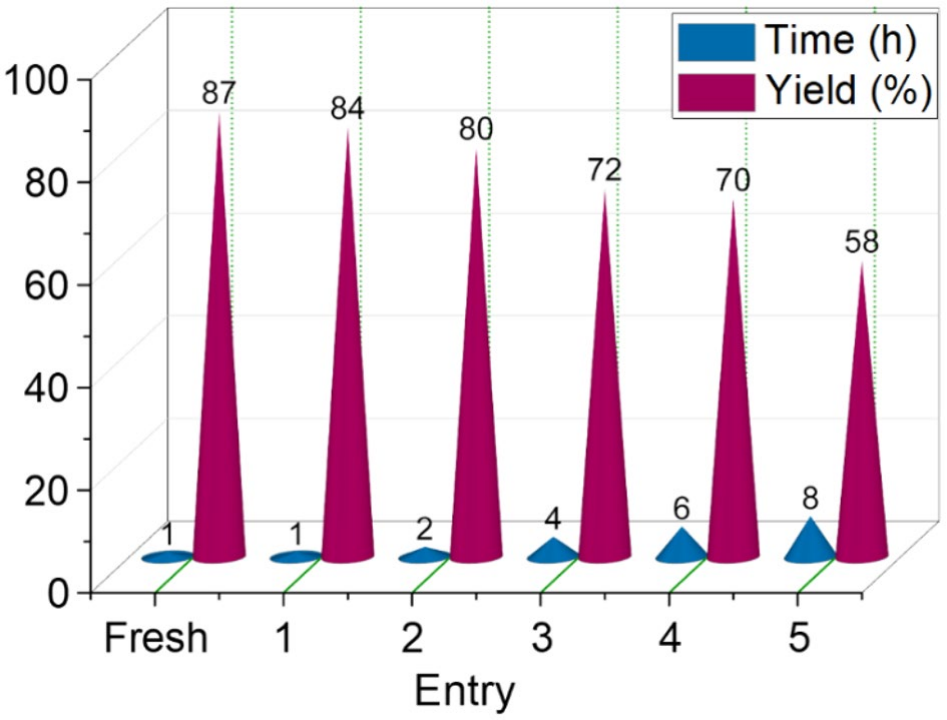
Recyclability of Ti-based MOFs functionalized with acetic acid as a porous catalyst.

The effectiveness and importance of the synthesized catalyst were evaluated from another method. For this purpose, the model reaction was evaluated using other organic and inorganic catalysts reported in the literature. The results are shown in Table 1. The results show that Ti-based MOFs functionalized with acetic acid as a porous catalyst produce the desired product with a higher yield and shorter reaction time. Another significant aspect of the synthesized catalyst is its recyclability. Following the completion of the model reaction and the formation of the target product, the catalyst was separated, washed, and reused for subsequent model reactions. The results illustrating the recyclability of the catalyst are presented in Fig. 12. According to the results obtained from Fig. 12, the prepared catalyst has shown a good efficiency up to 4 times, and the recovery period of 5 efficiency has decreased a bit. According to the obtained results, the above-mentioned catalyst can be recycled up to 4 times. The results obtained from these investigations show the proper performance of MOF(Ti)-TCT/Im/[CH_2_CO_2_H]Br as a porous catalyst that can both increase the yield of the reaction with recycle ability. These two characteristics, increased reaction yield and recyclability, are essential attributes of an efficient catalyst.

**Table 1:** Synthesis of A1 compound as a model reaction in the presence of various catalysts.

Entry	Catalyst	Amount of catalyst	Time (min.)	Yield (%)
1	CQDs-N(CH_2_PO_3_H_2_)_2_^52^	10 mg	120	30
2	CQDs-N(CH_2_PO_3_H_2_)_2_/SBA-15^53^	10 mg	100	50
3	MIL88-B(Fe_2_/Co)-N(CH_2_PO_3_H_2_)_2_^40^	10 mg	140	Trace
4	Ti-MOF-UR^44^	10 mg	120	35
5	*p-*TSA	10 mol%	120	35
6	Piperidine	10 mol%	140	15
7	H_2_SO_4_	10 mol%	200	–
8	NaOH	10 mol%	120	20
9	SSA^82,83^	10 mg	200	–
10	Acetic acid	10 mol%	140	50
11	Citric acid	10 mol%	120	40
12	H_3_PO_4_	10 mol%	180	20
13	NaHSO_4_	10 mol%	180	–
14	NaNH_2_PO_4_	10 mol%	180	–
15	Oxalic acid two-hydrate	10 mol%	180	Trace
16	MOF(Ti)-TCT/Im/[CH_2_CO_2_H]Br (this work)	10 mg	60	87

## Conclusion

In summary, the aim was to develop heterogeneous porous catalysts based on a post-modification strategy. The metal-organic framework based on Ti was chosen to achieve this goal. Next, MIL-125(Ti)-NH_2_ was modified using acidic groups, and acetic acid was created on this porous structure. The reason for choosing MIL-125(Ti)-NH_2_ was the appropriate disk-like morphology and high surface area of this structure, which creates a suitable substrate for its catalytic application. The structure of the target catalyst was approved using various techniques. The catalytic application of MOF(Ti)-TCT/Im/[CH_2_CO_2_H]Br as a porous catalyst in the preparation of new spiropyrans was evaluated and the obtained results showed that the catalytic performance was improved by this method. In the structure of the synthesized spiropyrans, biological components such as indole, henna, coumarin, pyrazole, and isatin were used. The synthesis of the compounds was done using the target catalyst, under mild conditions, short reaction time, and high yield, which is one of the most important features for the design, synthesis, application, and introduction of any task-specific catalyst. Another feature of the synthesized catalyst was its recyclability, which gave it a special feature.

### Supplementary Information

Supplementary Information.

## Data Availability

The datasets used and/or analyzed during the current study are available from the corresponding author on reasonable request.
