# Supplementary material for: Ti-based MOFs with acetic acid pendings as an efficient catalyst in the preparation of new spiropyrans with biological moieties
Source: Sci Rep. 2024 Jun 19;14:14101. doi: 10.1038/s41598-024-62757-x (PMC11189590; doi:10.1038/s41598-024-62757-x)
Supplement: Supplementary file 1 — Supplementary Information. [file 41598_2024_62757_MOESM1_ESM.docx]

**Supporting Information**

**Ti-based MOFs with acetic acid pendings as an efficient catalyst in the preparation of new spiropyrans with biological moieties**

Zahra Torkashvand, ^a^ Hassan Sepehrmansourie, ^a^ Mohammad Ali Zolfigol*^a^ Yanlong Gu^b^

^a^ Department of Organic Chemistry, Faculty of Chemistry and Petroleum Sciences, Bu-Ali Sina University, Hamedan 6517838683, Tel: +988138282807, Fax: +988138380709 Iran.

^b^ School of Chemistry and Chemical Engineering, Huazhong University of Science and Technology, 1037 Luoyu road, Hongshan District, Wuhan 430074.

Table of Contents

[Supplementary Figure 1: FT-IR spectrum of 3-(4-chlorophenyl)-1,11-dihydrospiro[benzo[*g*]pyrazolo[3,4-*b*]quinoline-4,3'-indoline]-2',5,10-trione (A1). 7](#_Toc159684458)

[Supplementary Figure 2: ^1^HNMR spectrum of 3-(4-chlorophenyl)-1,11-dihydrospiro[benzo[*g*]pyrazolo[3,4-*b*]quinoline-4,3'-indoline]-2',5,10-trione (A1). 7](#_Toc159684459)

[Supplementary Figure 3: ^13^CNMR spectrum of 6'-chloro-3-(4-chlorophenyl)-1,11-dihydrospiro[benzo[*g*]pyrazolo[3,4-*b*]quinoline-4,3'-indoline]-2',5,10-trione (A2). 8](#_Toc159684460)

[Supplementary Figure 4: FT-IR spectrum of 6'-chloro-3-(4-chlorophenyl)-1,11-dihydrospiro[benzo[*g*]pyrazolo[3,4-*b*]quinoline-4,3'-indoline]-2',5,10-trione (A2). 8](#_Toc159684461)

[Supplementary Figure 5: ^1^HNMR spectrum of 6'-chloro-3-(4-chlorophenyl)-1,11-dihydrospiro[benzo[*g*]pyrazolo[3,4-*b*]quinoline-4,3'-indoline]-2',5,10-trione (A2). 9](#_Toc159684462)

[Supplementary Figure 6: ^13^CNMR spectrum of 6'-chloro-3-(4-chlorophenyl)-1,11-dihydrospiro[benzo[*g*]pyrazolo[3,4-*b*]quinoline-4,3'-indoline]-2',5,10-trione (A2). 9](#_Toc159684463)

[Supplementary Figure 7: FT-IR spectrum of 3-(4-chlorophenyl)-6'-nitro-1,11-dihydrospiro[benzo[*g*]pyrazolo[3,4-*b*]quinoline-4,3'-indoline]-2',5,10-trione (A3). 10](#_Toc159684464)

[Supplementary Figure 8: ^1^HNMR spectrum of 3-(4-chlorophenyl)-6'-nitro-1,11-dihydrospiro[benzo[*g*]pyrazolo[3,4-*b*]quinoline-4,3'-indoline]-2',5,10-trione (A3). 10](#_Toc159684465)

[Supplementary Figure 9: ^13^CNMR spectrum of 3-(4-chlorophenyl)-6'-nitro-1,11-dihydrospiro[benzo[*g*]pyrazolo[3,4-*b*]quinoline-4,3'-indoline]-2',5,10-trione (A3). 11](#_Toc159684466)

[Supplementary Figure 10: FT-IR spectrum of 3-(4-chlorophenyl)-1,11-dihydrospiro[benzo[*g*]pyrazolo[3,4-*b*]quinoline-4,11'-indeno[1,2-*b*]quinoxaline]-5,10-dione (A4). 11](#_Toc159684467)

[Supplementary Figure 11: ^1^HNMR spectrum of 3-(4-chlorophenyl)-1,11-dihydrospiro[benzo[*g*]pyrazolo[3,4-*b*]quinoline-4,11'-indeno[1,2-*b*]quinoxaline]-5,10-dione (A4). 12](#_Toc159684468)

[Supplementary Figure 12: ^13^CNMR spectrum of 3-(4-chlorophenyl)-1,11-dihydrospiro[benzo[*g*]pyrazolo[3,4-*b*]quinoline-4,11'-indeno[1,2-*b*]quinoxaline]-5,10-dione (A4). 12](#_Toc159684469)

[Supplementary Figure 13: FT-IR spectrum of 3-(4-chlorophenyl)-7'-nitro-1,11-dihydrospiro[benzo[*g*]pyrazolo[3,4-*b*]quinoline-4,11'-indeno[1,2-*b*]quinoxaline]-5,10-dione (A5). 13](#_Toc159684470)

[Supplementary Figure 14: ^1^HNMR spectrum of 3-(4-chlorophenyl)-7'-nitro-1,11-dihydrospiro[benzo[*g*]pyrazolo[3,4-*b*]quinoline-4,11'-indeno[1,2-*b*]quinoxaline]-5,10-dione (A5). 13](#_Toc159684471)

[Supplementary Figure 15: FT-IR spectrum of 3-(4-chlorophenyl)-7'-methyl-1,11-dihydrospiro[benzo[*g*]pyrazolo[3,4-*b*]quinoline-4,11'-indeno[1,2-*b*]quinoxaline]-5,10-dione (A6). 14](#_Toc159684472)

[Supplementary Figure 16: ^1^HNMR spectrum of 3-(4-chlorophenyl)-7'-methyl-1,11-dihydrospiro[benzo[*g*]pyrazolo[3,4-*b*]quinoline-4,11'-indeno[1,2-*b*]quinoxaline]-5,10-dione (A6). 14](#_Toc159684473)

[Supplementary Figure 17: FT-IR spectrum of 3-(4-chlorophenyl)-1',11'-dihydro-2*H*-spiro[acenaphthylene-1,4'-benzo[*g*]pyrazolo[3,4-*b*]quinoline]-2,5',10'-trione (A7). 15](#_Toc159684474)

[Supplementary Figure 18: ^1^HNMR spectrum of 3-(4-chlorophenyl)-1',11'-dihydro-2*H*-spiro[acenaphthylene-1,4'-benzo[*g*]pyrazolo[3,4-*b*]quinoline]-2,5',10'-trione (A7). 15](#_Toc159684475)

[Supplementary Figure 19: ^13^CNMR spectrum of 3-(4-chlorophenyl)-1',11'-dihydro-2*H*-spiro[acenaphthylene-1,4'-benzo[*g*]pyrazolo[3,4-*b*]quinoline]-2,5',10'-trione (A7). 16](#_Toc159684476)

[Supplementary Figure 20: FT-IR spectrum of 10-(4-chlorophenyl)-6'-nitro-7,8-dihydro-6*H*-spiro[chromeno[3,4-*b*]pyrazolo[4,3-*e*]pyridine-11,3'-indoline]-2',6-dione (B1). 16](#_Toc159684477)

[Supplementary Figure 21: ^1^HNMR spectrum of 10-(4-chlorophenyl)-6'-nitro-7,8-dihydro-6*H*-spiro[chromeno[3,4-*b*]pyrazolo[4,3-*e*]pyridine-11,3'-indoline]-2',6-dione (B1). 17](#_Toc159684478)

[Supplementary Figure 22: ^13^CNMR spectrum of 10-(4-chlorophenyl)-6'-nitro-7,8-dihydro-6*H*-spiro[chromeno[3,4-*b*]pyrazolo[4,3-*e*]pyridine-11,3'-indoline]-2',6-dione (B1). 17](#_Toc159684479)

[Supplementary Figure 23: FT-IR spectrum of 10-(4-chlorophenyl)-7'-nitro-7,8-dihydro-6*H*-spiro[chromeno[3,4-*b*]pyrazolo[4,3-*e*]pyridine-11,11'-indeno[1,2-b]quinoxalin]-6-one (B2). 18](#_Toc159684480)

[Supplementary Figure 24: ^1^HNMR spectrum of 10-(4-chlorophenyl)-7'-nitro-7,8-dihydro-6*H*-spiro[chromeno[3,4-*b*]pyrazolo[4,3-*e*]pyridine-11,11'-indeno[1,2-b]quinoxalin]-6-one (B2). 18](#_Toc159684481)

[Supplementary Figure 25: ^13^CNMR spectrum of 10-(4-chlorophenyl)-7'-nitro-7,8-dihydro-6*H*-spiro[chromeno[3,4-*b*]pyrazolo[4,3-*e*]pyridine-11,11'-indeno[1,2-*b*]quinoxalin]-6-one (B2). 19](#_Toc159684482)

[Supplementary Figure 26: FT-IR spectrum of 10-(4-chlorophenyl)-7',8'-dihydro-2*H*,6'*H*-spiro[acenaphthylene-1,11'-chromeno[3,4-*b*]pyrazolo[4,3-*e*]pyridine]-2,6'-dione (B3). 19](#_Toc159684483)

[Supplementary Figure 27: ^1^HNMR spectrum of 10-(4-chlorophenyl)-7',8'-dihydro-2*H*,6'*H*-spiro[acenaphthylene-1,11'-chromeno[3,4-*b*]pyrazolo[4,3-*e*]pyridine]-2,6'-dione (B3). 20](#_Toc159684484)

[Supplementary Figure 28: ^13^CNMR spectrum of 10-(4-chlorophenyl)-7',8'-dihydro-2*H*,6'*H*-spiro[acenaphthylene-1,11'-chromeno[3,4-*b*]pyrazolo[4,3-*e*]pyridine]-2,6'-dione (B3). 20](#_Toc159684485)

[Supplementary Figure 29: FT-IR spectrum of 3-(1*H*-indol-3-yl)-1,11-dihydrospiro[benzo[*g*]pyrazolo[3,4-*b*]quinoline-4,3'-indoline]-2',5,10-trione (C1). 21](#_Toc159684486)

[Supplementary Figure 30: ^1^HNMR spectrum of 3-(1*H*-indol-3-yl)-1,11-dihydrospiro[benzo[*g*]pyrazolo[3,4-*b*]quinoline-4,3'-indoline]-2',5,10-trione (C1). 21](#_Toc159684487)

[Supplementary Figure 31: ^13^CNMR spectrum of 3-(1*H*-indol-3-yl)-1,11-dihydrospiro[benzo[*g*]pyrazolo[3,4-*b*]quinoline-4,3'-indoline]-2',5,10-trione (C1). 22](#_Toc159684488)

[Supplementary Figure 32: FT-IR spectrum of 6'-chloro-3-(1*H*-indol-3-yl)-1,11-dihydrospiro[benzo[*g*]pyrazolo[3,4-*b*]quinoline-4,3'-indoline]-2',5,10-trioe (C2). 22](#_Toc159684489)

[Supplementary Figure 33: ^1^HNMR spectrum of 6'-chloro-3-(1*H*-indol-3-yl)-1,11-dihydrospiro[benzo[*g*]pyrazolo[3,4-*b*]quinoline-4,3'-indoline]-2',5,10-trioe (C2). 23](#_Toc159684490)

[Supplementary Figure 34: ^13^CNMR spectrum of 6'-chloro-3-(1*H*-indol-3-yl)-1,11-dihydrospiro[benzo[*g*]pyrazolo[3,4-*b*]quinoline-4,3'-indoline]-2',5,10-trioe (C2). 23](#_Toc159684491)

[Supplementary Figure 35: FT-IR spectrum of 3-(1*H*-indol-3-yl)-6'-nitro-1,11-dihydrospiro[benzo[*g*]pyrazolo[3,4-*b*]quinoline-4,3'-indoline]-2',5,10-trione (C3). 24](#_Toc159684492)

[Supplementary Figure 36: ^1^HNMR spectrum of 3-(1*H*-indol-3-yl)-6'-nitro-1,11-dihydrospiro[benzo[*g*]pyrazolo[3,4-*b*]quinoline-4,3'-indoline]-2',5,10-trione (C3). 24](#_Toc159684493)

[Supplementary Figure 37: ^13^CNMR spectrum of 3-(1*H*-indol-3-yl)-6'-nitro-1,11-dihydrospiro[benzo[*g*]pyrazolo[3,4-*b*]quinoline-4,3'-indoline]-2',5,10-trione (C3). 25](#_Toc159684494)

[Supplementary Figure 38: FT-IR spectrum of 3-(1*H*-indol-3-yl)-1,11-dihydrospiro[benzo[*g*]pyrazolo[3,4-*b*]quinoline-4,11'-indeno[1,2-*b*]quinoxaline]-5,10-dione--methane (C4). 25](#_Toc159684495)

[Supplementary Figure 39: ^1^HNMR spectrum of 3-(1*H*-indol-3-yl)-1,11-dihydrospiro[benzo[*g*]pyrazolo[3,4-*b*]quinoline-4,11'-indeno[1,2-*b*]quinoxaline]-5,10-dione--methane (C4). 26](#_Toc159684496)

[Supplementary Figure 40: ^13^CNMR spectrum of 3-(1*H*-indol-3-yl)-1,11-dihydrospiro[benzo[*g*]pyrazolo[3,4-*b*]quinoline-4,11'-indeno[1,2-*b*]quinoxaline]-5,10-dione--methane (C4). 26](#_Toc159684497)

[Supplementary Figure 41: FT-IR spectrum of 3-(1*H*-indol-3-yl)-7'-nitro-1,11-dihydrospiro[benzo[*g*]pyrazolo[3,4-*b*]quinoline-4,11'-indeno[1,2-*b*]quinoxaline]-5,10-dione--methane (C5). 27](#_Toc159684498)

[Supplementary Figure 42: ^1^HNMR spectrum of 3-(1*H*-indol-3-yl)-7'-nitro-1,11-dihydrospiro[benzo[*g*]pyrazolo[3,4-*b*]quinoline-4,11'-indeno[1,2-*b*]quinoxaline]-5,10-dione--methane (C5). 27](#_Toc159684499)

[Supplementary Figure 43: ^13^CNMR spectrum of 3-(1*H*-indol-3-yl)-7'-nitro-1,11-dihydrospiro[benzo[*g*]pyrazolo[3,4-*b*]quinoline-4,11'-indeno[1,2-*b*]quinoxaline]-5,10-dione—methane (C5). 28](#_Toc159684500)

[Supplementary Figure 44: FT-IR spectrum of 3-(1*H*-indol-3-yl)-7'-methyl-1,11-dihydrospiro[benzo[*g*]pyrazolo[3,4-*b*]quinoline-4,11'-indeno[1,2-*b*]quinoxaline]-5,10-dione--methane (C6). 28](#_Toc159684501)

[Supplementary Figure 45: ^1^HNMR spectrum of 3-(1*H*-indol-3-yl)-7'-methyl-1,11-dihydrospiro[benzo[*g*]pyrazolo[3,4-*b*]quinoline-4,11'-indeno[1,2-*b*]quinoxaline]-5,10-dione--methane (C6). 29](#_Toc159684502)

[Supplementary Figure 46: ^13^CNMR spectrum of 3-(1*H*-indol-3-yl)-7'-methyl-1,11-dihydrospiro[benzo[*g*]pyrazolo[3,4-*b*]quinoline-4,11'-indeno[1,2-*b*]quinoxaline]-5,10-dione--methane (C6). 29](#_Toc159684503)

[Supplementary Figure 47: FT-IR spectrum of 10-(1*H*-indol-3-yl)-7,8-dihydro-6*H*-spiro[chromeno[3,4-*b*]pyrazolo[4,3-*e*]pyridine-11,3'-indoline]-2',6-dione (D1). 30](#_Toc159684504)

[Supplementary Figure 48: ^1^HNMR spectrum of 10-(1*H*-indol-3-yl)-7,8-dihydro-6*H*-spiro[chromeno[3,4-*b*]pyrazolo[4,3-*e*]pyridine-11,3'-indoline]-2',6-dione (D1). 30](#_Toc159684505)

[Supplementary Figure 49: ^13^CNMR spectrum of 10-(1*H*-indol-3-yl)-7,8-dihydro-6*H*-spiro[chromeno[3,4-*b*]pyrazolo[4,3-*e*]pyridine-11,3'-indoline]-2',6-dione (D1). 31](#_Toc159684506)

[Supplementary Figure 50: FT-IR spectrum of 6'-chloro-10-(1*H*-indol-3-yl)-7,8-dihydro-6*H*-spiro[chromeno[3,4-*b*]pyrazolo[4,3-*e*]pyridine-11,3'-indoline]-2',6-dione (D2). 31](#_Toc159684507)

[Supplementary Figure 51: ^1^HNMR spectrum of 6'-chloro-10-(1*H*-indol-3-yl)-7,8-dihydro-6*H*-spiro[chromeno[3,4-*b*]pyrazolo[4,3-*e*]pyridine-11,3'-indoline]-2',6-dione (D2). 32](#_Toc159684508)

[Supplementary Figure 52: ^13^CNMR spectrum of 6'-chloro-10-(1*H*-indol-3-yl)-7,8-dihydro-6*H*-spiro[chromeno[3,4-*b*]pyrazolo[4,3-*e*]pyridine-11,3'-indoline]-2',6-dione (D2). 32](#_Toc159684509)

[Supplementary Figure 53: FT-IR spectrum of 10'-(1*H*-indol-3-yl)-7',8'-dihydro-2*H*,6'*H*-spiro[acenaphthylene-1,11'-chromeno[3,4-*b*]pyrazolo[4,3-*e*]pyridine]-2,6'-dione (D3). 33](#_Toc159684510)

[Supplementary Figure 54: ^1^HNMR spectrum of 10'-(1*H*-indol-3-yl)-7',8'-dihydro-2*H*,6'*H*-spiro[acenaphthylene-1,11'-chromeno[3,4-*b*]pyrazolo[4,3-*e*]pyridine]-2,6'-dione (D3). 33](#_Toc159684511)

[Supplementary Figure 55: ^13^CNMR spectrum of 10'-(1*H*-indol-3-yl)-7',8'-dihydro-2*H*,6'*H*-spiro[acenaphthylene-1,11'-chromeno[3,4-*b*]pyrazolo[4,3-*e*]pyridine]-2,6'-dione (D3). 34](#_Toc159684512)

**Spectral data of spiropyrans**

**3-(4-Chlorophenyl)-1,11-dihydrospiro[benzo[*g*]pyrazolo[3,4-*b*]quinoline-4,3'-indoline]-2',5,10-trione (A1).**

Crimson solid, M.p: >300 ˚C; (Ethyl acetate: *n*-Hexane 4:6), FT-IR (KBr, cm^-1^): 3398, 3365, 1701, 1677, 1605, 1538, 1349, 1291, 726. ^1^H NMR (400 MHz, DMSO-d_6_) δ_ppm_ 12.72 (s, 1H), 10.88 (s, 1H), 8.10 (d, *J* = 32.1 Hz, 2H), 7.85 (s, 1H), 7.79 – 7.71 (m, 2H), 7.69 – 7.55 (m, 4H), 7.40 (s, 1H), 6.72 (s, 2H), 6.12 (s, 2H). ^13^C NMR (101 MHz, DMSO-d_6_) δ_ppm_ 180.4, 180.3, 179.5, 145.9, 142.5, 142.2, 139.0, 135.5, 133.9, 133.2, 132.6, 130.8, 130.4, 128.3, 128.3, 126.3, 126.1, 124.0, 122.0, 112.2, 109.6, 102.3, 49.8.

**6'-Chloro-3-(4-chlorophenyl)-1,11-dihydrospiro[benzo[*g*]pyrazolo[3,4-*b*]quinoline-4,3'-indoline]-2',5,10-trione (A2).**

Crimson solid, M.p: >300 ˚C; (Ethyl acetate: n-Hexane 4:6), FT-IR (KBr, cm-1):3445, 3354, 3246, 1719, 1506, 1677, 1350, 727. ^1^H NMR (400 MHz, DMSO-d_6_) δ_ppm_ 12.83 (s, 1H), 10.82 (s, 1H), 10.22 (s, 1H), 7.80-7.76 (m, 2H), 7.57 (dd, J = 33.1, 5.2 Hz, 2H), 7.31 (d, J = 8.5 Hz, 2H), 7.14 (s, 2H), 6.71 (d, J = 8.3 Hz, 2H), 6.58 (d, J = 8.9 Hz, 1H). ^13^C NMR (101 MHz, DMSO-d_6_) δ_ppm_ 179.9, 179.8, 178.9, 145.3, 142.3, 140.5, 140.0, 138.2, 135.0, 133.7, 132.8, 132.1, 130.3, 130.1, 129.9, 127.9, 127.6, 127.3, 126.1, 125.9, 125.63, 125.4, 123.6, 111.2, 110.4, 101.4, 49.5.

**3-(4-Chlorophenyl)-6'-nitro-1,11-dihydrospiro[benzo[*g*]pyrazolo[3,4-*b*]quinoline-4,3'-indoline]-2',5,10-trione (A3).**

Crimson solid, M.p: >300 ˚C; (Ethyl acetate: *n*-Hexane 4:6), FT-IR (KBr, cm^-1^): 3406, 3353, 1719, 1539, 1477, 1351, 1095, 735. ^1^H NMR (400 MHz, DMSO-d_6_) δ_ppm_ 12.81 (s, 1H), 10.80 (s, 1H), 10.20 (s, 1H), 8.05 (d, *J* = 7.5 Hz, 1H), 7.85 – 7.73 (m, 3H), 7.31 (d, *J* = 8.5 Hz, 2H), 7.13 (d, *J* = 2.1 Hz, 2H), 6.71 (d, *J* = 8.5 Hz, 2H), 6.57 (d, *J* = 8.7 Hz, 1H). ^13^C NMR (101 MHz, DMSO-d_6_) δ_ppm_ 179.8, 179.8, 178.8, 145.3, 142.3, 140.5, 140.1, 138.1, 134.9, 133.6, 132.7, 132.1, 130.4, 130.0, 127.9, 127.5, 127.4, 125.8, 125.6, 125.3, 123.7, 111.2, 110.3, 101.4, 49.5.

**3-(4-Chlorophenyl)-1,11-dihydrospiro[benzo[*g*]pyrazolo[3,4-*b*]quinoline-4,11'-indeno[1,2-*b*]quinoxaline]-5,10-dione (A4).**

Crimson solid, M.p: >300 ˚C; (Ethyl acetate: *n*-Hexane 4:6), FT-IR (KBr, cm^-1^): 3406, 1687, 1669, 1610, 1338, 1211, 999, 756. ^1^H NMR (400 MHz, DMSO-d_6_) δ_ppm_ 12.72 (s, 1H), 10.97 (s, 1H), 8.09 – 8.03 (m, 2H), 7.92 – 7.87 (m, 2H), 7.79 – 7.69 (m, 4H), 7.54 – 7.45 (m, 4H), 6.81 (d, *J* = 8.3 Hz, 2H), 6.01 (d, *J* = 8.5 Hz, 2H). ^13^C NMR (101 MHz, DMSO-d_6_) δ_ppm_ 179.2, 178.0, 167.3, 160.3, 152.4, 142.9, 141.4, 139.9, 136.7, 133.4, 131.7, 130.8, 130.4, 129.8, 128.7, 128.4, 128.1, 125.9, 125.7, 124.5, 124.3, 124.0, 123.8, 122.4, 119.7, 118.0, 117.2, 113.9, 111.2, 110.8, 101.7, 57.4.

**3-(4-Chlorophenyl)-7'-nitro-1,11-dihydrospiro[benzo[*g*]pyrazolo[3,4-*b*]quinoline-4,11'-indeno[1,2-*b*]quinoxaline]-5,10-dione (A5).**

Brown solid, M.p: >300 ˚C; (Ethyl acetate: *n*-Hexane 4:6), FT-IR (KBr, cm^-1^): 3429, 3320, 1685, 1669, 1612, 1593, 1537, 1340, 1187, 718. ^1^H NMR (400 MHz, DMSO-d_6_) δ_ppm_ 12.80 (s, 1H), 11.11 (s, 1H), 8.97 (s, 1H), 8.73 (d, *J* = 18.0 Hz, 1H), 8.60 (t, *J* = 9.1 Hz, 1H), 8.51 – 8.46 (m, 1H), 8.27 (d, *J* = 8.5 Hz, 1H), 8.18 (d, *J* = 7.1 Hz, 1H), 7.97 – 7.93 (m, 2H), 7.82 (t, *J* = 7.0 Hz, 1H), 7.60 – 7.58 (m, 1H), 7.50 (s, 1H), 6.82 (s, 2H), 6.05 (d, *J* = 8.3 Hz, 2H).

**3-(4-Chlorophenyl)-7'-methyl-1,11-dihydrospiro[benzo[*g*]pyrazolo[3,4-*b*]quinoline-4,11'-indeno[1,2-*b*]quinoxaline]-5,10-dione (A6).**

Crimson solid, M.p: >300 ˚C; (Ethyl acetate: *n*-Hexane 4:6), FT-IR (KBr, cm^-1^): 3411, 3245, 1687, 1609, 1510 ,1298, 999, 725. ^1^H NMR (400 MHz, DMSO-d_6_) δ_ppm_ 12.70 (s, 1H), 10.94 (s, 1H), 8.05 (d, *J* = 7.2 Hz, 1H), 7.91 – 7.83 (m, 2H), 7.74 – 7.66 (m, 3H), 7.55 – 7.43 (m, 5H), 6.82 (d, *J* = 8.0 Hz, 2H), 6.00 (dd, *J* = 8.5, 2.6 Hz, 2H), 2.55 (s, 3H).

**3-(4-Chlorophenyl)-1',11'-dihydro-2*H*-spiro[acenaphthylene-1,4'-benzo[*g*]pyrazolo[3,4-*b*]quinoline]-2,5',10'-trione (A7).**

Dark violet solid, M.p: >300 ˚C; (Ethyl acetate: *n*-Hexane 4:6), FT-IR (KBr, cm^-1^): 3406, 1721, 1674, 1574, 1537, 1344, 1002, 781. ^1^H NMR (400 MHz, DMSO-d_6_) δ 12.72 (s, 1H), 10.88 (s, 1H), 8.10 (d, *J* = 32.1 Hz, 2H), 7.85 (s, 1H), 7.79 – 7.71 (m, 2H), 7.69 – 7.55 (m, 4H), 7.40 (s, 1H), 6.72 (s, 2H), 6.12 (s, 2H). ^13^C NMR (101 MHz, DMSO-d_6_) δ 180.4, 180.2, 168.1, 141.6, 141.0, 138.6, 135.9, 135.4, 133.7, 133.3, 132.6, 132.4, 130.4, 130.2, 129.7, 129.3, 129.0, 128.8, 127.9, 127.7, 127.2, 126.4, 126.0, 125.6, 121.6, 113.6, 104.3, 50.1.

**10-(4-Chlorophenyl)-6'-nitro-7,8-dihydro-6*H*-spiro[chromeno[3,4-*b*]pyrazolo[4,3-*e*]pyridine-11,3'-indoline]-2',6-dione (B1).**

Light cream solid, M.p: >300 ˚C; (Ethyl acetate: *n*-Hexane 4:6), FT-IR (KBr, cm^-1^): 3368, 3259, 1733, 1714, 1519, 1342, 1017, 755. ^1^H NMR (400 MHz, DMSO-d_6_) δ_ppm_ 12.88 (s, 1H), 11.61 (s, 1H), 10.78 (s, 1H), 8.51 (d, 1H), 8.21 (d, *J* = 8.8 Hz, 1H), 8.07 (d, *J* = 8.6 Hz, 1H), 7.85 (t, *J* = 7.9 Hz, 1H), 7.59 (t, *J* = 7.7 Hz, 1H), 7.54 (d, 1H), 7.50 – 7.42 (m, 1H), 7.30 (d, *J* = 8.0 Hz, 2H), 6.75 (d, *J* = 8.2 Hz, 2H). ^13^C NMR (101 MHz, DMSO-d_6_) δ_ppm_ 179.5, 177.3, 159.6, 158.3, 155.2, 151.9, 150.4, 148.4, 142.3, 142.2, 134.1, 132.5, 130.4, 128.0, 126.5, 125.3, 125.2, 123.7, 120.6, 116.7, 116.5, 112.4, 108.9, 102.1, 52.3.

**10-(4-Chlorophenyl)-7'-nitro-7,8-dihydro-6*H*-spiro[chromeno[3,4-*b*]pyrazolo[4,3-*e*]pyridine-11,11'-indeno[1,2-*b*]quinoxalin]-6-one (B2).**

Light cream solid, M.p: >300 ˚C; (Ethyl acetate: *n*-Hexane 4:6), FT-IR (KBr, cm^-1^): 3422, 3221, 1701, 1670, 1617, 1591, 1472, 1346, 1092, 755. ^1^H NMR (400 MHz, DMSO-d_6_) δ_ppm_ 12.69 (s, 1H), 11.08 (s, 1H), 8.52 (d, *J* = 8.3 Hz, 1H), 8.12 (d, *J* = 7.9 Hz, 1H), 7.87 (d, *J* = 8.2 Hz, 1H), 7.63 (dd, *J* = 21.2, 13.2 Hz, 5H), 7.47 (s, 1H), 7.37 (d, *J* = 7.4 Hz, 2H), 6.71 (d, *J* = 8.2 Hz, 2H), 6.11 (d, *J* = 8.2 Hz, 2H). ^13^C NMR (101 MHz, DMSO-d_6_) δ_ppm_ 172.5, 169.3, 158.6, 157.6, 154.6, 152.7, 146.3, 144.4, 144.4, 139.4, 134.9, 133.6, 130.0, 130.0, 129.8, 129.7, 129.7, 128.6, 127.5, 125.0, 124.5, 124.5, 122.0, 121.7, 116.8, 96.9, 46.4.

**10-(4-Chlorophenyl)-7',8'-dihydro-2*H*,6'*H*-spiro[acenaphthylene-1,11'-chromeno[3,4-*b*]pyrazolo[4,3-*e*]pyridine]-2,6'-dione (B3).**

Light cream solid, M.p: >300 ˚C; (Ethyl acetate: *n*-Hexane 4:6), FT-IR (KBr, cm^-1^): 3427, 3256, 1715, 1614, 1590, 1544, 995, 781. ^1^H NMR (400 MHz, DMSO-d_6_) δ_ppm_ 12.69 (s, 1H), 11.08 (s, 1H), 8.52 (d, *J* = 8.3 Hz, 1H), 8.12 (d, *J* = 7.9 Hz, 1H), 7.87 (d, *J* = 8.2 Hz, 1H), 7.73 – 7.54 (m, 4H), 7.47 (s, 1H), 7.37 (d, *J* = 7.4 Hz, 2H), 6.71 (d, *J* = 8.2 Hz, 2H), 6.11 (d, *J* = 8.2 Hz, 2H). ^13^C NMR (101 MHz, DMSO-d_6_) δ_ppm_ 204.5, 160.6, 160.6, 152.5, 146.9, 146.7, 146.3, 140.9, 138.4, 133.5, 132.8, 131.2, 130.2, 130.0, 129.3, 128.4, 127.5, 127.3, 124.6, 124.5, 123.8, 121.6, 120.4, 117.2, 114.0, 114.0, 103.6, 103.6, 96.4, 59.1.

**3-(1*H*-Indol-3-yl)-1,11-dihydrospiro[benzo[*g*]pyrazolo[3,4-*b*]quinoline-4,3'-indoline]-2',5,10-trione (C1).**

Dark violet solid, M.p: >300 ˚C; (Ethyl acetate: *n*-Hexane 4:6), FT-IR (KBr, cm^-1^): 3437, 3347, 1720, 1667, 15621352, 998, 743. ^1^H NMR (400 MHz, DMSO-d_6_) δ_ppm_ 12.53 (s, 1H), 11.23 (s, 1H), 10.68 (s, 1H), 10.06 (s, 1H), 8.05 (d, *J* = 7.1 Hz, 1H), 7.82 – 7.72 (m, 3H), 7.34 (d, *J* = 8.1 Hz, 1H), 7.26 (d, *J* = 7.9 Hz, 1H), 7.17 – 7.08 (m, 3H), 6.98 (t, *J* = 7.5 Hz, 1H), 6.88 (t, *J* = 7.6 Hz, 1H), 6.63 (d, *J* = 7.7 Hz, 1H), 5.83 (s, 1H). ^13^C NMR (101 MHz, DMSO-d_6_) δ_ppm_ 180.6, 180.2, 179.5, 142.7, 142.4, 139.7, 135.9, 135.5, 133.1, 132.8, 130.3, 128.1, 126.8, 126.3, 126.1, 125.0, 124.1, 121.9, 121.8, 119.8, 119.4, 112.7, 111.8, 109.4, 102.9, 101.8, 50.0.

**6'-Chloro-3-(1*H*-indol-3-yl)-1,11-dihydrospiro[benzo[*g*]pyrazolo[3,4-*b*]quinoline-4,3'-indoline]-2',5,10-trioe (C2).**

Dark violet solid, M.p: >300 ˚C; (Ethyl acetate: *n*-Hexane 4:6), FT-IR (KBr, cm^-1^): 3437, 3321, 1726, 1667, 1597, 1296, 960, 736. ^1^H NMR (400 MHz, DMSO-d_6_) δ_ppm_ 12.58 (s, 1H), 11.04 (s, 1H), 10.91 (s, 1H), 8.67 (s, 1H), 8.35 (d, 1H), 8.07 (d, *J* = 7.6 Hz, 1H), 8.00 (d, *J* = 9.1 Hz, 1H), 7.80 – 7.64 (m, 5H), 7.57 (s, 4H), 7.48 (d, *J* = 7.5 Hz, 1H), 7.41 (t, *J* = 5.9 Hz, 2H), 7.05 (d, *J* = 8.2 Hz, 1H), 6.70 (t, *J* = 7.6 Hz, 1H), 6.43 (d, *J* = 7.9 Hz, 1H), 6.30 (t, *J* = 7.5 Hz, 1H), 5.75 (s, 1H). ^13^C NMR (101 MHz, DMSO-d_6_) δ_ppm_ 180.0, 179.8, 178.8, 145.3, 142.3, 141.0, 140.6, 135.3, 134.9, 133.9, 132.7, 132.2, 130.0, 127.3, 126.4, 125.8, 125.6, 125.2, 124.5, 123.7, 121.5, 119.3, 118.7, 111.4, 110.0, 102.1, 101.2, 49.7.

**3-(1*H*-Indol-3-yl)-6'-nitro-1,11-dihydrospiro[benzo[*g*]pyrazolo[3,4-*b*]quinoline-4,3'-indoline]-2',5,10-trione (C3).**

Black solid, M.p: >300 ˚C; (Ethyl acetate: *n*-Hexane 4:6), FT-IR (KBr, cm^-1^): 3401, 3302, 1732, 1670, 1560, 1340, 999, 725. ^1^H NMR (400 MHz, DMSO-d_6_) δ_ppm_ 12.67 (s, 1H), 11.25 (s, 1H), 10.88 (s, 1H), 10.69 (s, 1H), 8.06 (d, *J* = 5.7 Hz, 1H), 7.94 – 7.85 (m, 2H), 7.81 – 7.71 (m, 3H), 7.34 (d, *J* = 8.2 Hz, 1H), 7.07 (t, *J* = 7.7 Hz, 1H), 6.98 (d, *J* = 8.1 Hz, 1H), 6.86 (t, *J* = 7.5 Hz, 1H), 6.57 (d, *J* = 8.6 Hz, 1H), 6.40 (s, 1H). ^13^C NMR (101 MHz, DMSO-d_6_) δ_ppm_ 179.9, 179.9, 179.6, 148.4, 145.2, 142.7, 142.0, 139.0, 135.2, 134.9, 133.9, 132.7, 132.1, 130.1, 126.5, 126.2, 125.9, 125.5, 124.9, 124.8, 121.4, 119.1, 118.3, 111.4, 110.8, 108.4, 101.7, 49.4.

**3-(1*H*-Indol-3-yl)-1,11-dihydrospiro[benzo[*g*]pyrazolo[3,4-*b*]quinoline-4,11'-indeno[1,2-*b*]quinoxaline]-5,10-dione-methane (C4).**

Dark violet solid, M.p: >300 ˚C; (Ethyl acetate: *n*-Hexane 4:6), FT-IR (KBr, cm^-1^): 3435, 3251, 1663, 1562, 1514, 1339, 1295, 1101, 743. ^1^H NMR (400 MHz, DMSO-d_6_) δ_ppm_ 12.50 (s, 1H), 10.92 (s, 1H), 10.89 (s, 1H), 8.06 (d, *J* = 7.3 Hz, 1H), 7.89 (t, *J* = 7.7 Hz, 2H), 7.76 – 7.62 (m, 5H), 7.53 – 7.43 (m, 3H), 7.33 (t, *J* = 7.5 Hz, 1H), 7.07 (d, *J* = 8.2 Hz, 1H), 6.79 (t, *J* = 7.6 Hz, 1H), 6.55 (d, *J* = 7.9 Hz, 1H), 6.42 (t, *J* = 7.5 Hz, 1H), 5.52 (s, 1H). ^13^C NMR (101 MHz, DMSO-d_6_) δ_ppm_ 180.6, 180.1, 168.5, 155.5, 141.5, 141.2, 136.1, 135.4, 135.2, 133.2, 132.5, 132.3, 130.4, 129.24, 129.0, 128.8, 128.2, 126.4, 126.3, 126.0, 125.4, 121.6, 121.1, 119.0, 118.4, 111.4, 102.4, 44.7.

**3-(1*H*-Indol-3-yl)-7'-nitro-1,11-dihydrospiro[benzo[*g*]pyrazolo[3,4-*b*]quinoline-4,11'-indeno[1,2-*b*]quinoxaline]-5,10-dione--methane (C5).**

Dark violet solid, M.p: >300 ˚C; (Ethyl acetate: *n*-Hexane 4:6), FT-IR (KBr, cm^-1^): 3427, 1670, 1579, 1514, 1344, 1077, 993, 738.^1^H NMR (400 MHz, DMSO-d_6_) δ_ppm_ 12.58 (s, 1H), 11.04 (s, 1H), 10.91 (s, 1H), 8.67 (s, 1H), 8.35 (d, 1H), 8.07 (d, *J* = 7.6 Hz, 1H), 8.00 (d, *J* = 9.1 Hz, 1H), 7.80 – 7.64 (m, 5H), 7.57 (s, 4H), 7.48 (d, *J* = 7.5 Hz, 1H), 7.41 (t, *J* = 5.9 Hz, 1H), 7.05 (d, *J* = 8.2 Hz, 1H), 6.70 (t, *J* = 7.6 Hz, 1H), 6.43 (d, *J* = 7.9 Hz, 1H), 6.30 (t, *J* = 7.5 Hz, 1H), 5.75 (s, 1H). ^13^C NMR (101 MHz, DMSO-d_6_) δ_ppm_ 180.5, 180.2, 170.5, 157.5, 156.0, 146.5, 145.7, 140.0, 135.4, 135.2, 134.9, 134.3, 133.7, 133.3, 132.4, 130.5, 130.4, 128.6, 126.7, 126.6, 126.4, 126.0, 125.6, 124.9, 124.8, 122.6, 121.9, 121.6, 118.9, 118.2, 111.3, 105.1, 102.0, 50.2.

**3-(1*H*-indol-3-yl)-7'-methyl-1,11-dihydrospiro[benzo[*g*]pyrazolo[3,4-*b*]quinoline-4,11'-Indeno[1,2-*b*]quinoxaline]-5,10-dione-methane (C6).**

Dark violet solid, M.p: >300 ˚C; (Ethyl acetate: *n*-Hexane 4:6), FT-IR (KBr, cm^-1^): 3430, 1665, 1596, 1563, 1338, 1296, 991, 724. ^1^H NMR (400 MHz, DMSO-d_6_) δ_ppm_ 12.48 (s, 1H), 10.89 (d, *J* = 5.1 Hz, 2H), 8.05 (d, *J* = 7.5 Hz, 1H), 7.85 – 7.74 (m, 2H), 7.74 – 7.69 (m, 3H), 7.69 – 7.62 (m, 2H), 7.56 – 7.46 (m, 4H), 7.42 (t, *J* = 7.4 Hz, 2H), 7.31 (t, *J* = 7.5 Hz, 1H), 7.09 (d, *J* = 8.2 Hz, 1H), 6.83 (t, *J* = 5.4 Hz, 1H), 6.62 (d, *J* = 8.1 Hz, 1H), 6.50 (t, *J* = 7.3 Hz, 1H), 5.46 (s, 1H), 2.45 (s, 3H). ^13^C NMR (101 MHz, DMSO-d_6_) δ_ppm_ 180.1, 179.6, 167.9, 167.2, 155.0, 154.8, 154.2, 153.6, 142.6, 141.0, 139.0, 138.7, 135.7, 134.9, 134.7, 132.7, 132.0, 131.7, 130.4, 129.9, 128.0, 127.8, 127.5, 125.8, 125.8, 125.5, 124.9, 123.7, 121.1, 120.6, 118.7, 118.0, 110.9, 49.7, 21.1.

**10-(1*H*-indol-3-yl)-7,8-dihydro-6*H*-spiro[chromeno[3,4-*b*]pyrazolo[4,3-*e*]pyridine-11,3'-Indoline]-2',6-dione (D1).**

Light cream solid, M.p: >300 ˚C; (Ethyl acetate: *n*-Hexane 4:6), FT-IR (KBr, cm^-1^): 3408, 3305, 1724, 1614, 1472, 1348, 1107, 753. ^1^H NMR (400 MHz, DMSO-d_6_) δ_ppm_ 12.64 (s, 1H), 11.25 (s, 1H), 11.09 (s, 1H), 10.66 (s, 1H), 8.52 (d, *J* = 7.8 Hz, 1H), 7.92 – 7.87 (m, 2H), 7.68 (d, 1H), 7.45 (d, *J* = 8.2 Hz, 1H), 7.36 – 7.33 (m, 2H), 7.08 (d, *J* = 7.1 Hz, 2H), 6.89 (s, 1H), 6.55 (d, *J* = 8.6 Hz, 1H), 6.39 (s, 1H), 5.81 (s, 1H). ^13^C NMR (101 MHz, DMSO-d_6_) δ_ppm_ 179.6, 159.5, 151.9, 148.7, 146.5, 146.5, 142.0, 138.4, 135.2, 126.4, 124.9, 124.1, 123.9, 123.4, 121.5, 119.1, 118.8, 118.3, 116.7, 113.5, 111.4, 108.4, 100.6, 93.9, 49.2.

**6'-Chloro-10-(1*H*-indol-3-yl)-7,8-dihydro-6*H*-spiro[chromeno[3,4-*b*]pyrazolo[4,3-*e*]pyridine-11,3'-indoline]-2',6-dione (D2).**

Light cream solid, M.p: >300 ˚C; (Ethyl acetate: *n*-Hexane 4:6), FT-IR (KBr, cm^-1^): 3377, 3240, 1712, 1702, 1689, 1616, 1478, 751. ^1^H NMR (400 MHz, DMSO-d_6_) δ_ppm_ 12.55 (s, 1H), 11.25 (s, 1H), 10.47 (s, 1H), 10.11 (s, 1H), 8.49 (d, *J* = 7.7 Hz, 1H), 7.91 (d, *J* = 7.8 Hz, 1H), 7.67 (t, *J* = 7.8 Hz, 1H), 7.46 – 7.43 (m, 1H), 7.35 (t, *J* = 8.1 Hz, 2H), 7.13 (s, 1H), 6.99 – 6.97 (m, 1H), 6.88 (d, *J* = 8.1 Hz, 1H), 6.51 (d, *J* = 8.1 Hz, 2H), 6.10 (s, 1H). ^13^C NMR (101 MHz, DMSO-d_6_) δ_ppm_ 178.7, 166.9, 159.3, 151.9, 149.0, 146.1, 145.8, 142.1, 141.3, 140.0, 135.3, 132.2, 131.4, 130.6, 126.3, 125.3, 124.4, 123.3, 121.5, 119.3, 116.6, 113.5, 111.4, 102.4, 102.2, 56.7.

**10'-(1*H*-Indol-3-yl)-7',8'-dihydro-2*H*,6'*H*-spiro[acenaphthylene-1,11'-chromeno[3,4-*b*]pyrazolo[4,3-*e*]pyridine]-2,6'-dione (D3).**

Light cream solid, M.p: >300 ˚C; (Ethyl acetate: *n*-Hexane 4:6), FT-IR (KBr, cm^-1^): 3369, 3052, 1724, 1571, 1456, 1343, 1145, 781, 744. ^1^H NMR (400 MHz, DMSO-d_6_) δ_ppm_ 12.25 (s, 1H), 10.80 (s, 1H), 10.75 (s, 1H), 8.07 (d, *J* = 8.2 Hz, 1H), 7.92 (s, 1H), 7.77 (t, *J* = 7.5 Hz, 1H), 7.64 – 7.55 (m, 2H), 7.29 (t, *J* = 7.3 Hz, 1H), 7.22 – 7.18 (m, 1H), 7.15 – 7.11 (m, 1H), 7.08 – 7.03 (m, 2H), 6.92 (t, *J* = 7.0 Hz, 1H), 6.84 (d, *J* = 8.3 Hz, 1H), 6.73 (t, *J* = 7.6 Hz, 1H), 6.65 (t, *J* = 7.6 Hz, 1H), 5.53 (s, 1H). ^13^C NMR (101 MHz, DMSO-d_6_) δ_ppm_ 180.4, 168.1, 155.3, 154.8, 146.0, 143.2, 141.7, 141.0, 138.6, 136.0, 135.4, 133.3, 132.6, 132.4, 130.4, 130.2, 129.7, 129.2, 129.0, 128.8, 127.8, 126.4, 126.1, 125.6, 121.6, 113.7, 104.4, 58.5.


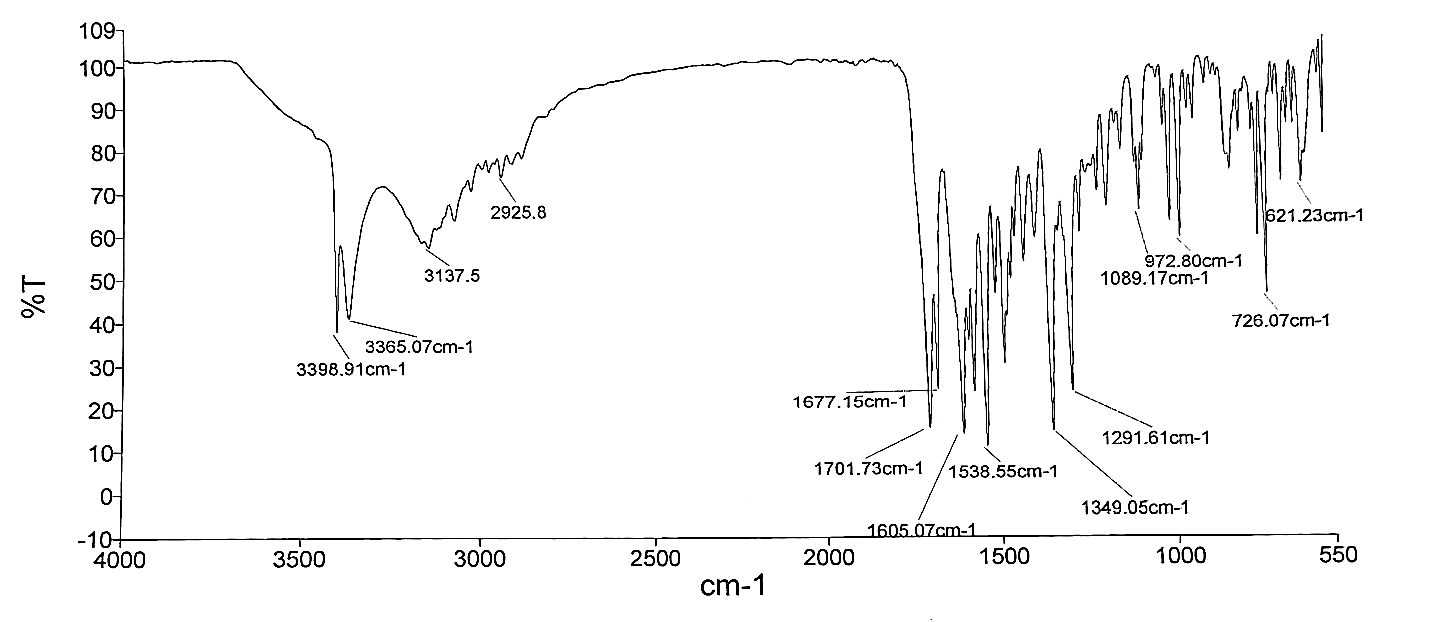


# Supplementary Figure 1: FT-IR spectrum of 3-(4-chlorophenyl)-1,11-dihydrospiro[benzo[g]pyrazolo[3,4-b]quinoline-4,3'-indoline]-2',5,10-trione (A1).

*
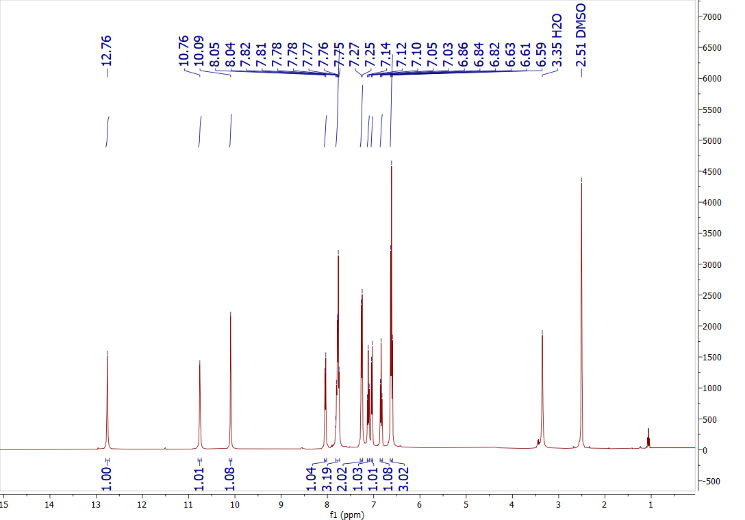
*

# Supplementary Figure 2: ^1^HNMR spectrum of 3-(4-chlorophenyl)-1,11-dihydrospiro[benzo[g]pyrazolo[3,4-b]quinoline-4,3'-indoline]-2',5,10-trione (A1).

*
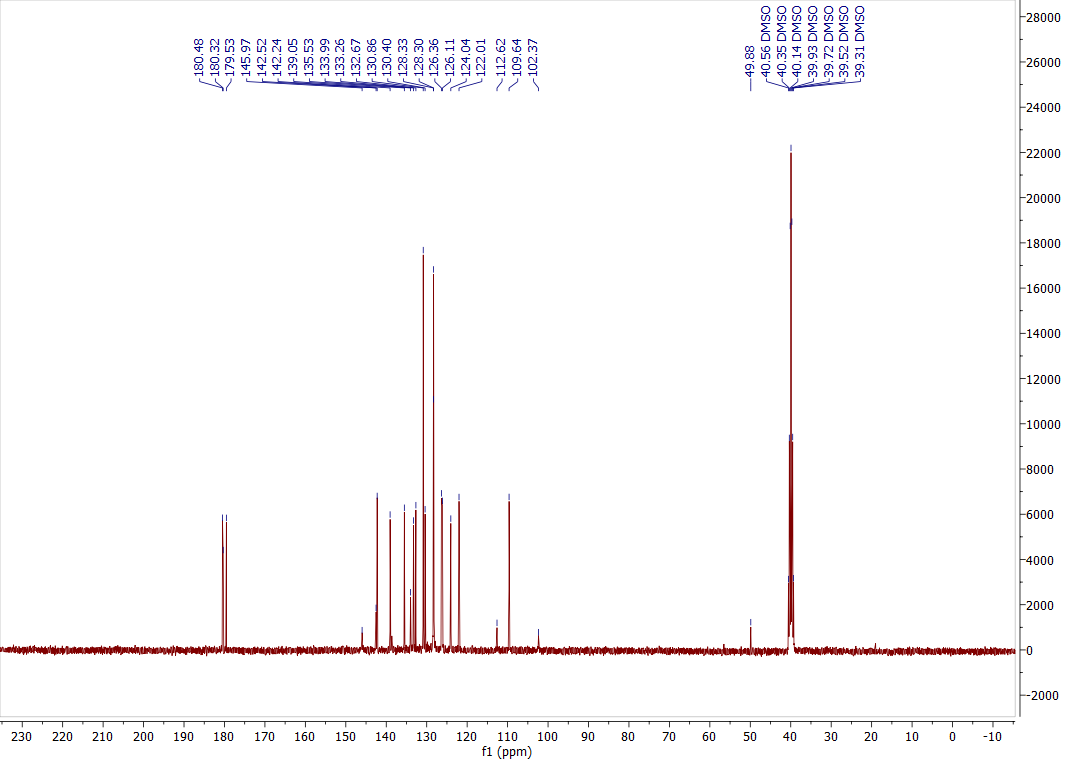
*

# Supplementary Figure 3: ^13^CNMR spectrum of 6'-chloro-3-(4-chlorophenyl)-1,11-dihydrospiro[benzo[g]pyrazolo[3,4-b]quinoline-4,3'-indoline]-2',5,10-trione (A2).

*
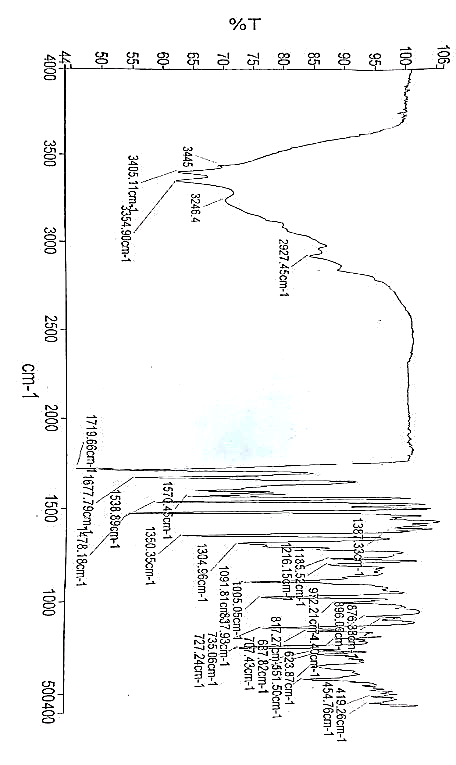
*

# Supplementary Figure 4: FT-IR spectrum of 6'-chloro-3-(4-chlorophenyl)-1,11-dihydrospiro[benzo[g]pyrazolo[3,4-b]quinoline-4,3'-indoline]-2',5,10-trione (A2).

*
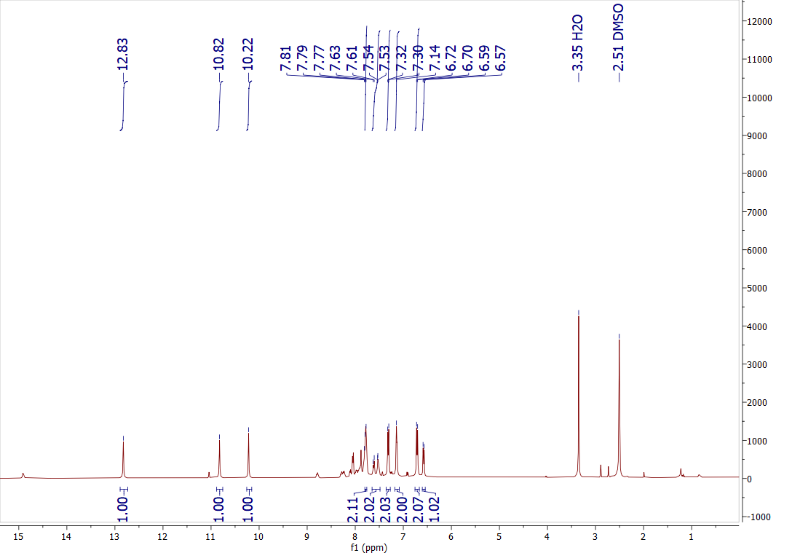
*

# Supplementary Figure 5: ^1^HNMR spectrum of 6'-chloro-3-(4-chlorophenyl)-1,11-dihydrospiro[benzo[g]pyrazolo[3,4-b]quinoline-4,3'-indoline]-2',5,10-trione (A2).

*
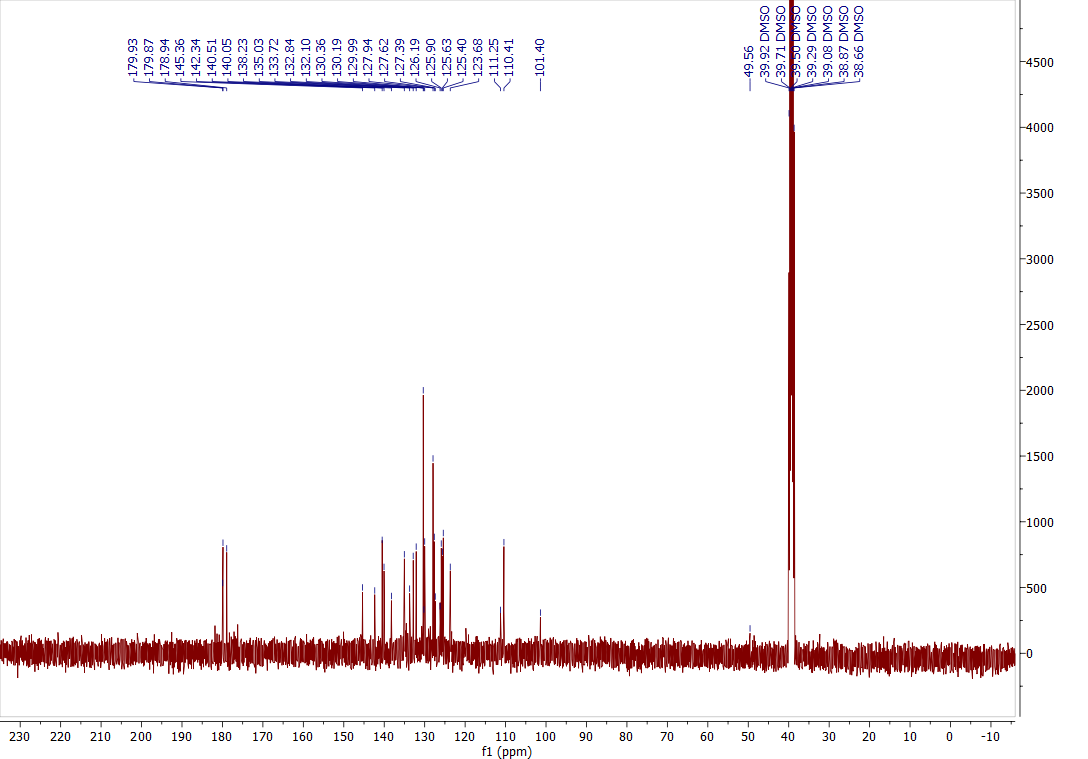
*

# Supplementary Figure 6: ^13^CNMR spectrum of 6'-chloro-3-(4-chlorophenyl)-1,11-dihydrospiro[benzo[g]pyrazolo[3,4-b]quinoline-4,3'-indoline]-2',5,10-trione (A2).

*
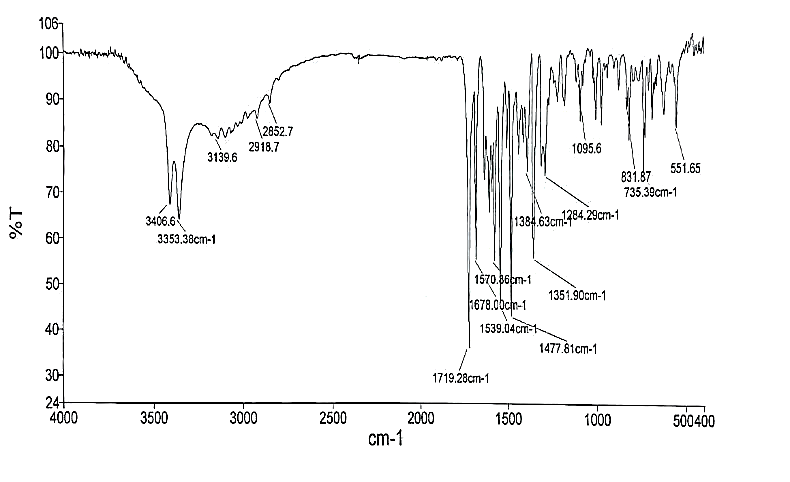
*

# Supplementary Figure 7: FT-IR spectrum of 3-(4-chlorophenyl)-6'-nitro-1,11-dihydrospiro[benzo[g]pyrazolo[3,4-b]quinoline-4,3'-indoline]-2',5,10-trione (A3).

*
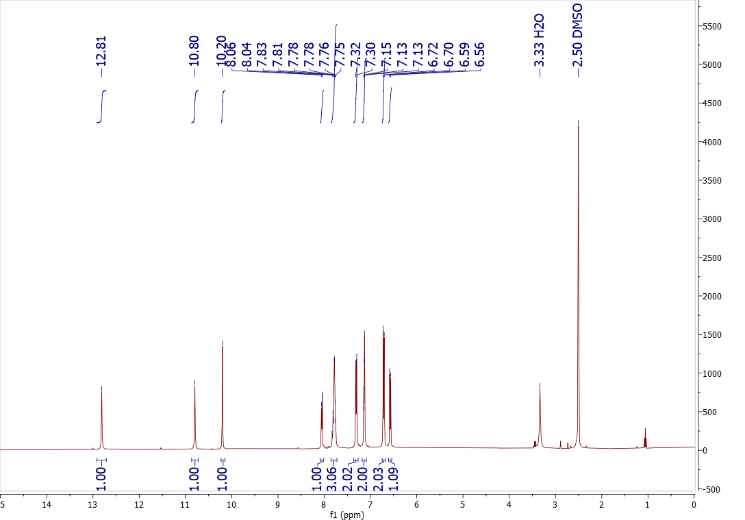
*

# Supplementary Figure 8: ^1^HNMR spectrum of 3-(4-chlorophenyl)-6'-nitro-1,11-dihydrospiro[benzo[g]pyrazolo[3,4-b]quinoline-4,3'-indoline]-2',5,10-trione (A3).

*
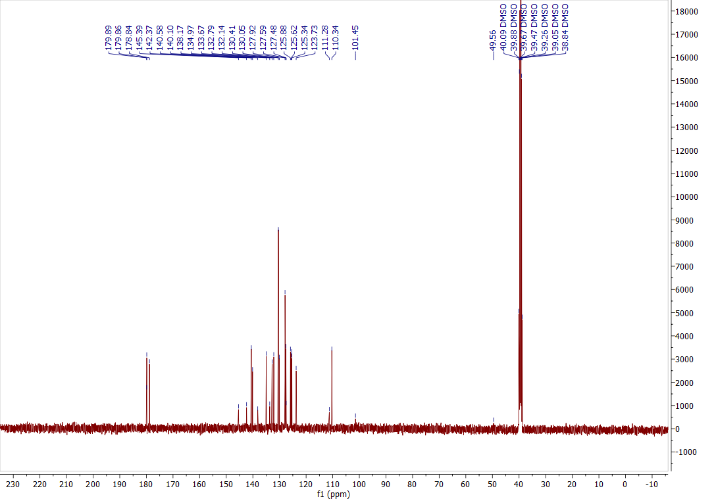
*

# Supplementary Figure 9: ^13^CNMR spectrum of 3-(4-chlorophenyl)-6'-nitro-1,11-dihydrospiro[benzo[g]pyrazolo[3,4-b]quinoline-4,3'-indoline]-2',5,10-trione (A3).

*
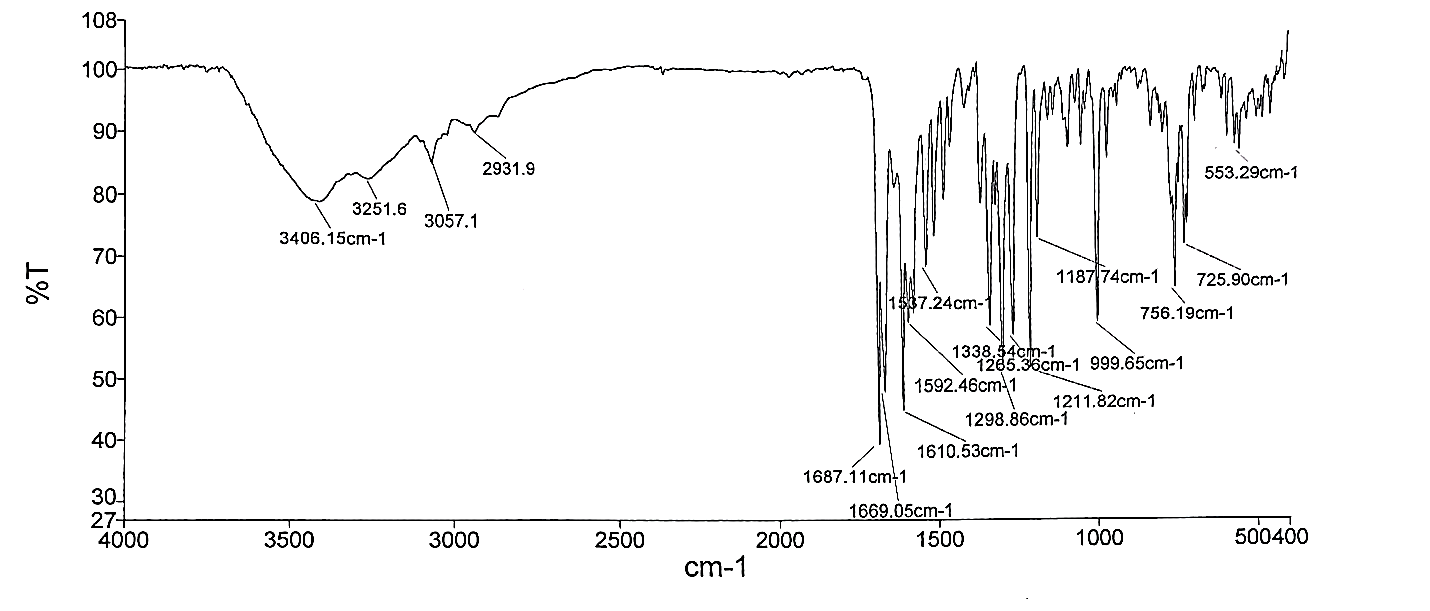
*

# Supplementary Figure 10: FT-IR spectrum of 3-(4-chlorophenyl)-1,11-dihydrospiro[benzo[g]pyrazolo[3,4-b]quinoline-4,11'-indeno[1,2-b]quinoxaline]-5,10-dione (A4).

*
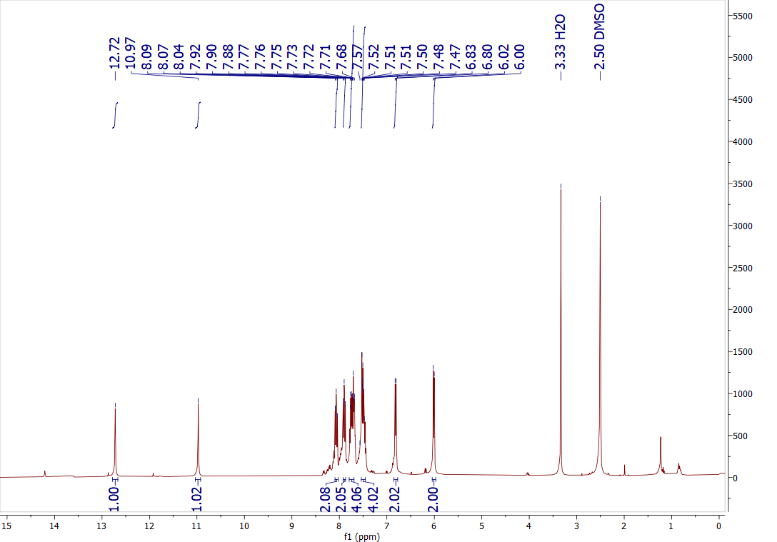
*

# Supplementary Figure 11: ^1^HNMR spectrum of 3-(4-chlorophenyl)-1,11-dihydrospiro[benzo[g]pyrazolo[3,4-b]quinoline-4,11'-indeno[1,2-b]quinoxaline]-5,10-dione (A4).

*
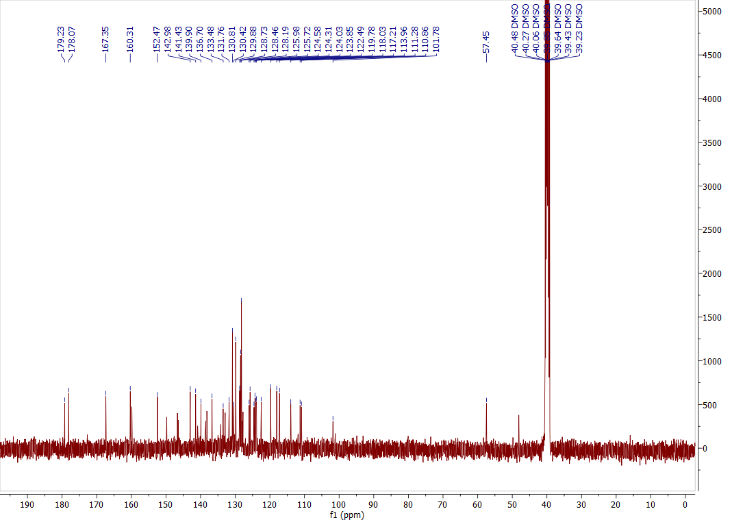
*

Supplementary Figure 12: ^13^CNMR spectrum of 3-(4-chlorophenyl)-1,11-dihydrospiro[benzo[*g*]pyrazolo[3,4-*b*]quinoline-4,11'-indeno[1,2-*b*]quinoxaline]-5,10-dione (A4).

*
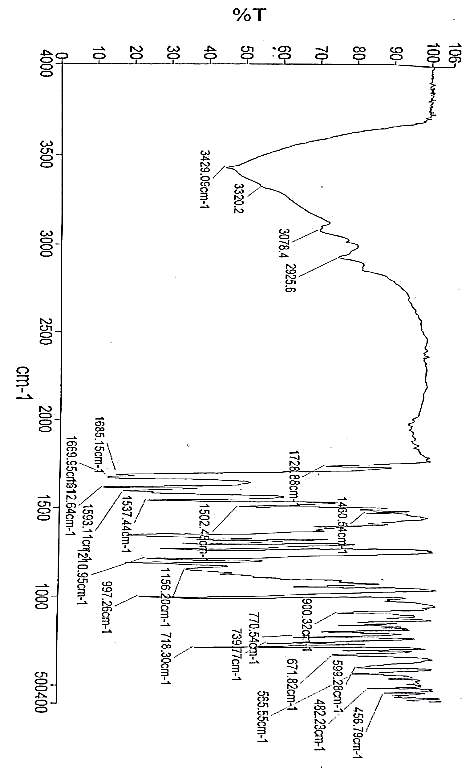
*

# Supplementary Figure 13: FT-IR spectrum of 3-(4-chlorophenyl)-7'-nitro-1,11-dihydrospiro[benzo[g]pyrazolo[3,4-b]quinoline-4,11'-indeno[1,2-b]quinoxaline]-5,10-dione (A5).

 *
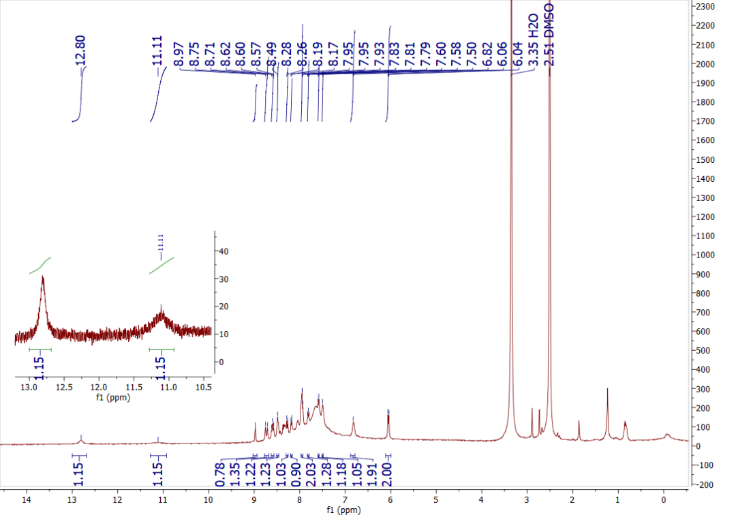
*

# Supplementary Figure 14: ^1^HNMR spectrum of 3-(4-chlorophenyl)-7'-nitro-1,11-dihydrospiro[benzo[g]pyrazolo[3,4-b]quinoline-4,11'-indeno[1,2-b]quinoxaline]-5,10-dione (A5).

*
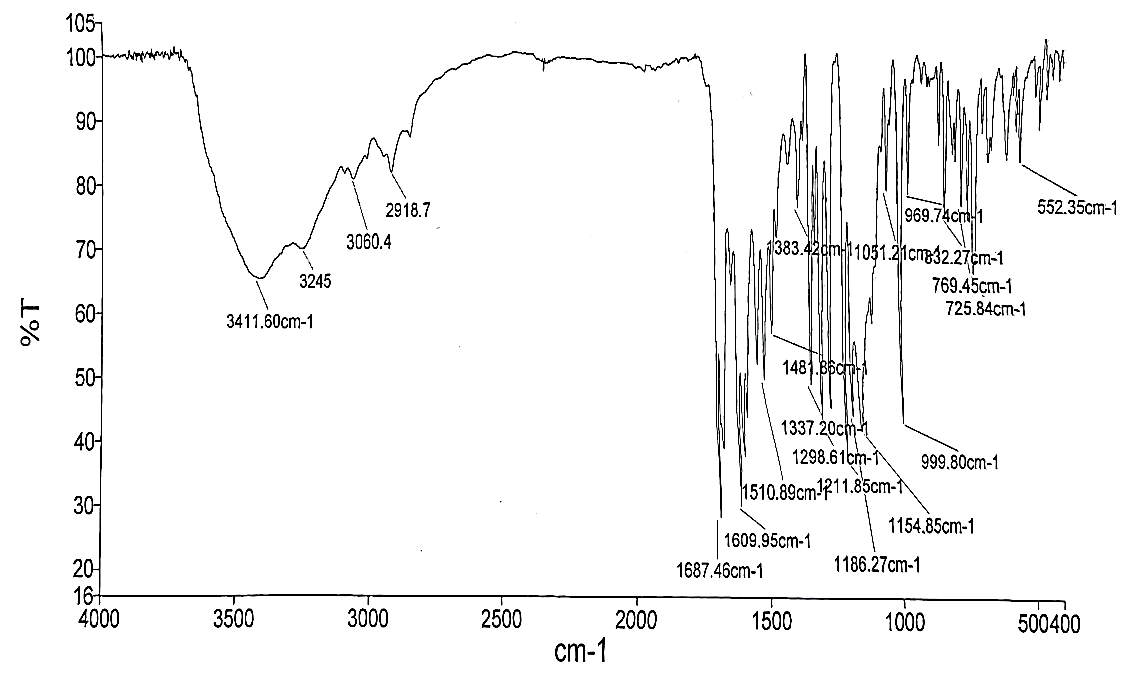
*

# Supplementary Figure 15: FT-IR spectrum of 3-(4-chlorophenyl)-7'-methyl-1,11-dihydrospiro[benzo[g]pyrazolo[3,4-b]quinoline-4,11'-indeno[1,2-b]quinoxaline]-5,10-dione (A6).

*
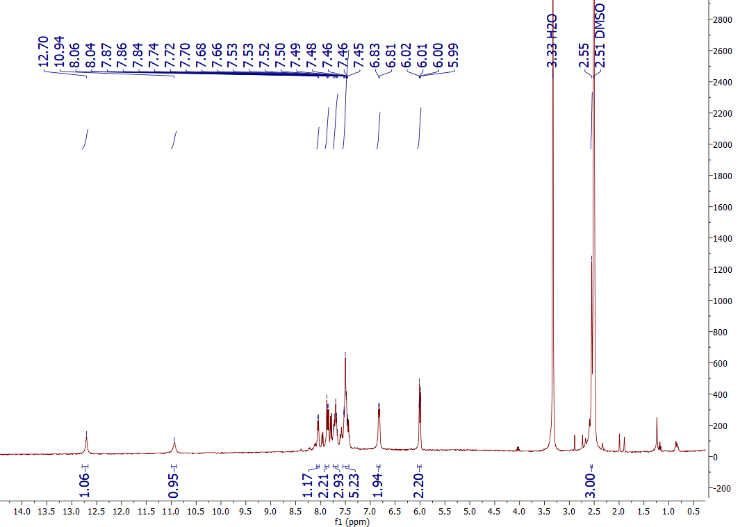
*

Supplementary Figure 16: ^1^HNMR spectrum of 3-(4-chlorophenyl)-7'-methyl-1,11-dihydrospiro[benzo[*g*]pyrazolo[3,4-*b*]quinoline-4,11'-indeno[1,2-*b*]quinoxaline]-5,10-dione (A6).

*
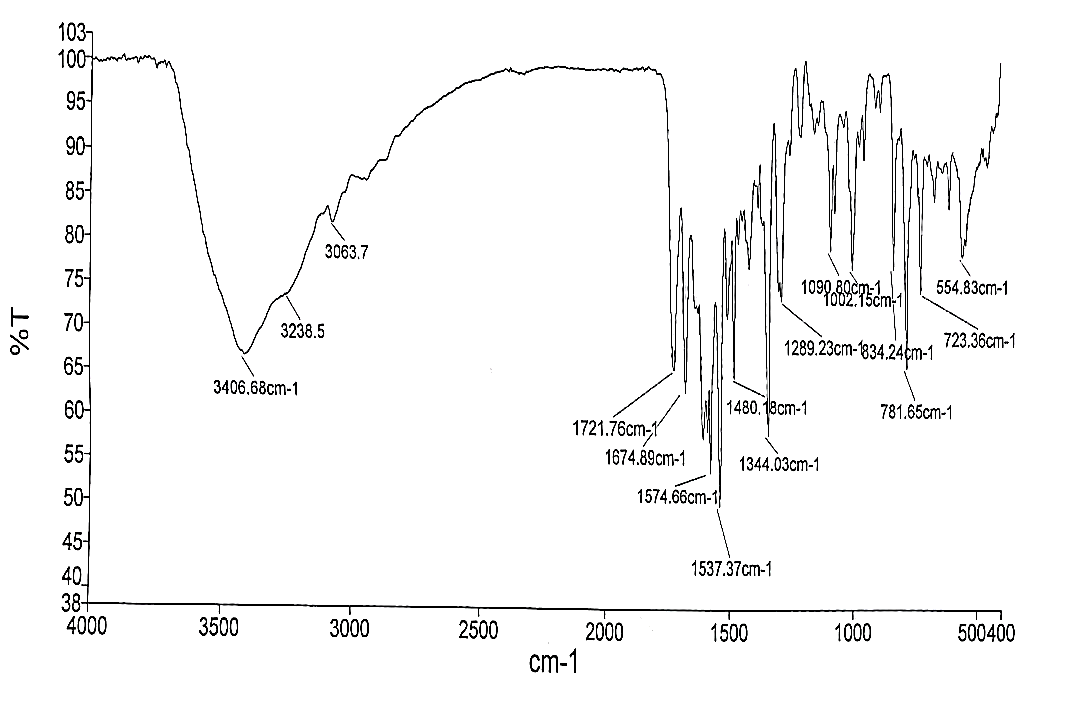
*

# Supplementary Figure 17: FT-IR spectrum of 3-(4-chlorophenyl)-1',11'-dihydro-2H-spiro[acenaphthylene-1,4'-benzo[g]pyrazolo[3,4-b]quinoline]-2,5',10'-trione (A7).

*
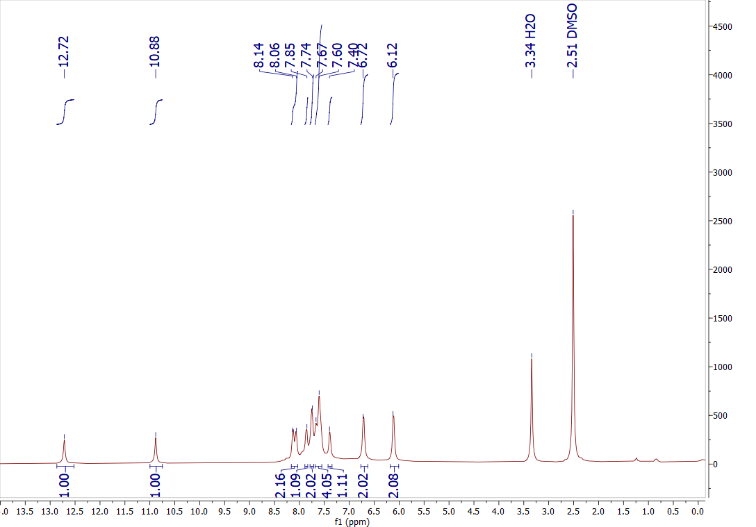
*

# Supplementary Figure 18: ^1^HNMR spectrum of 3-(4-chlorophenyl)-1',11'-dihydro-2H-spiro[acenaphthylene-1,4'-benzo[g]pyrazolo[3,4-b]quinoline]-2,5',10'-trione (A7).

*
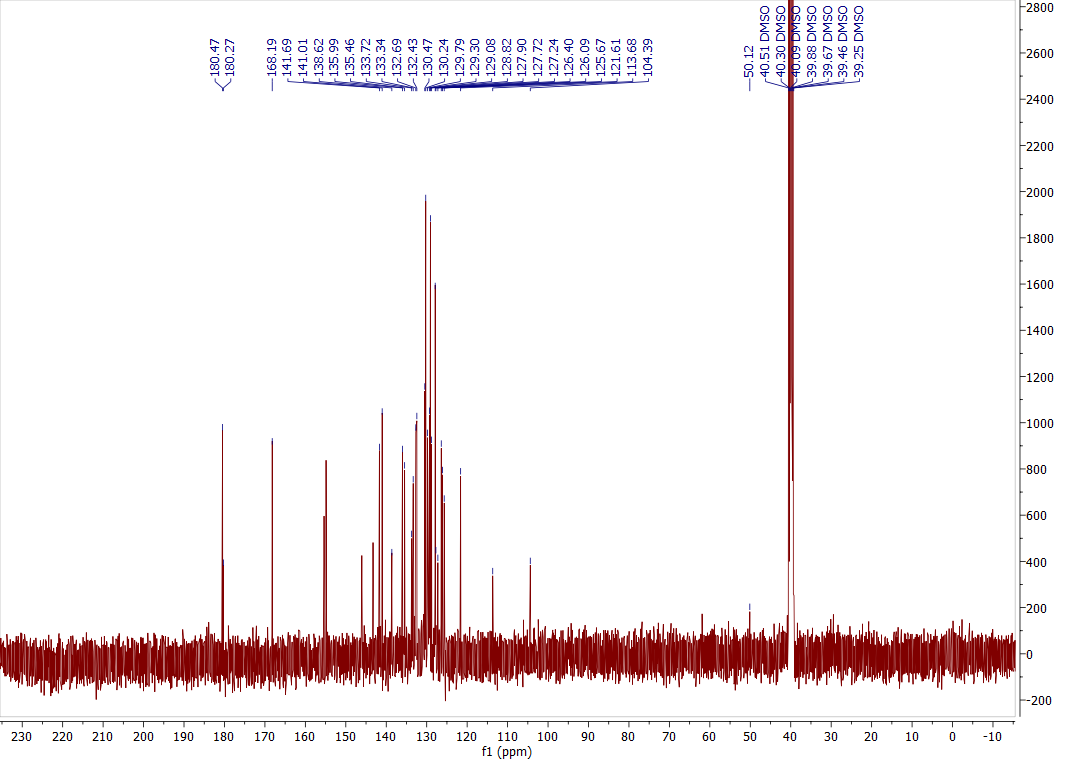
*

# Supplementary Figure 19: ^13^CNMR spectrum of 3-(4-chlorophenyl)-1',11'-dihydro-2H-spiro[acenaphthylene-1,4'-benzo[g]pyrazolo[3,4-b]quinoline]-2,5',10'-trione (A7).

*
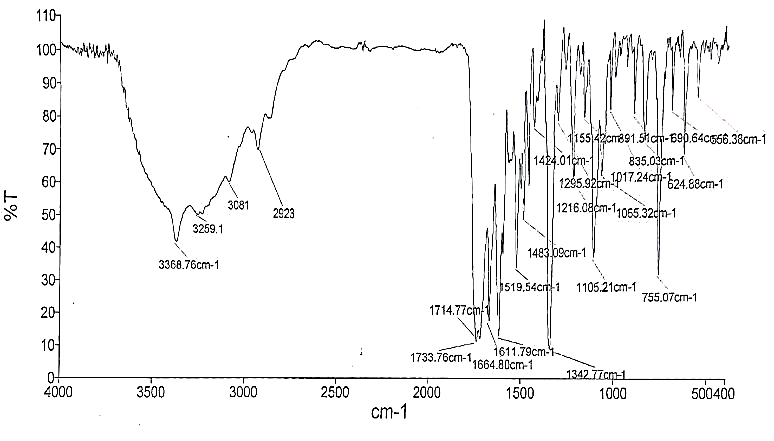
*

# Supplementary Figure 20: FT-IR spectrum of 10-(4-chlorophenyl)-6'-nitro-7,8-dihydro-6H-spiro[chromeno[3,4-b]pyrazolo[4,3-e]pyridine-11,3'-indoline]-2',6-dione (B1).

*
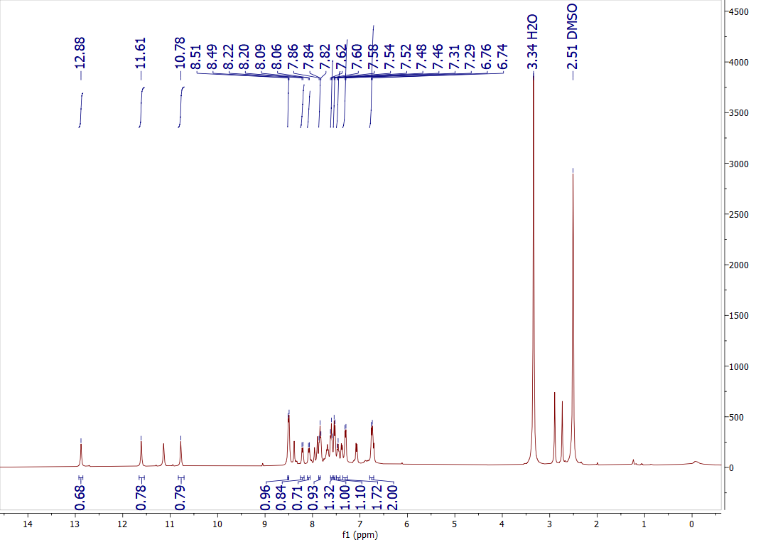
*

# Supplementary Figure 21: ^1^HNMR spectrum of 10-(4-chlorophenyl)-6'-nitro-7,8-dihydro-6H-spiro[chromeno[3,4-b]pyrazolo[4,3-e]pyridine-11,3'-indoline]-2',6-dione (B1).

*
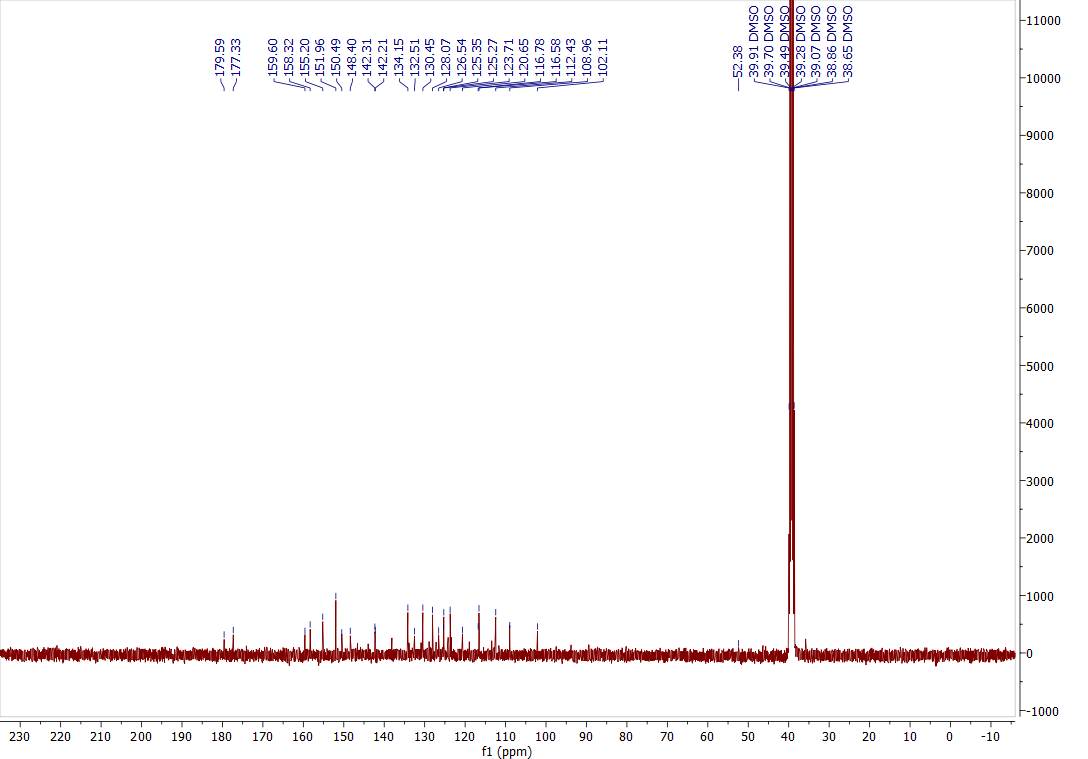
*

# Supplementary Figure 22: ^13^CNMR spectrum of 10-(4-chlorophenyl)-6'-nitro-7,8-dihydro-6H-spiro[chromeno[3,4-b]pyrazolo[4,3-e]pyridine-11,3'-indoline]-2',6-dione (B1).

*
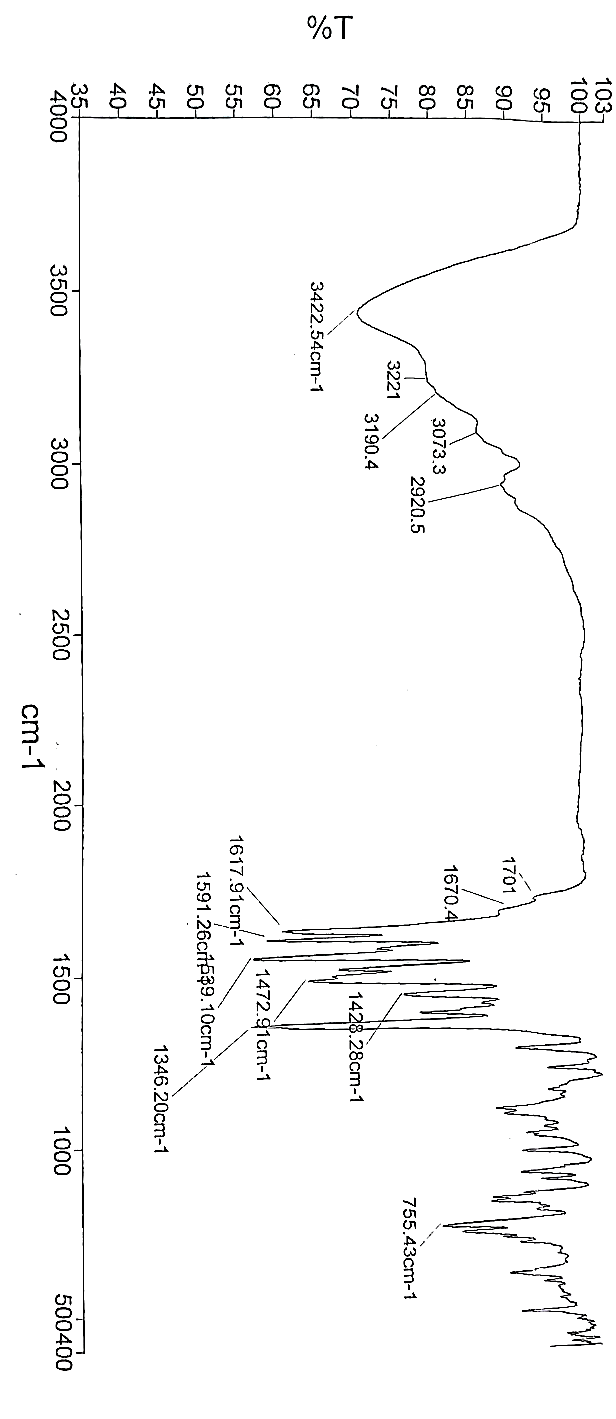
*

# Supplementary Figure 23: FT-IR spectrum of 10-(4-chlorophenyl)-7'-nitro-7,8-dihydro-6H-spiro[chromeno[3,4-b]pyrazolo[4,3-e]pyridine-11,11'-indeno[1,2-b]quinoxalin]-6-one (B2).

*
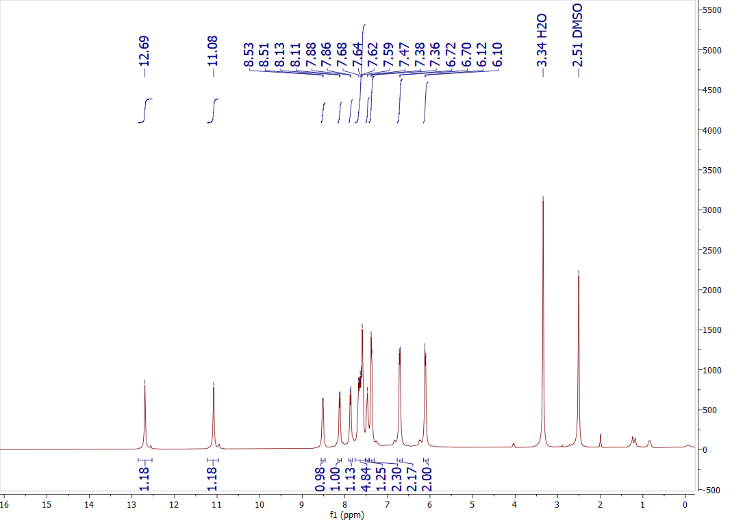
*

# Supplementary Figure 24: ^1^HNMR spectrum of 10-(4-chlorophenyl)-7'-nitro-7,8-dihydro-6H-spiro[chromeno[3,4-b]pyrazolo[4,3-e]pyridine-11,11'-indeno[1,2-b]quinoxalin]-6-one (B2).

*
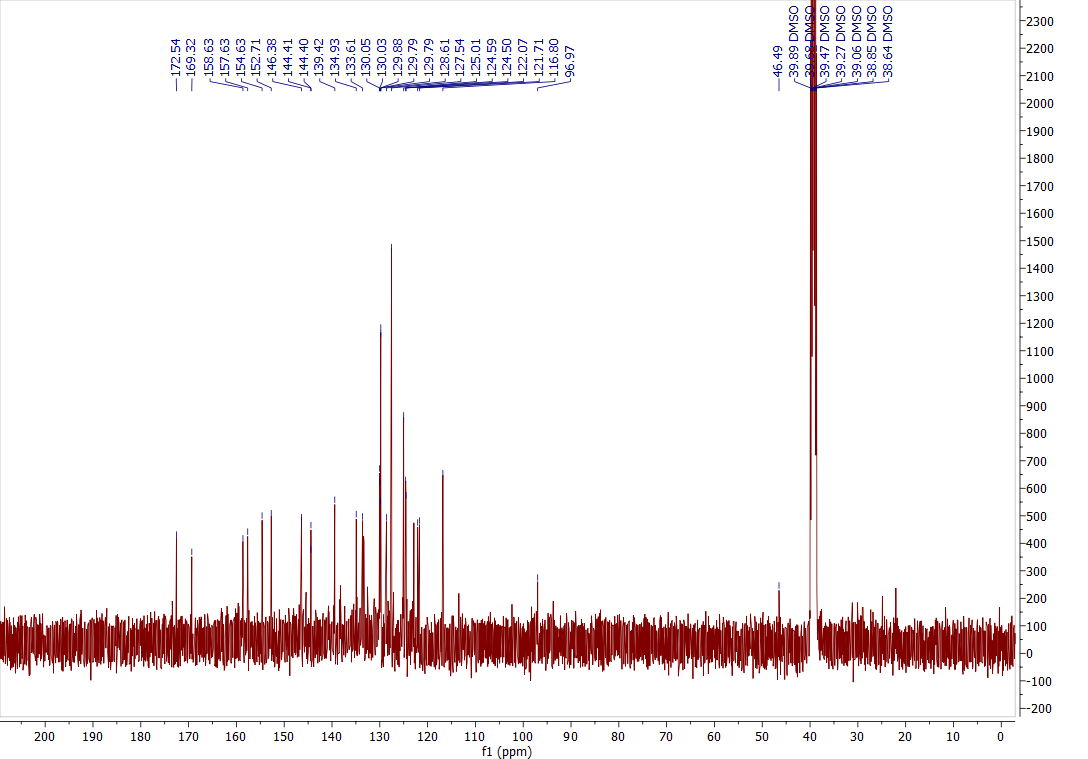
*

# Supplementary Figure 25: ^13^CNMR spectrum of 10-(4-chlorophenyl)-7'-nitro-7,8-dihydro-6H-spiro[chromeno[3,4-b]pyrazolo[4,3-e]pyridine-11,11'-indeno[1,2-b]quinoxalin]-6-one (B2).

*
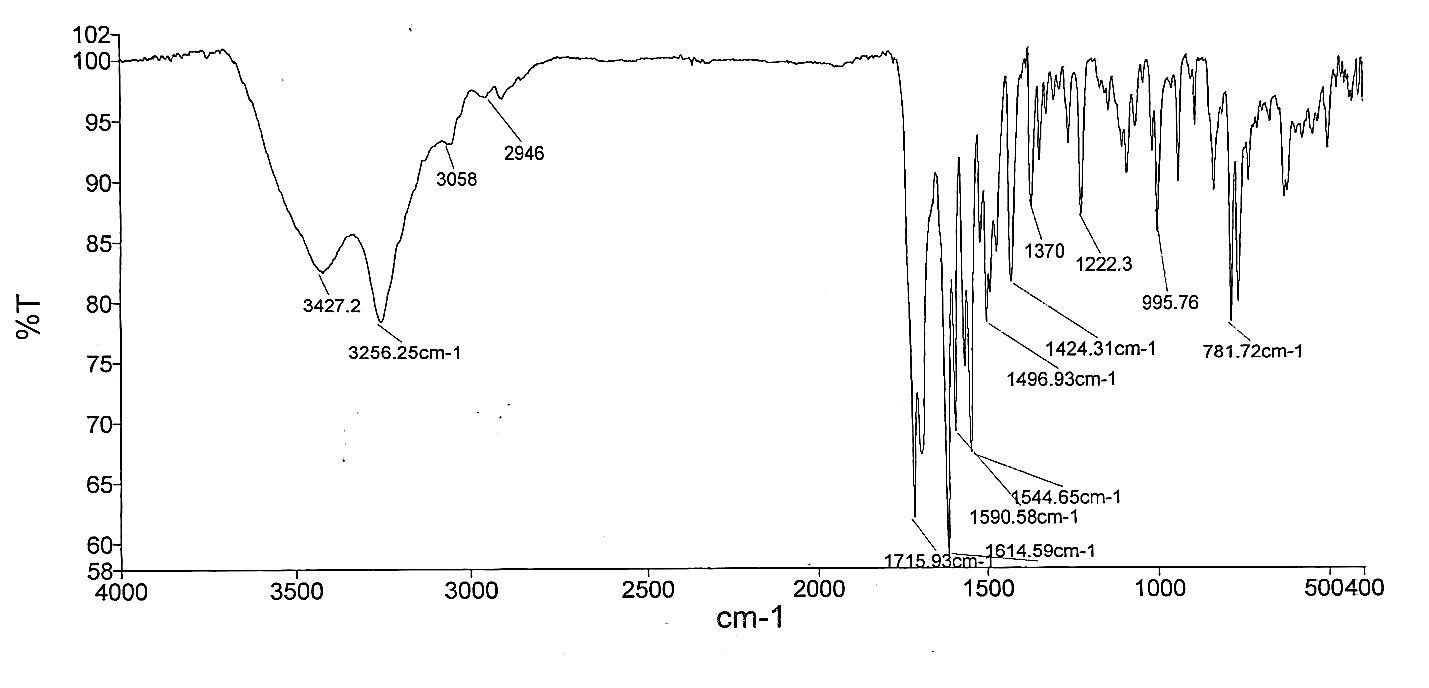
*

# Supplementary Figure 26: FT-IR spectrum of 10-(4-chlorophenyl)-7',8'-dihydro-2H,6'H-spiro[acenaphthylene-1,11'-chromeno[3,4-b]pyrazolo[4,3-e]pyridine]-2,6'-dione (B3).

*
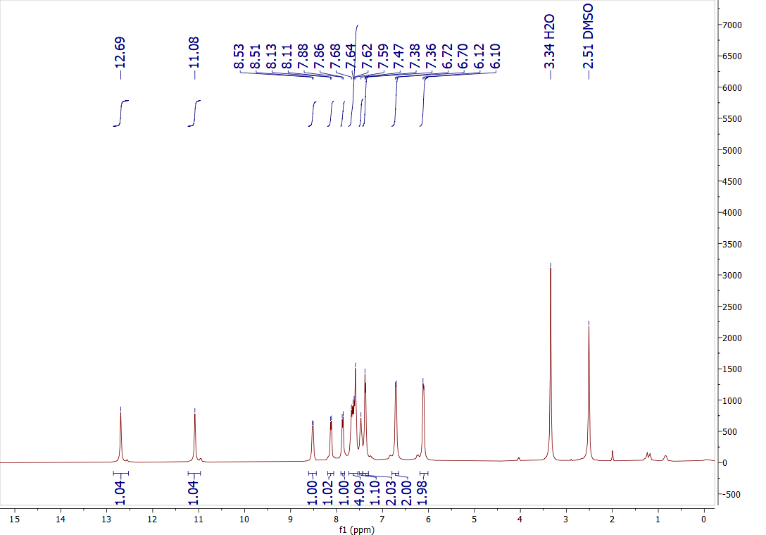
*

# Supplementary Figure 27: ^1^HNMR spectrum of 10-(4-chlorophenyl)-7',8'-dihydro-2H,6'H-spiro[acenaphthylene-1,11'-chromeno[3,4-b]pyrazolo[4,3-e]pyridine]-2,6'-dione (B3).

*
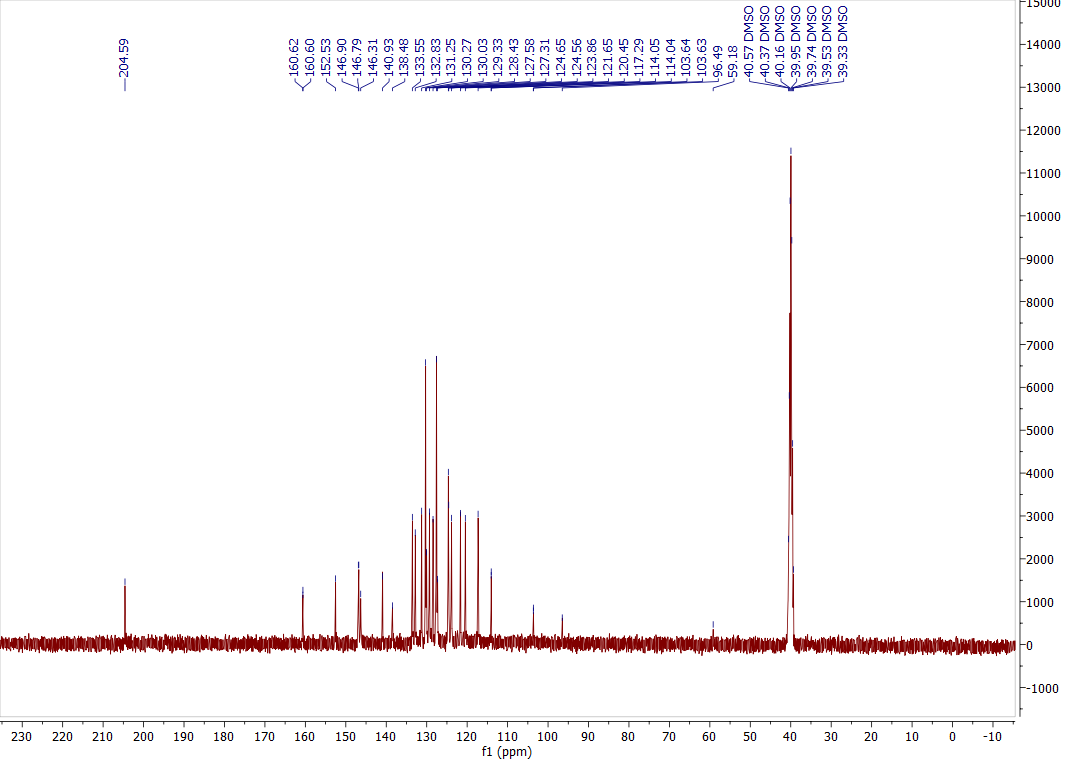
*

# Supplementary Figure 28: ^13^CNMR spectrum of 10-(4-chlorophenyl)-7',8'-dihydro-2H,6'H-spiro[acenaphthylene-1,11'-chromeno[3,4-b]pyrazolo[4,3-e]pyridine]-2,6'-dione (B3).

*
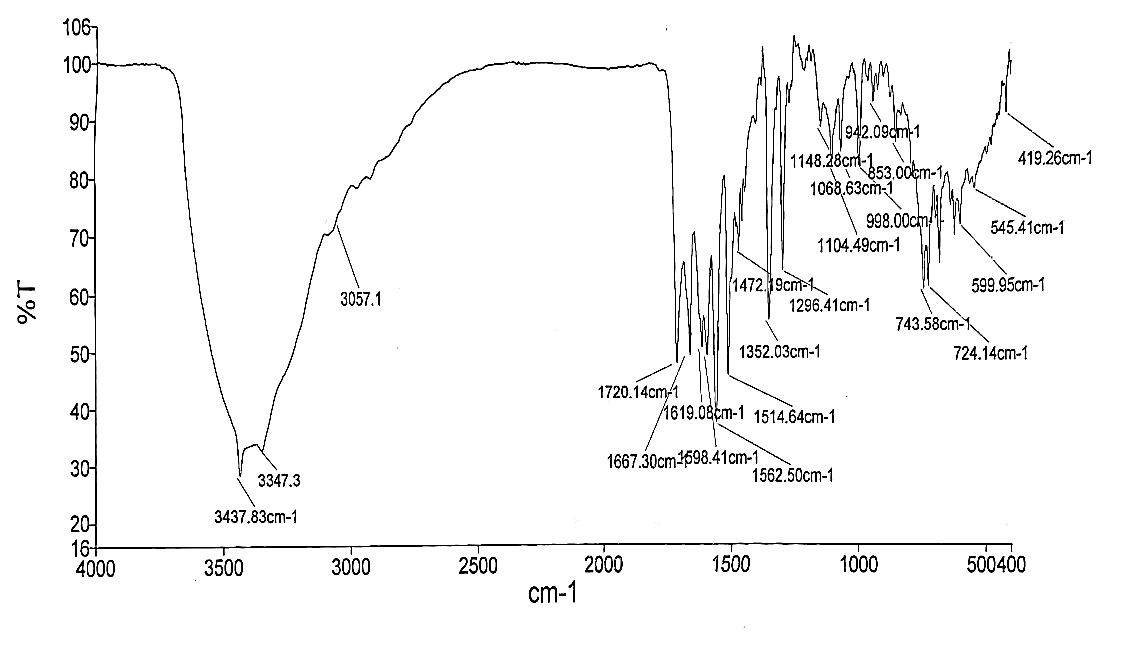
*

# Supplementary Figure 29: FT-IR spectrum of 3-(1H-indol-3-yl)-1,11-dihydrospiro[benzo[g]pyrazolo[3,4-b]quinoline-4,3'-indoline]-2',5,10-trione (C1).

*
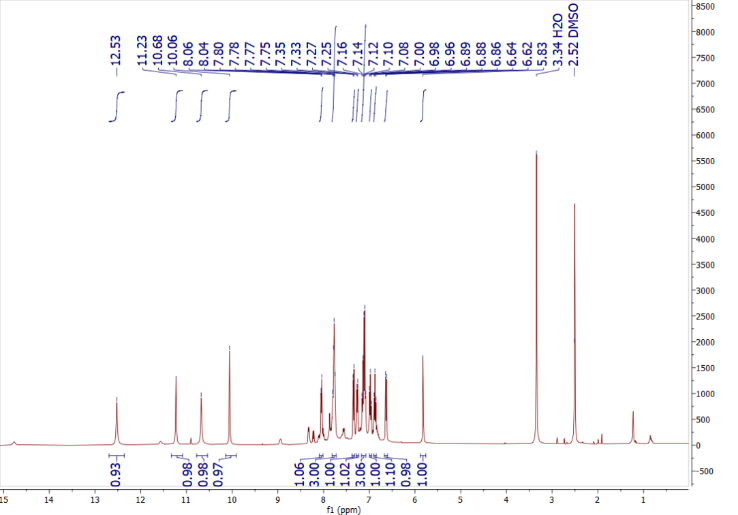
*

# Supplementary Figure 30: ^1^HNMR spectrum of 3-(1H-indol-3-yl)-1,11-dihydrospiro[benzo[g]pyrazolo[3,4-b]quinoline-4,3'-indoline]-2',5,10-trione (C1).


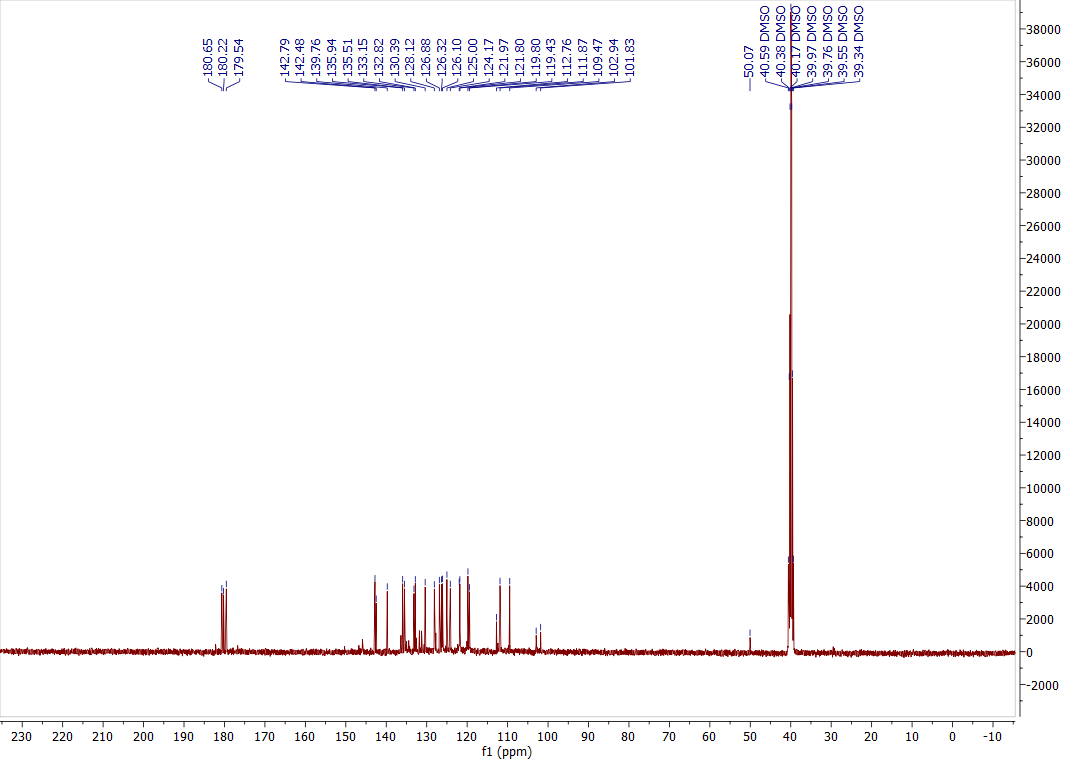


# Supplementary Figure 31: ^13^CNMR spectrum of 3-(1H-indol-3-yl)-1,11-dihydrospiro[benzo[g]pyrazolo[3,4-b]quinoline-4,3'-indoline]-2',5,10-trione (C1).

*
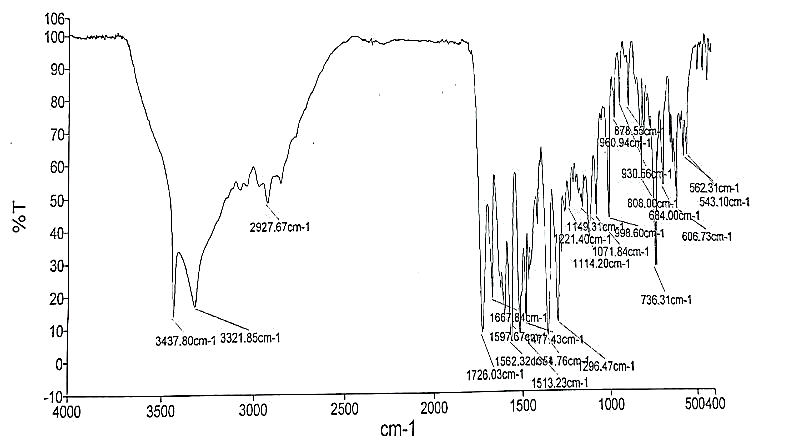
*

# Supplementary Figure 32: FT-IR spectrum of 6'-chloro-3-(1H-indol-3-yl)-1,11-dihydrospiro[benzo[g]pyrazolo[3,4-b]quinoline-4,3'-indoline]-2',5,10-trioe (C2).

*
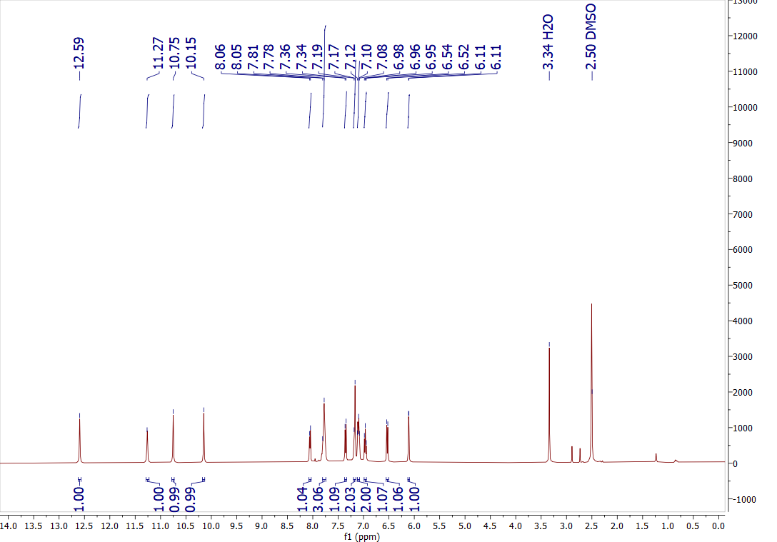
*

# Supplementary Figure 33: ^1^HNMR spectrum of 6'-chloro-3-(1H-indol-3-yl)-1,11-dihydrospiro[benzo[g]pyrazolo[3,4-b]quinoline-4,3'-indoline]-2',5,10-trioe (C2).

*
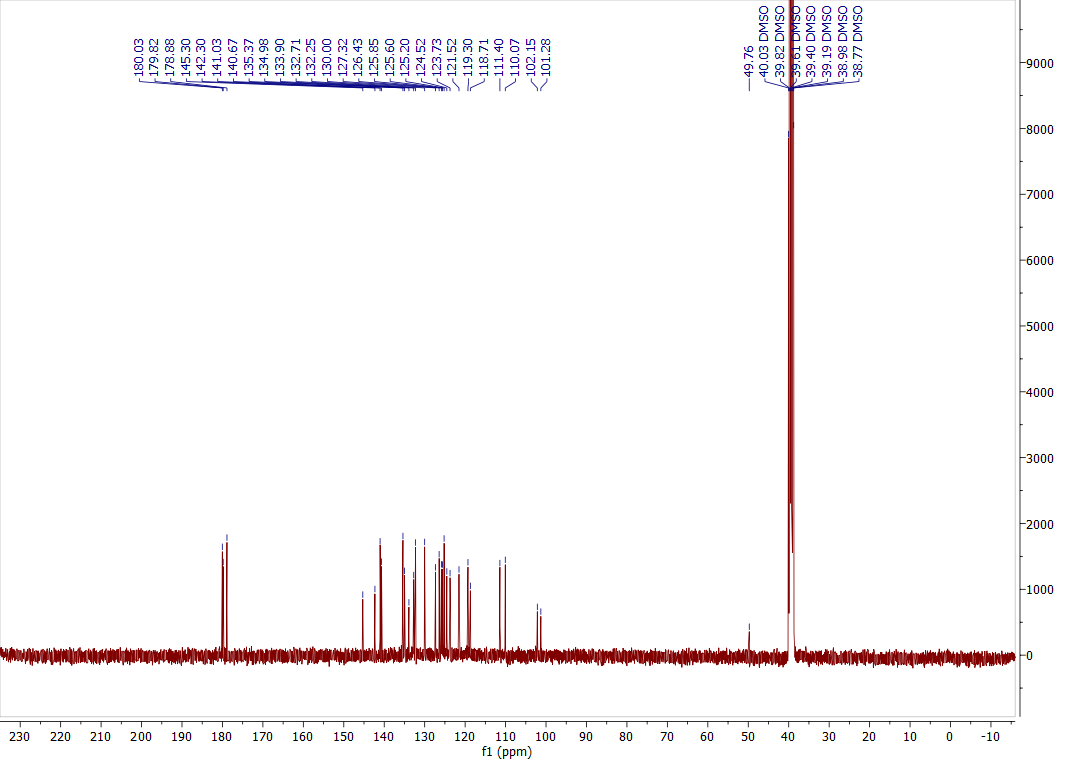
*

# Supplementary Figure 34: ^13^CNMR spectrum of 6'-chloro-3-(1H-indol-3-yl)-1,11-dihydrospiro[benzo[g]pyrazolo[3,4-b]quinoline-4,3'-indoline]-2',5,10-trioe (C2).

*
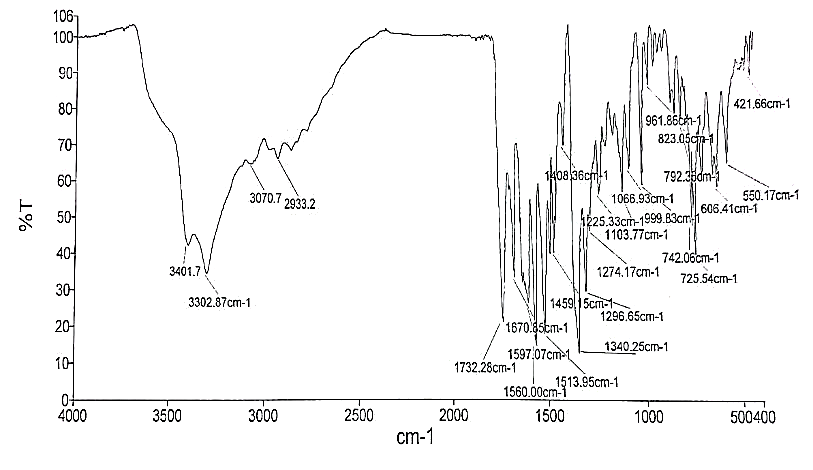
*

# Supplementary Figure 35: FT-IR spectrum of 3-(1H-indol-3-yl)-6'-nitro-1,11-dihydrospiro[benzo[g]pyrazolo[3,4-b]quinoline-4,3'-indoline]-2',5,10-trione (C3).

*
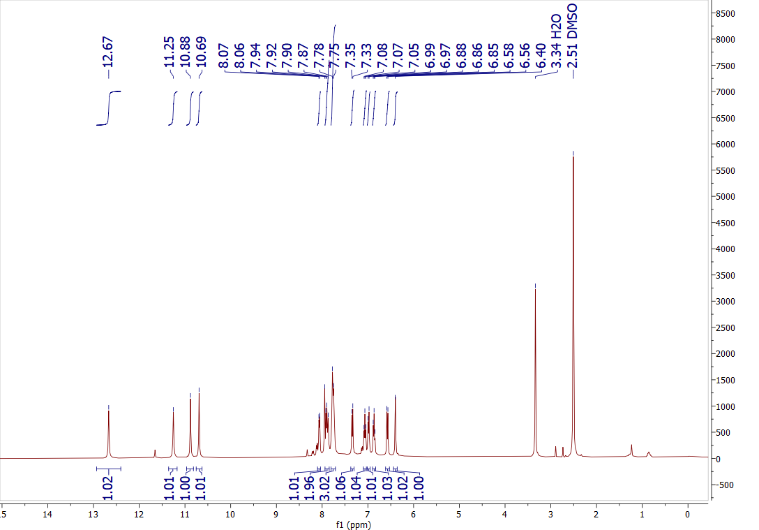
*

# Supplementary Figure 36: ^1^HNMR spectrum of 3-(1H-indol-3-yl)-6'-nitro-1,11-dihydrospiro[benzo[g]pyrazolo[3,4-b]quinoline-4,3'-indoline]-2',5,10-trione (C3).

*
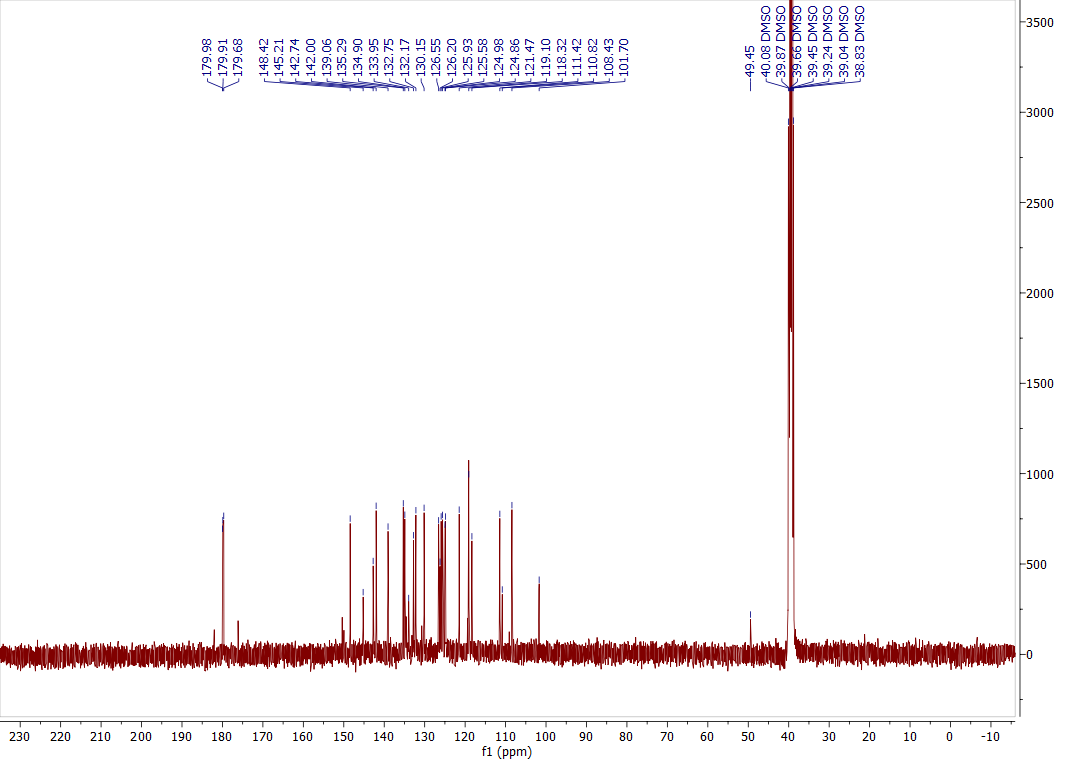
*

# Supplementary Figure 37: ^13^CNMR spectrum of 3-(1H-indol-3-yl)-6'-nitro-1,11-dihydrospiro[benzo[g]pyrazolo[3,4-b]quinoline-4,3'-indoline]-2',5,10-trione (C3).

 *
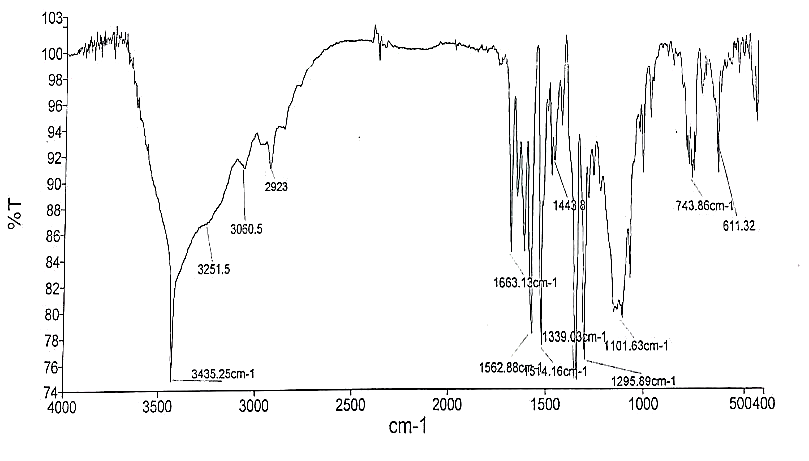
*

# Supplementary Figure 38: FT-IR spectrum of 3-(1H-indol-3-yl)-1,11-dihydrospiro[benzo[g]pyrazolo[3,4-b]quinoline-4,11'-indeno[1,2-b]quinoxaline]-5,10-dione--methane (C4).

*
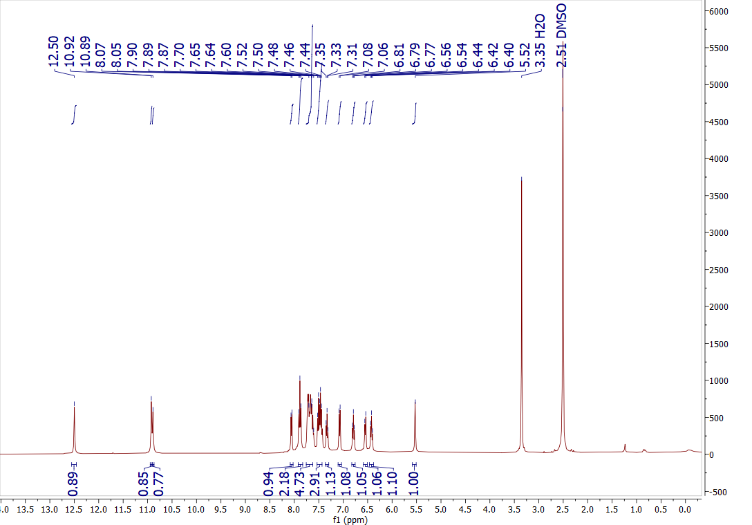
*

# Supplementary Figure 39: ^1^HNMR spectrum of 3-(1H-indol-3-yl)-1,11-dihydrospiro[benzo[g]pyrazolo[3,4-b]quinoline-4,11'-indeno[1,2-b]quinoxaline]-5,10-dione--methane (C4).

*
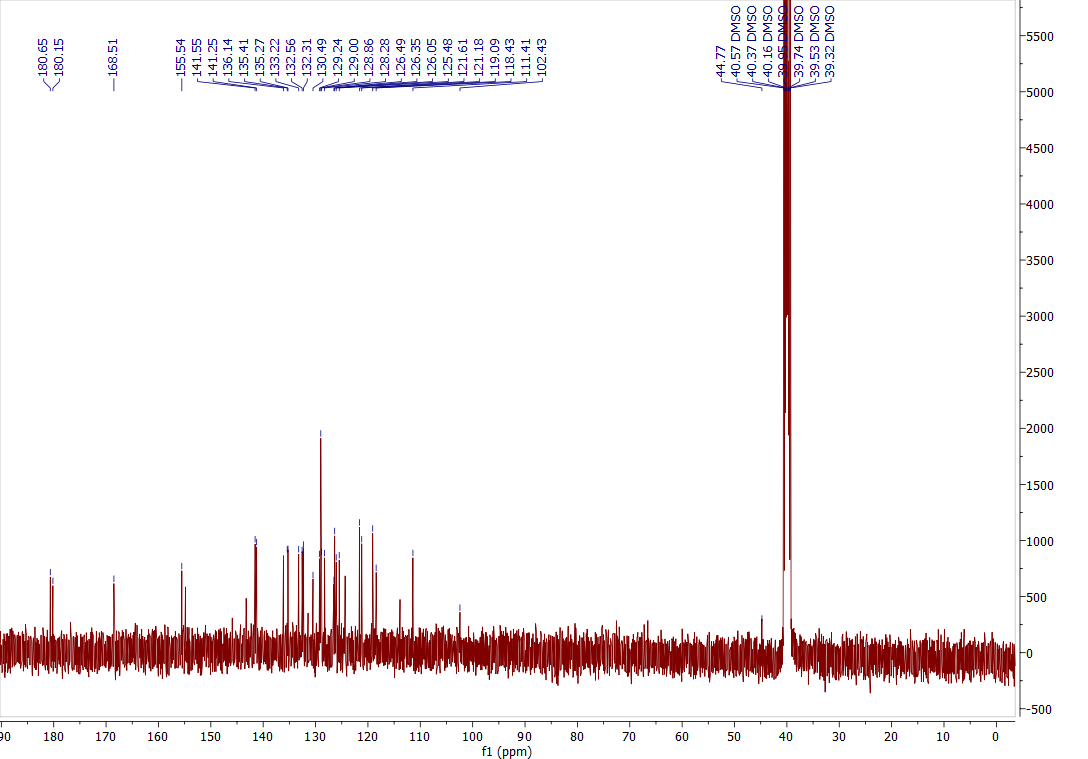
*

# Supplementary Figure 40: ^13^CNMR spectrum of 3-(1H-indol-3-yl)-1,11-dihydrospiro[benzo[g]pyrazolo[3,4-b]quinoline-4,11'-indeno[1,2-b]quinoxaline]-5,10-dione--methane (C4).

*
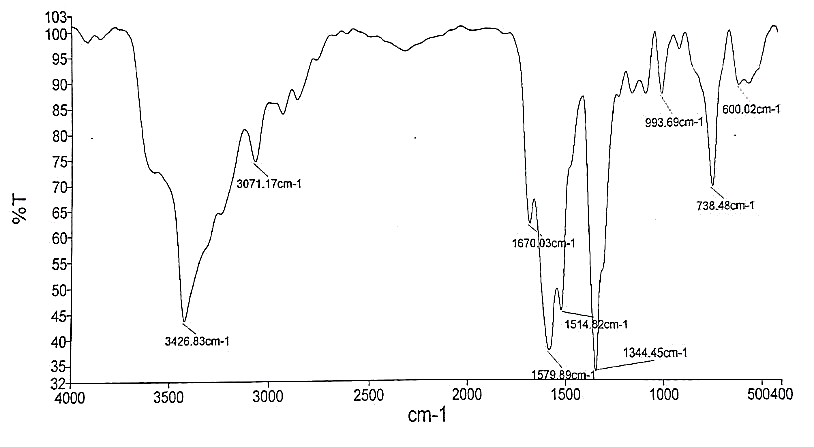
*

# Supplementary Figure 41: FT-IR spectrum of 3-(1H-indol-3-yl)-7'-nitro-1,11-dihydrospiro[benzo[g]pyrazolo[3,4-b]quinoline-4,11'-indeno[1,2-b]quinoxaline]-5,10-dione--methane (C5).

*
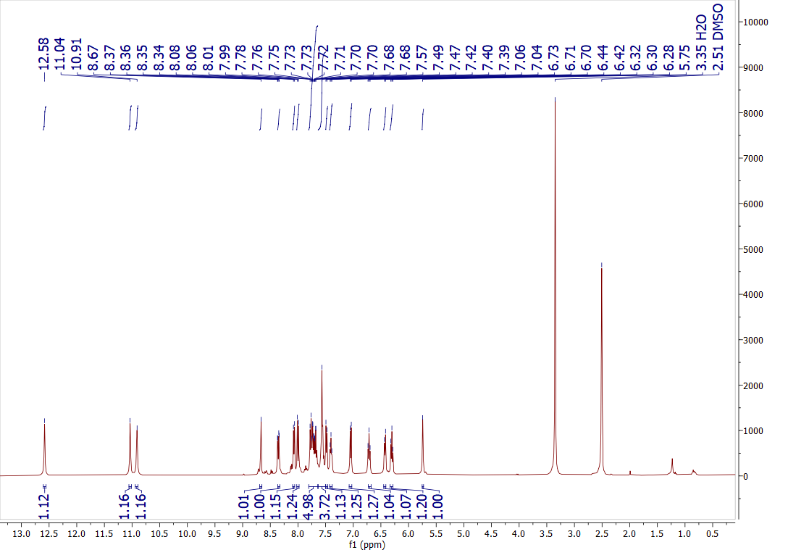
*

# Supplementary Figure 42: ^1^HNMR spectrum of 3-(1H-indol-3-yl)-7'-nitro-1,11-dihydrospiro[benzo[g]pyrazolo[3,4-b]quinoline-4,11'-indeno[1,2-b]quinoxaline]-5,10-dione--methane (C5).

*
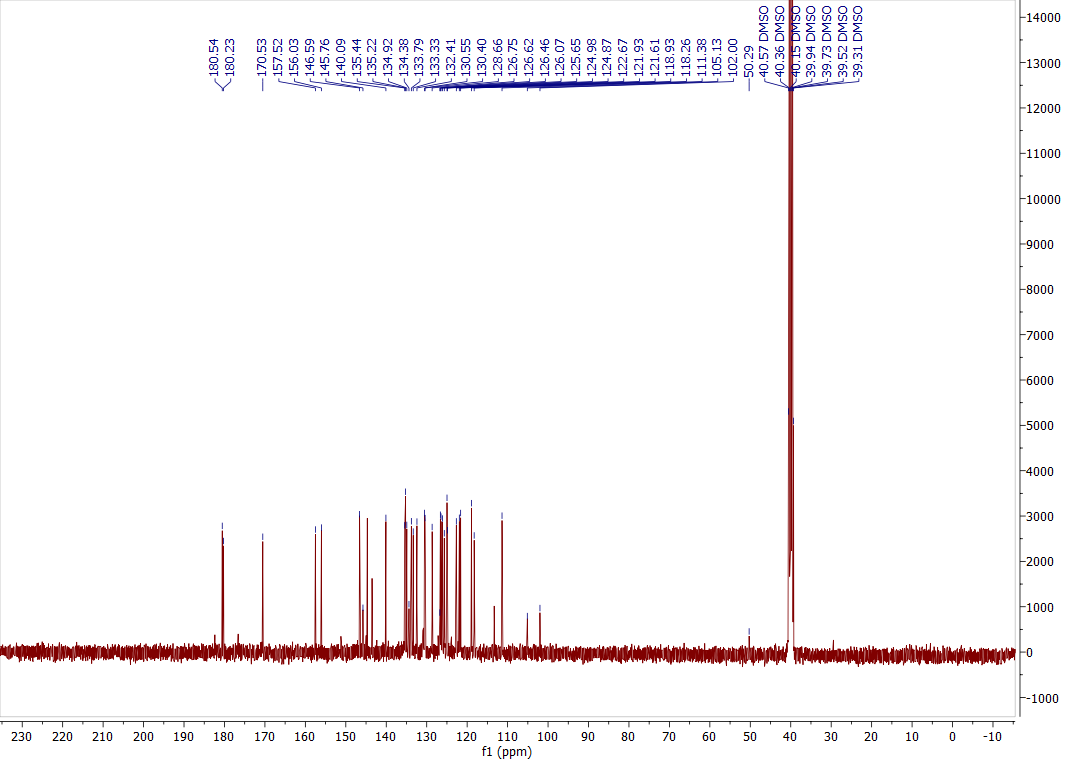
*

# Supplementary Figure 43: ^13^CNMR spectrum of 3-(1H-indol-3-yl)-7'-nitro-1,11-dihydrospiro[benzo[g]pyrazolo[3,4-b]quinoline-4,11'-indeno[1,2-b]quinoxaline]-5,10-dione—methane (C5).

*
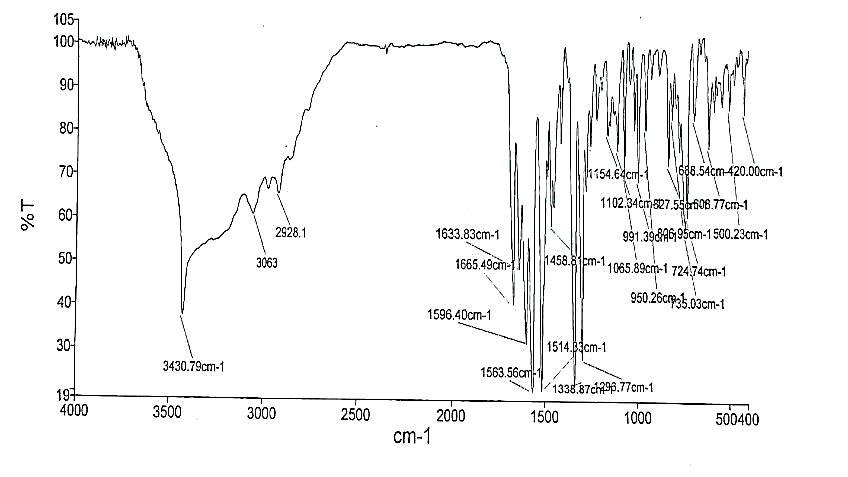
*

# Supplementary Figure 44: FT-IR spectrum of 3-(1H-indol-3-yl)-7'-methyl-1,11-dihydrospiro[benzo[g]pyrazolo[3,4-b]quinoline-4,11'-indeno[1,2-b]quinoxaline]-5,10-dione--methane (C6).

*
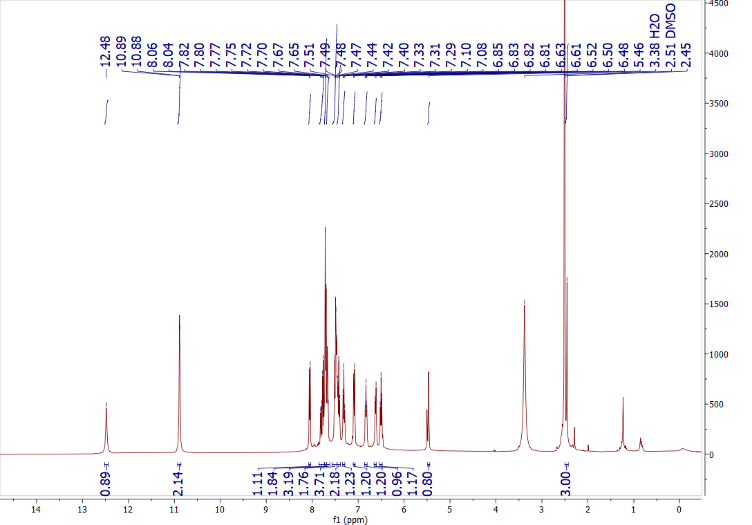
*

# Supplementary Figure 45: ^1^HNMR spectrum of 3-(1H-indol-3-yl)-7'-methyl-1,11-dihydrospiro[benzo[g]pyrazolo[3,4-b]quinoline-4,11'-indeno[1,2-b]quinoxaline]-5,10-dione--methane (C6).

*
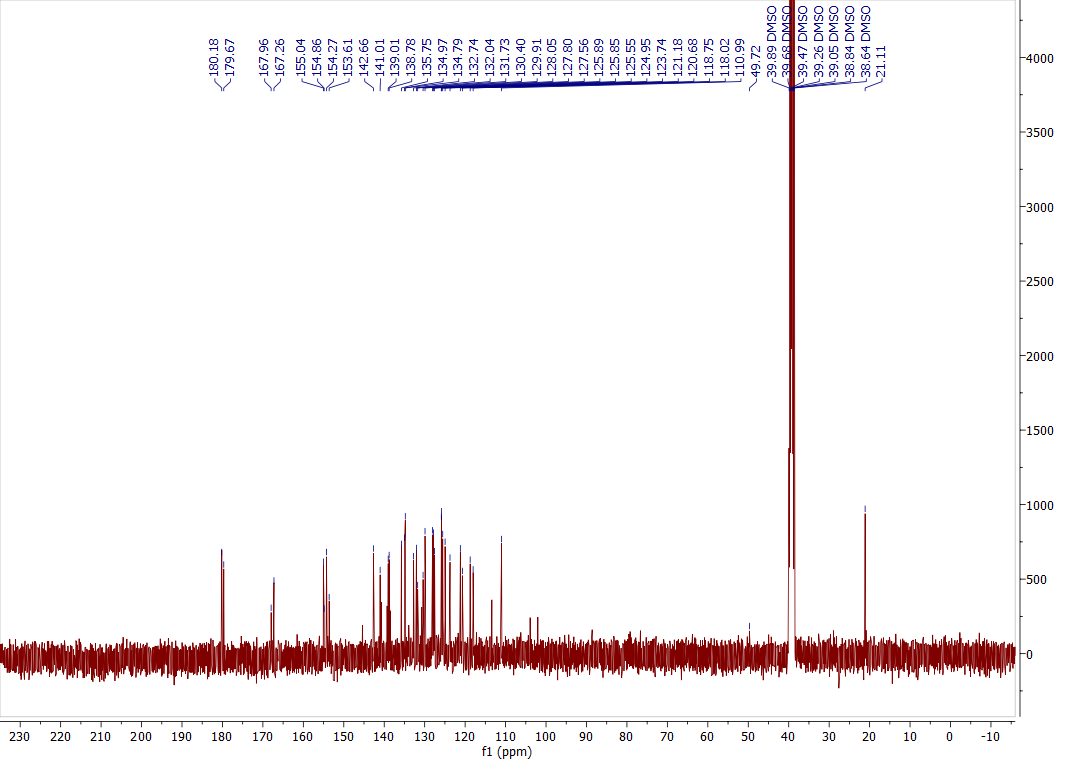
*

# Supplementary Figure 46: ^13^CNMR spectrum of 3-(1H-indol-3-yl)-7'-methyl-1,11-dihydrospiro[benzo[g]pyrazolo[3,4-b]quinoline-4,11'-indeno[1,2-b]quinoxaline]-5,10-dione--methane (C6).

*
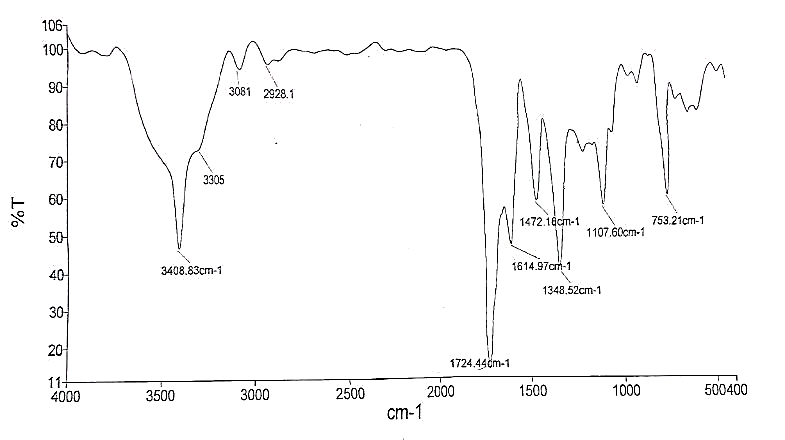
*

# Supplementary Figure 47: FT-IR spectrum of 10-(1H-indol-3-yl)-7,8-dihydro-6H-spiro[chromeno[3,4-b]pyrazolo[4,3-e]pyridine-11,3'-indoline]-2',6-dione (D1).

*
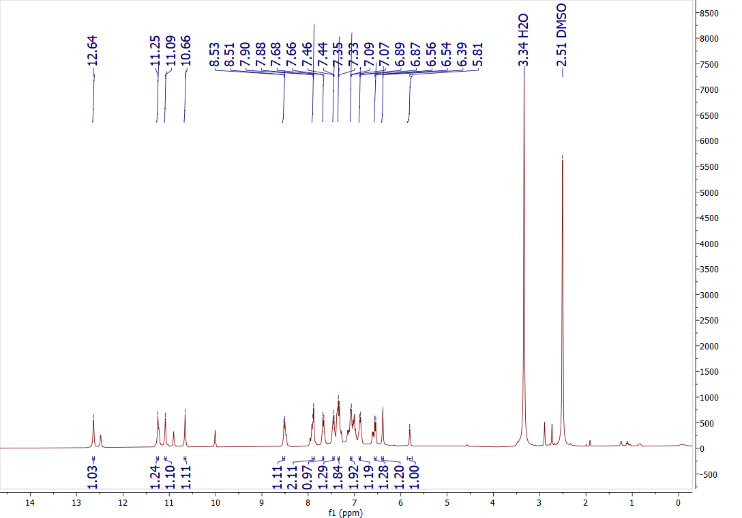
*

# Supplementary Figure 48: ^1^HNMR spectrum of 10-(1H-indol-3-yl)-7,8-dihydro-6H-spiro[chromeno[3,4-b]pyrazolo[4,3-e]pyridine-11,3'-indoline]-2',6-dione (D1).

*
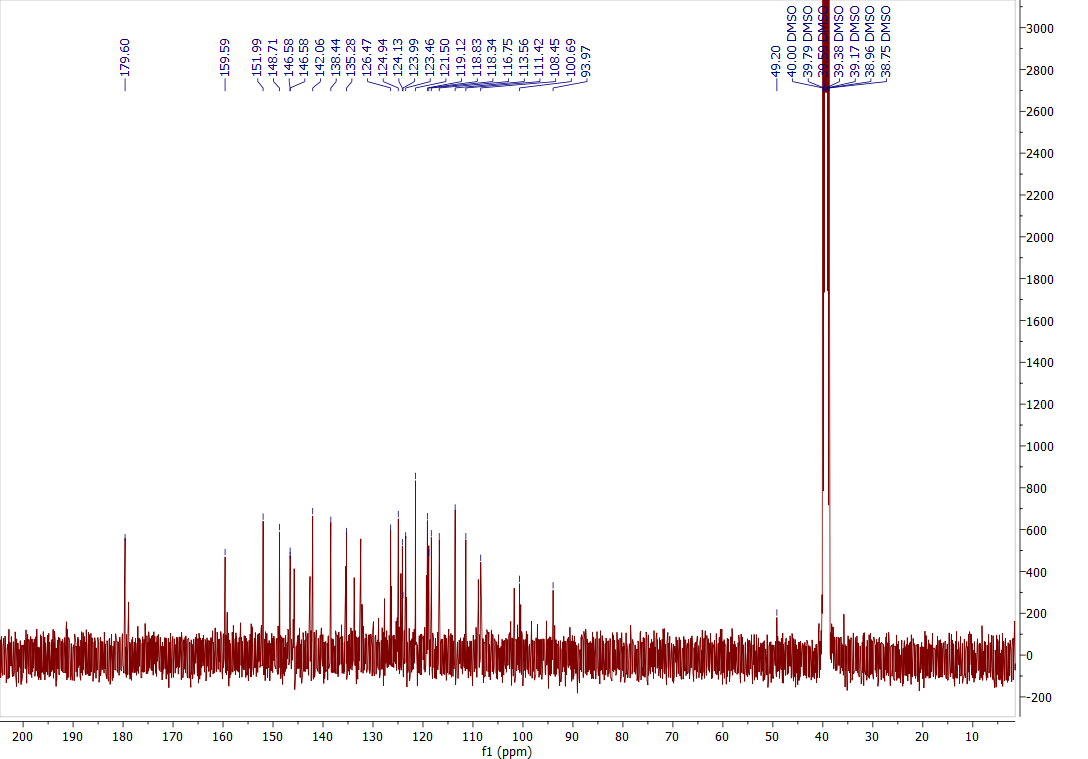
*

# Supplementary Figure 49: ^13^CNMR spectrum of 10-(1H-indol-3-yl)-7,8-dihydro-6H-spiro[chromeno[3,4-b]pyrazolo[4,3-e]pyridine-11,3'-indoline]-2',6-dione (D1).

*
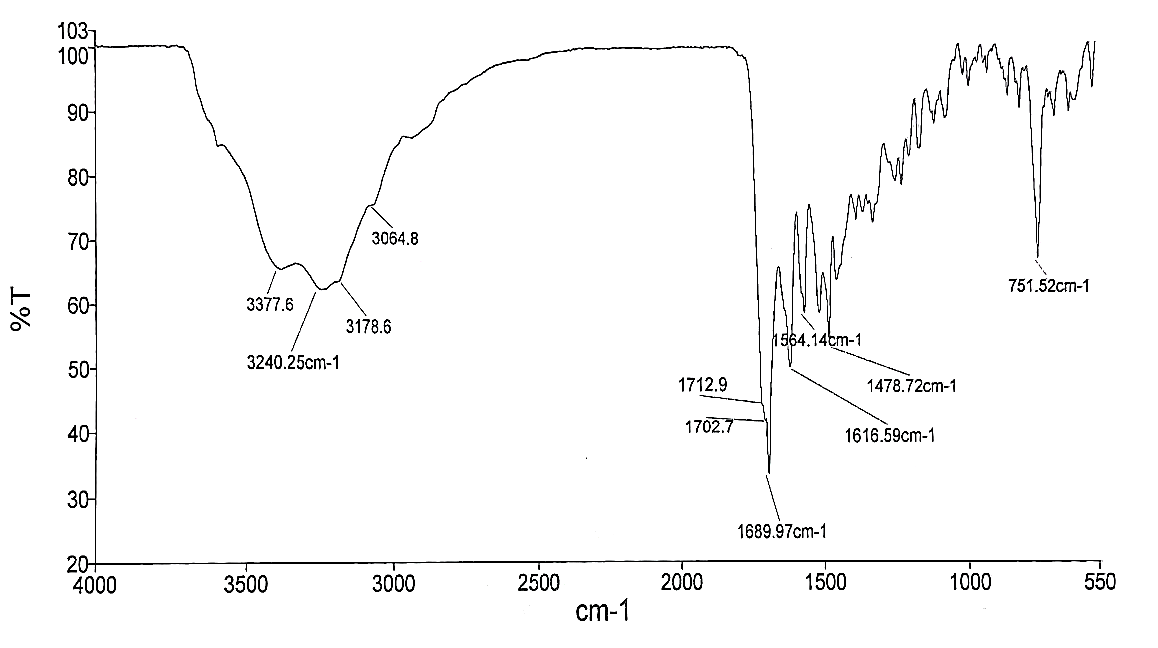
*

# Supplementary Figure 50: FT-IR spectrum of 6'-chloro-10-(1H-indol-3-yl)-7,8-dihydro-6H-spiro[chromeno[3,4-b]pyrazolo[4,3-e]pyridine-11,3'-indoline]-2',6-dione (D2).

*
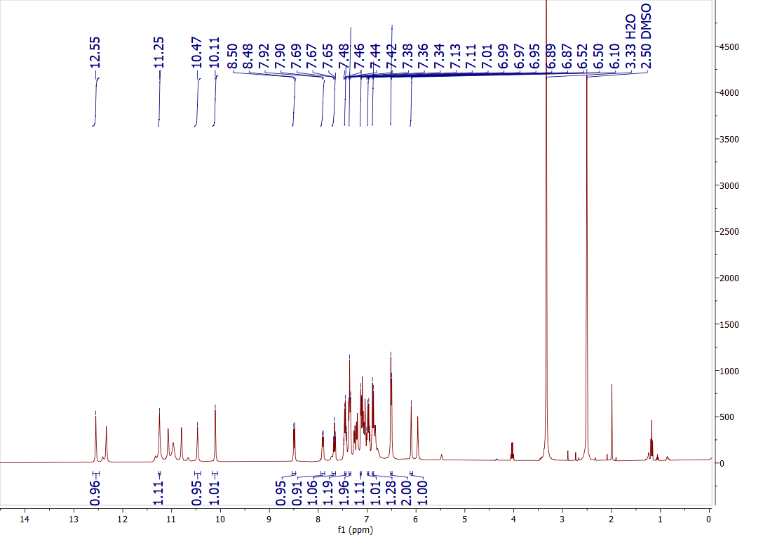
*

# Supplementary Figure 51: ^1^HNMR spectrum of 6'-chloro-10-(1H-indol-3-yl)-7,8-dihydro-6H-spiro[chromeno[3,4-b]pyrazolo[4,3-e]pyridine-11,3'-indoline]-2',6-dione (D2).

*
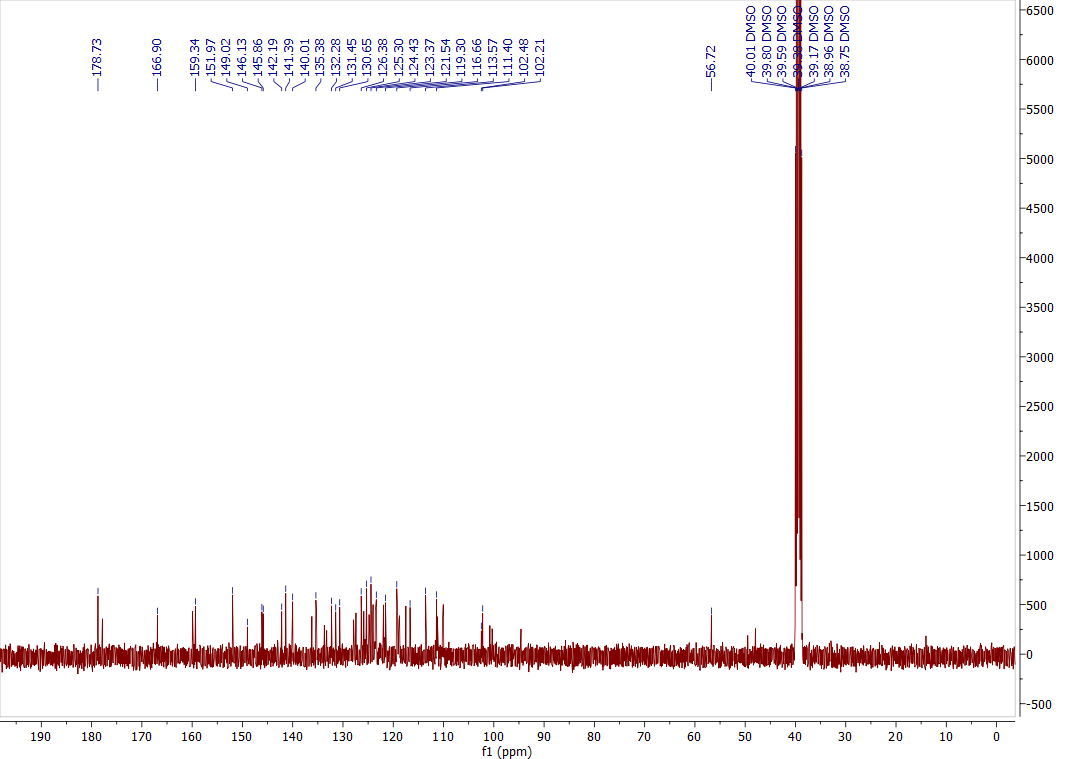
*

# Supplementary Figure 52: ^13^CNMR spectrum of 6'-chloro-10-(1H-indol-3-yl)-7,8-dihydro-6H-spiro[chromeno[3,4-b]pyrazolo[4,3-e]pyridine-11,3'-indoline]-2',6-dione (D2).

*
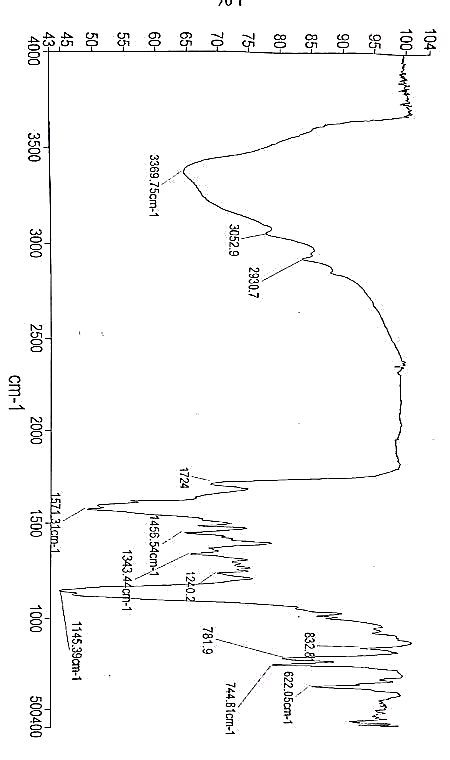
*

# Supplementary Figure 53: FT-IR spectrum of 10'-(1H-indol-3-yl)-7',8'-dihydro-2H,6'H-spiro[acenaphthylene-1,11'-chromeno[3,4-b]pyrazolo[4,3-e]pyridine]-2,6'-dione (D3).

*
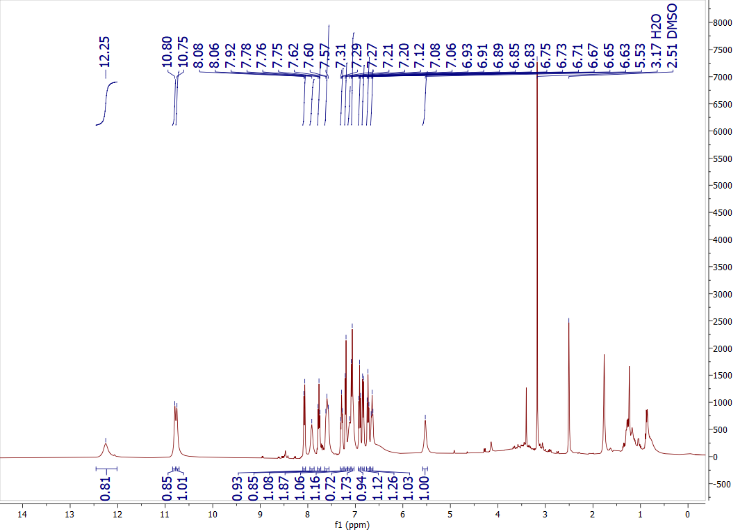
*

# Supplementary Figure 54: ^1^HNMR spectrum of 10'-(1H-indol-3-yl)-7',8'-dihydro-2H,6'H-spiro[acenaphthylene-1,11'-chromeno[3,4-b]pyrazolo[4,3-e]pyridine]-2,6'-dione (D3).

*
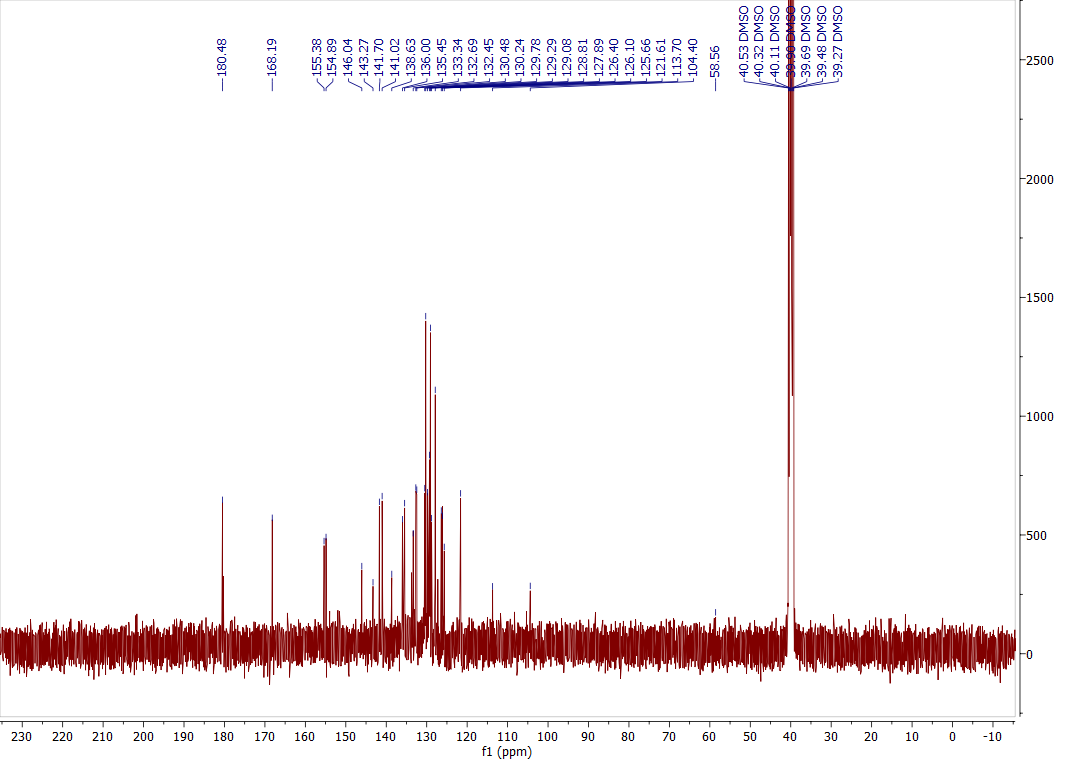
*

# Supplementary Figure 55: ^13^CNMR spectrum of 10'-(1H-indol-3-yl)-7',8'-dihydro-2H,6'H-spiro[acenaphthylene-1,11'-chromeno[3,4-b]pyrazolo[4,3-e]pyridine]-2,6'-dione (D3).
